# Cross-Mating Compatibility and Competitiveness among *Aedes albopictus* Strains from Distinct Geographic Origins - Implications for Future Application of SIT Programs in the South West Indian Ocean Islands

**DOI:** 10.1371/journal.pone.0163788

**Published:** 2016-11-02

**Authors:** David Damiens, Cyrille Lebon, David A. Wilkinson, Damien Dijoux-Millet, Gilbert Le Goff, Ambicadutt Bheecarry, Louis Clément Gouagna

**Affiliations:** 1 Institut de Recherche pour le Développement (IRD), Unité Mixte de Recherche MIVEGEC (IRD 224-CNRS 5290-UM1-UM2), Montpellier, France; 2 IRD La Réunion - Plateforme de Recherche CYROI, Sainte-Clotilde, Reunion Island, France; 3 Université de La Réunion, Unité Mixte de Recherche « Processus Infectieux en Milieu Insulaire Tropical (UMR PIMIT) », INSERM U1187-CNRS9192-IRD249. Plateforme de Recherche CYROI. Ste Clotilde, Saint-Denis, La Réunion, France; 4 Université de La Réunion, Institut Universitaire de Technologie, Département Génie Biologique, Saint Pierre, La Réunion, France; 5 Vector Biology and Control Division, Ministry of Health and Quality of Life, SSR Botanical Garden Rd, Curepipe, Mauritius; International Atomic Energy Agency, AUSTRIA

## Abstract

The production of large numbers of males needed for a sustainable sterile insect technique (SIT) control program requires significant developmental and operational costs. This may constitute a significant economic barrier to the installation of large scale rearing facilities in countries that are undergoing a transition from being largely dependent on insecticide use to be in a position to integrate the SIT against *Aedes albopictus*. Alternative options available for those countries could be to rely on outsourcing of sterile males from a foreign supplier, or for one centralised facility to produce mosquitoes for several countries, thus increasing the efficiency of the mass-rearing effort. However, demonstration of strain compatibility is a prerequisite for the export of mosquitoes for transborder SIT applications. Here, we compared mating compatibility among *Ae*. *albopictus* populations originating from three islands of the South Western Indian Ocean, and assessed both insemination rates and egg fertility in all possible cross-mating combinations. Furthermore, competitiveness between irradiated and non-irradiated males from the three studied strains, and the subsequent effect on female fertility were also examined. Although morphometric analysis of wing shapes suggested phenoptypic differences between *Ae*. *albopictus* strains, perfect reproductive compatibility between them was observed. Furthermore, irradiated males from the different islands demonstrated similar levels of competitiveness and induced sterility when confronted with fertile males from any of the other island populations tested. In conclusion, despite the evidence of inter-strain differences based on male wing morphology, collectively, our results provide a new set of expectations for the use of a single candidate strain of mass-reared sterile males for area-wide scale application of SIT against *Ae*. *albopictus* populations in different islands across the South Western Indian Ocean. Cross-mating competitiveness tests such as those applied here are necessary to assess the quality of mass reared strains for the trans-border application of sterile male release programs.

## Introduction

*Aedes albopictus* (Skuse) (Diptera: Culicidae) is the most abundant *Aedes* species in the islands of the South West Indian Ocean (SWIO). In the major Chikungunya virus epidemic that occurred on many of the SWIO islands between 2005 and 2007 [1–3], *Ae*. *albopictus* was incriminated as the sole vector responsible for viral transmission in Mayotte, La Reunion, the Seychelles, Mauritius, and Rodrigues. *Ae*. *aegypti* was the only vector implicated in the Comoros archipelago, while both *Aedes species* were involved in Madagascar.

In response to these devastating chikungunya outbreaks and motivated by the need to reduce insecticide use for vector control, both La Réunion and Mauritius Islands have launched research efforts to develop and implement the sterile insect technique (SIT) as a component of control strategies against *Ae*. *albopictus*. The SIT is based on the repeated inundation of sterile males to induce sterility in the wild population over a number of generations, and consequently suppress the target pest species [4–5]. This technology has been effectively used for decades against numerous species [6–7] including screwworm [8–10], tsetse fly [11] and Mediterranean fruit fly [12–13] and its potential for mosquito control was shown in early trials in the 1960s and 1970s [14–18]. Proof of principle for the use of genetic control strategies has now been demonstrated in several ongoing programs aimed to reduce wild populations of *Ae*. *albopictus* [19–20], *Ae*. *aegypti* [21], and *Aedes polynesiensis* [22]. In recent years, efforts have also been made to assess the feasibility of the SIT program for *Ae*. *albopictus* control, both in Reunion [23–25] and Mauritius Island [26].

The SIT can be used to effectively suppress isolated vector populations, for example on islands where most immigration of fertile mosquitoes and reinvasion can be controlled. However, the successful implementation of any SIT program relies on sustaining continuous production and the release of a high ratio of sterile-to-fertile males within the target area over a long period of time [18]. The production of such large numbers of males requires the optimization of technical and procedural elements of mass rearing [27–31]. The development of an effective mass-rearing facility poses a number of specific challenges, including careful consideration of different technological requirements, installation costs, logistics and the operational capacity to sustainably support field interventions, as well as sufficient human resource availability for the time-consuming rearing process. Cumulatively, these setup requirements may generate an impractical economic barrier to a number of countries that would otherwise benefit from the SIT in their vector control efforts. One cost effective strategy is outsourcing insect production to an established rearing facility that can supply sterile males to release programmes in other areas [32–34]. Several SIT-based suppression or containment programmes worldwide, e.g. against pink bollworm [35], New World screwworm [36–37] and Mediterranean fruit fly [38–39], have been successfully implemented based on outsourcing components from countries where mass production facilities were already established.

Commonly, this practice consists of producing viable and competitive sterile insects in one country, which are generally shipped as pupae to foreign processing facilities where adults emerge and are processed for release. However, in some situations mating barriers between strains from distinct geographical areas have affected the suitability of allochthon strains of sterile insects obtained from foreign suppliers in place of an autochthon source, for example the South American fruit fly *Anastrepha fraterculus* (Wiedermann) [40]. Across associated literature, the most frequently cited reasons for such mating barriers included, but were not limited to, intraspecific variation in life history traits resulting from differences in selection pressures [41–44], differences in ecological effects on the patterns of wing morphology [45–50], and adult fitness [51–52]. Furthermore, there may be genetic divergence over geographical distance [53–54], and artificial selection associated with rearing conditions that may result in behavioural changes. Assuming sterile males from mass-reared strains were originally fully sexually competitive, and even though intra-species genetic incompatibility is unlikely, careful testing of strain compatibility and competitiveness is a prerequisite for the export of males for the SIT.

In the current study, we present relevant data to assess the potential of an established rearing facility to supply sterile males for the SIT-based control program against *Ae*. *albopictus* populations from across the islands of the SWIO. Specifically, a series of laboratory-cage tests were conducted (1) to assess mating compatibility among three *Ae*. *albopictus* strains from Reunion Island, Mauritius and Seychelles, three isolated Island systems (50–200 km apart), and (2) to evaluate the mating competitiveness of sterile vs. fertile males from the three *Ae*. *albopictus* strains. The absence of mating barriers between populations from different islands would allow for the mass production of a single candidate mosquito strain for use in different island nations.

## Materials and Methods

### Mosquito populations

The *Ae*. *albopictus* strains used in the experiments were originally collected as eggs from Seychelles (Sey), La Réunion (Run) and Mauritius (Mau), three islands in the Indian Ocean region. To produce experimental insect lineages, colonies were established at the insectary of the CYROI (*Cyclotron*, *Reunion Océan Indien*) research platform in March 2015. All three strains were routinely maintained in the same climate-controlled insectary (T: 27 ± 2°C, RH: 75 ± 2%, light: 12L:12D). Adults were continuously given access to a 10% sucrose solution [w/v] in laboratory cages (30x30x30 cm). Females were fed with 2.5 ml of sheep blood through a Parafilm^®^ membrane on the Hemotek^®^ system (PS6 Power Unit, Discovery Workshops, Accrington, Lancashire, United Kingdom) [55]. The use of sheep blood provided by the slaughterhouse for mosquito feeding did not require any particular ethical clearance. Eggs were collected on moist seed germination paper (Seedburo Equipment Company, Illinois, USA) and stored for 8 days in laboratory conditions to allow egg maturation and the synchronization of hatching. Eggs were hatched in larval rearing trays in which dehydrated rabbit food (hay pellet, *Compagnie des Grains du Capricorne*, Le Port, Reunion Island) had been left overnight in water. After 24h, approximately 500 larvae (L1) were added to each tray (30x40 cm) containing 1 litre of water for rearing. They were fed daily with dry pellets composed of 50% rabbit-food and 50% fish-food (Tetramin, Tetra, Germany). Eggs from the F3-4 Run, F8-9 Sey, F10-11 Mau generations were used for experiments. Unless otherwise stated all male and female mosquitoes used in the experiments described below were 2 days old.

### Assessment of inter-strain mating compatibility

Batches of one thousand five hundred eggs from each population were counted into rearing trays (30x40x10 cm). Upon hatching, larvae were reared at a density of ~0.5 larvae/ml, in a controlled climatic chamber (Versatile Environmental Test Chamber MLR-350H, Sanyo) at 29°C and a photoperiod of 12:12 (L:D). They were fed with 5, 7, 7 and 5 ml per tray of a solution at 7.5% (wt:vol) slurry of diet (50% ground rabbit-food and 50% ground fish-food Tetramin, Tetra, Germany) on days 1,2,3 and 4, respectively. Pupae appeared after 5 days of larval development and were collected for experimental purposes. Virgin mosquitoes were obtained by separating females and males as pupae based on terminalia morphology as observed under an optical microscope (Leica MZ6 X25). After sex separation, female pupae and male pupae were allowed to emerge into separate cages and observed to check the accuracy of the separation method. To test the mating compatibility between the three populations, newly emerged males and females were then dispatched in cages at a 1:1 ratio (male:female) for each of the 9 cross-mating combinations between the three strains (3 intra-strain mating and 6 inter-strain mating) (Table 1). All inter-strain mating combinations were carried out simultaneously and were repeated three times. Randomly selected lots of 50 virgin females and 50 virgin males were placed in a 30x30x30 cm cage and allowed to mate for 24h during which they were given access to 10% (w/v) sucrose solution on a cotton wick. At the end of this period, males were removed and females were offered blood over two consecutive days. Blood meals consisted of defibrinated fresh rabbit blood provided through Parafilm^®^ membrane with Hemotek^®^ feeding system. The Hemotek plate was placed on top of the cages for 30 minutes each day. After two days, plastic cups containing 100 ml of water and lined with strips of germination paper were provided in each cage for oviposition. Eggs were subsequently collected and dried for 2 days under ambient laboratory conditions and then allowed a maturation period of 8 days. The number of eggs laid per female was estimated by dividing the observed number of eggs (counted in the lab using an optical microscope, Leica MZ6, x20) from each cross by the number of females alive during the egg laying period. In order to determine the insemination rate in each experimental cage, surviving females were dissected and their spermathecae examined under microscope (Leica MZ6, X40) for the presence of sperm. The eggs from each cross were subsequently allowed to hatch for 24h in hatching solution (as above). The hatching rate was defined as the number of hatched vs. non-hatched eggs. Each cross-mating trial was repeated on three occasions, using different batches of newly emerged mosquitoes.

**Table 1:** Mean insemination rates, egg production per female and egg hatching rates (±SD) in reciprocal cross-mating experiments between *Aedes albopictus* males and females from the three geographical origins.

Male	Female	Insemination rate	Egg production per female	Hatching rate
Run	Run	98.67±2.31	18.38±0.90	94.05±3.36
Run	Sey	98.52±1.29	22.41±4.00	91.22±4.78
Run	Mau	97.57±4.22	25.20±2.35	86.36±8.31
Sey	Run	93.76±5.97	16.07±0.81	81.02±16.43
Sey	Sey	95.77±5.00	16.69±2.57	81.93±17.02
Sey	Mau	98.33±1.45	19.02±10.78	80.67±13.72
Mau	Run	97.69±2.38	16.42±2.06	88.30±15.94
Mau	Sey	100±0.00	23.19±7.60	84.13±12.72
Mau	Mau	97.06±1.23	19.26±4.74	74.51±7.61

Cross-mating experiments between laboratory-reared *Aedes albopictus* males and females from three islands of the SWIO (i.e. Reunion = Run, Seychelles = Sey and Mauritius = Mau) were performed under laboratory conditions.

### Mating Competitiveness of irradiated vs. non-irradiated males

The success of a sterile male *Ae*. *albopictus* transborder eradication program would be critically dependent on their ability of sterile males to effectively compete for mating with local wild males. The purpose of this experiment was to test the ability of sterile males originating from different islands to achieve copulations with females of different origins (Table 2) and to assess the degree of sterility conferred to eggs produced by inseminated females. The procedures for mosquito production and sex separation were identical to those presented above. After sex separation, female pupae and a portion of the male pupae were allowed to emerge into separate adult cages without further treatment to produce non-irradiated males and virgin females. Separate batches of male pupae (aged between 24 and 30 h) from the same larval cohort were randomly retrieved and sterilized with 35 Gy of ionizing radiation (2.35 Gy min^-1^ for 15 min) from a Cs^137^ source (Gammacell IBL 437, Cis Bio International, Germany) at the Blood bank located at the Bellepierre hospital, St Denis de La Réunion. The experiments were scheduled so that males of each of the 3 strains received irradiation treatment and were allowed to compete with non-irradiated males from other strains, all competition experiments being run simultaneously. In total, 21 possible combinations of the three strains were run simultaneously in 30x30x30 cm cages (Table 2). First, a 1:1:1 (non-irradiated male: irradiated male: virgin female) ratio cage was prepared for each of the 9 competition combinations between the three strains (Table 2, lines 1 to 9). Thus, batches of 50 non-irradiated males and 50 irradiated males of the same age but different origins were released in each cage when they were 2 days old. They were allowed to compete for mating with 50 two day old virgin females of the same origin as the non-irradiated males. In addition, for each cross-mating trial two control cages were set up as follows: 1) one “irradiated control” cage into which 50 irradiated males were released along with 50 virgin females of the same strain (9 combinations of different strains at the ratio of 50:0:50), respectively (Table 2, lines 10 to 18) and 2) one “non-irradiated control” cage into which 50 non-irradiated males were released along with 50 virgin females of the same strain (3 strains x 3 controls at the ratio of 0:50:50, Table 2, lines 19 to 21). The mosquitoes were left in the cages and provided with 10% sucrose solution for 2 days, after which females were separated, offered blood on two consecutive days, and allowed to oviposit for 24–48 hours. At the end of this period, the number of eggs was recorded and induced sterility estimated from the hatch rate. The rest of the procedures, egg maturation, counting and hatching, were identical to those used for experiment 1 (described above). The mating experiments were performed under the same conditions as those of routine rearing. The data on hatch rate of each cross was used for the calculation of the competitiveness index, ‘C’ [56].

**Table 2:** Variation of mean insemination rates (±SD) and mean numbers of eggs produced (±SD) for individual females in mating crosses with a 1:1 ratio of irradiated to non-irradiated males either from the same strain or from geographical different strains: Reunion Island (Run), Seychelles (Sey) or Mauritius (Mau).

	Irradiated male (S)	Non irradiated male (N)	Female	Insemination rate	Egg production
1	Run	Run	Run	96.86±2.73	28.67±10.77
2	Run	Sey	Sey	98.20±3.13	23.72±11.99
3	Run	Mau	Mau	100±0.00	28.40±7.68
4	Sey	Run	Run	97.98±3.50	18.38±10.82
5	Sey	Sey	Sey	98.10±3.30	23.20±5.37
6	Sey	Mau	Mau	98.25±3.04	20.61±8.80
7	Mau	Run	Run	100±0.00	24.29±10.26
8	Mau	Sey	Sey	100±0.00	20.56±10.82
9	Mau	Mau	Mau	95.66±7.53	22.99±7.99
10	Run	-	Run	98.89±1.92	20.49±13.42
11	Run	-	Sey	96.40±6.24	23.02±17.70
12	Run	-	Mau	98.81±2.06	23.76±0.19
13	Sey	-	Run	100±0.00	19.28±8.31
14	Sey	-	Sey	99.21±1.37	22.722±8.34
15	Sey	-	Mau	97.86±2.05	21.99±7.77
16	Mau	-	Run	100±0.00	24.71±10.97
17	Mau	-	Sey	98.03±1.75	24.33±13.65
18	Mau	-	Mau	100±0.00	24.17±11.75
19	-	Run	Run	100±0.00	23.95±15.18
20	-	Sey	Sey	99.15±1.48	22.45±5.84
21	-	Mau	Mau	100±0.00	18.59±6.08

Values are means ± Standard deviation

‘C’ was calculated using the observed hatch rates from the fertile (non-irradiated) control (Hn), sterile (irradiated) control (Hs) and treatment (reciprocal cross) groups (Ho) as follows: C = N/S*(Hn-Ho)/(Ho-Hs), where N is the number of non-irradiated males, S is the number of irradiated (or sterile) males. C Values around 1 indicate equivalent mating performance between irradiated and non-irradiated males. The induced sterility (IS), defined as the reduction of fertility of female populations induced by irradiated males, in each treatment group was calculated as: IS = (Hn-Ho)/(Hn-Hs)*100.

For each competition situation in which the three strains of males and females (from Reunion, Mauritius or Seychelles) were mixed, different ‘Hn’ and ‘Hs’ were estimated for the calculation of CI and IS. For example, in the cross between irradiated ♂Run × ♀Sey x non-irradiated ♂Sey (Table 2), the ‘Hn’ from the Sey:Sey non-irradiated control (i.e. non-irradiated ♂Sey × ♀ in Table 2) and the ‘Hs’ from the irradiated cross (i.e. combination 11 in Table 2: irradiated ♂Run x non-irradiated ♂Sey x ♀Run,) were used, while the ‘Ho’ was estimated from the ♂Run: ♀Sey: ♂Sey, i.e, the competition test irradiated ♂ Run × ♀Sey x non-irradiated ♂Sey when non-irradiated males from Reunion island were released together with males and females from the Seychelles strain.

### Wing morphometry

Natural variation in wing shape is known to occur in insect species and influences an individual’s fitness, as for parasitoids [57] or diptera [45–46]. Moreover some geographical variations in wing shape exist in Odonata [58], Lepidoptera [59], Coleoptera [60] and Diptera [47–50]. In order to determine if such a morphological trait difference exists among our tested *Aedes albopictus* strains from the three tested islands, landmark-based geometric morphometric comparisons were performed on wings of the male mosquitoes used in the first experiment described above. Thus, after the mating period, males were collected and killed by freezing. Wings were detached from the thorax, placed on a glass slide and secured with a cover slip. Images of mounted wings were captured using a ScopeTek DCM310 camera fitted on a binocular microscope at 4x magnification (Leica MZ6). Images were further processed using a custom-written MATLAB-based user interface. True scale was calculated using an image of a graticule at the appropriate magnification, and a 1mm scale bar was incorporated into each digital image. Images were cropped and landmarks positioned with the aid of a custom-designed graphical user interface, coded in MATLAB 2012b using the Image Processing Toolkit. Twelve landmark positions were selected, based on previous literature of insects [61–62] and specifically *Aedes* [63, 49] wing morphometry. Procrustes superimposition was optimized over ten iterations, standardizing centroid size to remove location and orientation effects. The horizontal axis of the wing was defined as the line joining average positions of the distal end of the radial branch 3 and the notch between the alula and the posterior margin of the wing, excluding the fringe. Centroid size was calculated for horizontal, vertical and total components as the root sum square of Euclidean distances between landmarks and the centroid [64]. Principle component analysis was performed using covariance matrices generated from the superimposed dataset. ANOVA analyses comparing coordinates of each input data point along the first two principle components were used to test differenced in wing shape. For each principle component, ANOVA analyses allow the detection of significant differences between individuals from each island.

### Parameters recorded and statistical analyses

Prior to running statistical analyses, the data for each treatment cross with the different geographical strains and control groups within each experimental trial were summarized for the following statistics: the percentage of inseminated females in each individual cage, number of eggs laid and percent egg hatch (number of hatched eggs divide by the total number of eggs recorded), and Fried’s competitiveness value (C). Analysis of variance (ANOVA) was used to compare quantitative means between experimental groups. Prior to the analysis, data recorded as percentages were first arcsine—square root transformed while all count data were log-transformed to increase their fit to the normal distribution. Variations in ‘means’ were further examined using the Tukey’s honestly significant difference (HSD) multiple comparison test (post hoc). Strain origin effect on insemination rate, egg production per female and hatching rate were further analysed using a two-way ANOVA. Statistical significance was determined at α = 0.05 level. All statistical analyses described hereafter were performed using Minitab version 16 (Minitab 2000).

## Results

### Assessment of inter-strain mating compatibility

A total number of 2700 *Ae*. *albopictus* (that comprised 1350 females and 1350 males) were used in the 27 (9 combinations among 3 strains x 3 replicates) experimental crosses. The insemination rates (range: 95.7–98.6%), the number of eggs produced by individual females (range: 16.7–19 eggs per female), and the egg hatching rate (81–94% hatched) were not significantly different between the 3 strains (S1 Table). In addition, there was no significant difference in insemination rate, or in the resulting fecundity and egg hatching between intra and inter-strain crosses. On average, irrespective of the origin of females in any inter-strain cross-mating experiment, 93.7–100% of them were effectively inseminated by male individuals from the other populations. There was no significant difference in the proportion of inseminated females between the 6 inter-strain cross-mating trials (ANOVA, F_8,18_ = 0.80, P = 0.61, Table 1). Moreover, the differences in the egg production per female (F_8,18_ = 1.08, P = 0.42) and hatching rate (F_8,18_ = 0.76, P = 0.64) were not statistically significant across all cross-mating trials. Further analysis indicated that in both directions of the reciprocal crosses the origin of males released in the cage did not significantly affect the insemination rate (Two-way ANOVA, F = 0.96, DF = 2, P = 0.40), the number of eggs laid per female (F = 1.98, DF = 2, P = 0.17), or hatching rate (F = 1.28, DF = 2, P = 0.30). Similarly, there was no significant effect of female origin (F = 0.34, DF = 2, P = 0.71; F = 1.44, DF = 2, P = 0.26; F = 1.16, DF = 2, P = 0.33; respectively), or the interaction between male and female origin in any measured outcomes (F = 0.94, DF = 2, P = 0.46; F = 0.44, DF = 2, P = 0.77; F = 0.30, DF = 2, P = 0.87; respectively). Overall, the data from the cross-mating experiments indicate the absence of mating barriers between the three tested strains.

### Mating Competitiveness of irradiated vs. non-irradiated males

**Fig 1:**
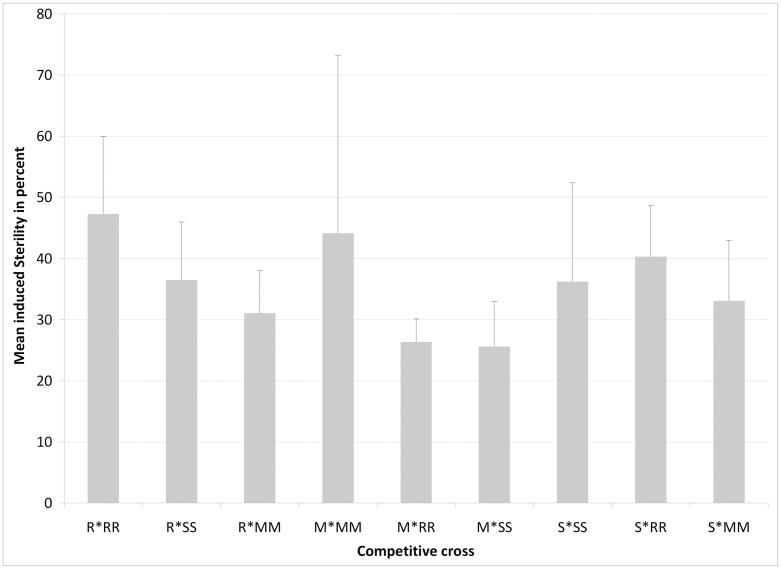
Variation of mean induced sterility (IS) in females under different competitive crosses with a 1:1:1 ratio of irradiated males: non-irradiated males: virgin females from Mauritius (Mau), Seychelles (Sey) or Reunion (Run) *Aedes albopictus* strains. The bars indicate standard deviation. The letter indicates the origin of non-irradiated males and virgin females and the letter with an asterisk indicates the origin of the irradiated males. All inter-strain mating combinations were carried out simultaneously and were repeated three times.

**Fig 2:**
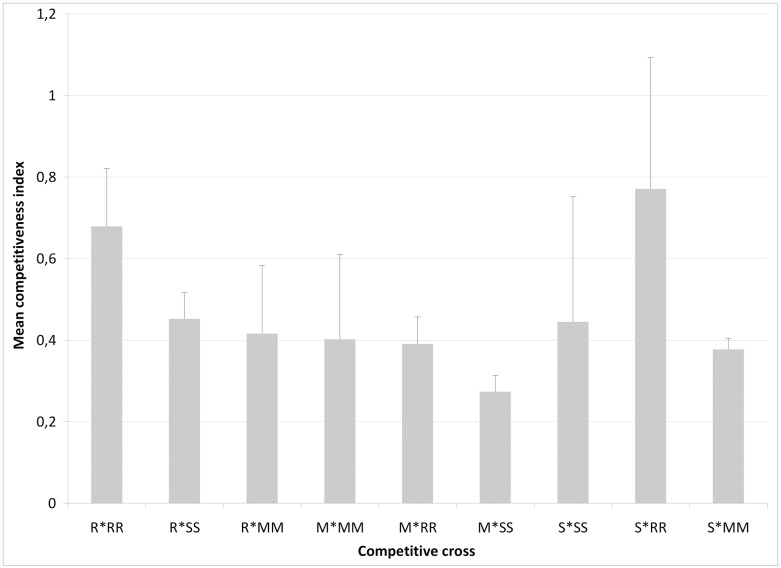
Mean competitiveness index of irradited males in different competitive crosses with a 1:1:1 ratio of irradiated males: non-irradiated males: virgin females from Mauritius (Mau), Seychelles (Sey) or Reunion (Run) *Aedes albopictus* strains. The bars denote standard deviation. The letter indicates the origin of non-irradiated males and virgin females and the letter with an asterisk indicates the origin of the irradiated males.

Given that the cross-mating among the three tested *Aedes albopictus* strains from different islands (Reunion, Mauritius, and Seychelles) were compatible, further cross-mating tests were carried out both (1) to determine the level of sterility induced by irradiated males of each *Ae*. *albopictus* strain in a foreign female population in a non-competitive situation, and (2) to compare competitiveness of irradiated males from each of the three *Ae*. *albopictus* strains when confronted with non-irradiated males from another strain in a 1:1 ratio. As shown in Table 2, there was no significant variation in the proportion of females inseminated (ANOVA, F_20,42_ = 0.67, P = 0.83), or in the egg production per female (F_20,40_ = 0.20, P = 1.00) when all three strains were compared, either in competitive (S2 Table, line 1 to 9) or non-competitive (S2 Table, line 10 to 21) situations. Moreover, there was no significant difference in the degree of Induced Sterility (IS) between the 9 inter-strain mating competitions (S1 Fig). Irrespective of the competition situation or strain origin, the presence of irradiated males strongly decreased the fertility of the female population with an IS that varied from 25% to 47% (S1 Fig). Two way-ANOVA statistical analyses indicated that the origin of the irradiated males (F = 0.54, DF = 2, P = 0.59), or the origin of the couple (non irradiated males and virgin females) (F = 0.36, DF = 2, P = 0.70) had no significant effect on the level of induced sterility (Fig 1). Moreover there is no interaction between the two origins (F = 1.35, DF = 2, P = 0.29). The competitiveness indices (CI) of the irradiated males from different origins are presented in Fig 2. Although irradiated males from Reunion and Seychelles were apparently less competitive in confrontation with non-irradiated males in any competitive situation, pairwise statistical analyses indicated that the relative competitiveness indices involving irradiated males from none of the *Ae*. *albopictus* strains tested were statistically significant (P > 0.05 in all cases). Indeed, there was no significant difference in the CI between mating crosses with non-irradiated males and irradiated males from Reunion Island (F_2,3_ = 2.35, P = 0.24), Mauritius (F_2,3_ = 0.62, P = 0.60) or the Seychelles (F_2,3_ = 1.34, P = 0.38) (Fig 2). This overall trend in mating competitiveness of irradiated males from the three strains was similar for reciprocal competitive crosses among strains.

### Wing morphometry

**Fig 3:**
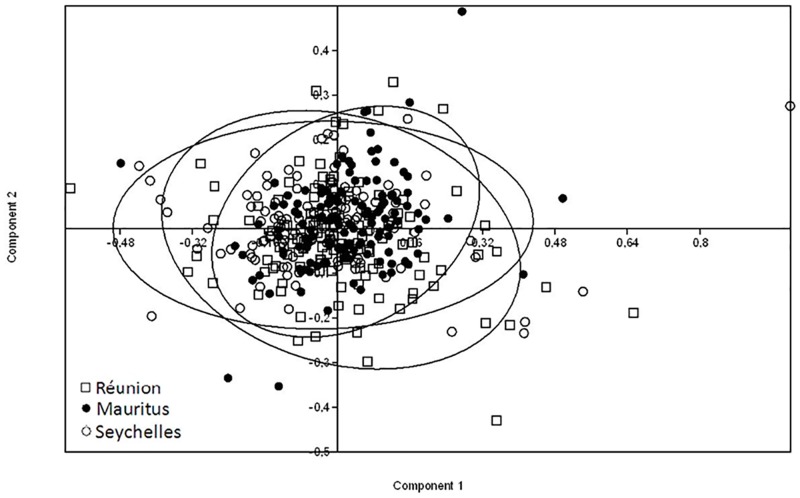
Procrustes analysis of wing morphometric data of individual *Ae albopictus* males from three different islands of the SWOI. Principal component analysis plot of the two principle axes for n = 118, n = 135 and n = 132 male *Ae*. *albopictus* originating from Seychelles, Mauritius and Reunion Island, respectively.

The use of male wing morphometry analyses was a way to immediately differentiate the three strains and to verify that any difference in experimental outcomes between the tested strains was not confounded by variation in the males’ size. A total of 118, 135 and 132 wings were randomly sampled from *Ae*. *albopictus* males derived from the Seychelles, Mauritius and La Reunion strains, respectively, then mounted and measured. Multivariate analysis [principal components analysis (PCA)] was performed on wing landmarks of males from the three strains (Fig 3). In reference to S2 Fig, the first and second principal components together accounted for 53.60% of the total variability in wing shape, and the remaining 17 possible components together explained 99% of the variance of all wing shape. S2 Fig shows that wing morphometric landmarks of the three *Ae*. *albopictus* strains overlapped, but the variations within each strain are different from one another, Furthermore, pairwise comparison of coordinates of each input data point for each principle component reveals statistically significant differences in wing shape in PCA1 between mosquitoes from Mauritius and Reunion (ANOVA, F = 7.81, P<0.001 see post hoc tests in Table 3), and Mauritius and the Seychelles in the PCA2 (ANOVA, F = 10.15, P<0.001), suggesting that the studied mosquitoes may constitute genetically distinct populations.

**Table 3:** Pair-wise Tukey’s Post Hoc analyses of wing shape differences between male *Aedes albopictus* based on the first two statistically significant principle components (PCA1 and PCA2) that discriminate between strains from Seychelles, Mauritius and Reunion Island.

	PCA1			PCA2		
	Mauritius	Reunion	Seychelles	Mauritius	Reunion	Seychelles
Mauritius	-	0,16	P<0.001	-	P<0.001	P<0.001
Reunion	2.59	-	0.08	6.10	-	0.37
Seychelles	5.63	3.04	-	4.19	1.91	-

## Discussion

These mating studies suggest the absence of mating barriers between geographically isolated *Ae*. *albopictus* populations from different islands of the South-western Indian Ocean (SWIO). Males from each island were equally able to copulate with and inseminate females from any other island. Indeed, intra-strain crosses resulted in similar insemination rates, egg production per female and egg hatching rates as those recorded in inter-strain crosses. Such an absence of mating incompatibility between different geographical strains has already been observed in Italy (for example, between *Ae*. *albopictus* strains from Pinerolo, Rimini, Cesena, Bologna and Matera) [65]. However, the presence of *Aedes albopictus* in Italy is due to a recent introduction in the Veneto region, via the importation of used tires from the United States [66, 67]. The absence of geographical barriers as well as the relatively short time scale over which any genetic adaptation will have occurred in these European mosquito populations may account for the observed level of strain compatibility, but in older and more insular systems such as those of the SWIO the same cannot be assumed to be true. In the current study statistical analysis indicated that there were inter-strain differences based on male wing shapes, chiefly between Mauritius *Ae*. *albopictus* strain and two others strains. However, further analyses showed that such variability in morphological traits (wing size) was not associated with mating success obtained in inter-population reciprocal cross mating experiments. In fact, in a study examining intraspecific variations among *Aedes aegypti* populations using landmark*-*based geometric morphometrics, Sendaydiego et al (49) have shown that substantial heterogeneity in wing morphology may exist within mosquito populations from a single locality. The shape differences of a morphological feature in conspecific populations may represent a result of random evolutionary processes. Although many life history traits and mating behaviours may theoretically influence the chance of mating success between mosquito strains, under the current experimental conditions, our results suggest that differences in morphological traits (wing shape) may not be linked to mating or reproductive barriers.

Previous genetic analyses of *Aedes albopictus* strains from Madagascar and Reunion Island indicated that there was no noticeable genetic difference between strains or to those from geographically distant strains originating from the USA or France [68]. Phylogenetic studies in *Ae*. *albopictus* had already shown little variation among various globally distributed populations in newly invaded countries, as a result of its recent worldwide expansion [68–73]. Based on phylogenetic analyses of the mtDNA-CO1 sequences, Delatte et al [74] hypothesized that *Ae*. *albopictus* populations on the islands of the SWIO are monophyletic lineages with at least two waves of invasion. The first one probably occurred dozens of centuries ago alongside the human colonization of Madagascar [75], Mauritius and Reunion Island are suspected to have been colonised in more recent decades. Frequent trade connections between SWIO islands have possibly facilitated movements of *Ae*. *albopictus* (as quiescent eggs, larvae and pupae in fresh water supplies on boats, or even as adults), as during the spice trade in the 17–18^th^ centuries [76]. Beside the recognized eco-geographical distribution pattern and phenotypic variations among *Ae*. *albopictus* populations across different SWIO islands, our results further suggest that they could constitute a regional structure of spatially separated populations of the same species without any noticeable consequences of reproductive isolation.

The presented study involved cross-mating in laboratory cages. The applicability of the presented competitiveness index estimates to natural habitats is uncertain, and should be further tested in large cage or field conditions to bring greater certainty in predicting the effects of the SIT strategies on *Ae*. *albopictus* populations of the SWIO.

Caution should be taken when employing a single candidate sterile male mosquito strain for planning and implementing SIT programs at large scales. A successful sterile male release strategy requires appreciable suppression of fertility from mating between irradiated males and wild females. This condition can be met only if sterilization does not affect sexual competitiveness, and when ratios of irradiated to wild males of at least 5:1, and preferably higher, are released to overcome the natural rate of growth of the wild populations [77]. In the present study male sterility in all tested *Ae*. *albopictus* strains was achieved through irradiation of laboratory reared pupae at a dose of 35 Gy. This radiation dose represents the best compromise between the expected level of sterility and mating competitiveness. Consistent with evidence from our previous laboratory and semi-field investigations [78, 24], the radiation dose (35 Gy) currently applied to sterilize *Ae*. *albopictus* males is expected to induce 95–97% sterility. The use of an irradiation dose that does not lead to full sterility poses a challenge to the use of these males as a strategy for control of wild *Ae*. *albopictus* between different countries. Release of sub-sterile male mosquitoes may lead to the production of a small number of offspring from the introduced population. If we assume that *Ae*. *albopictus* populations of the islands of SWIO are monophyletic lineages based on phylogenetic analyses of the mtDNA-CO1 sequences [74], such mating will have no impact on native population gene pool, however, such introductions may be considered as a biosecurity risk by some countries regulations for conservation.

Advances in technology used to generate full sterility without influencing the competitiveness of sterile male mosquitoes would improve efficiencies and reduce the risks of area-wide SIT interventions against *Ae*. *albopictus* populations. An interesting approach to avoid the residual sterility of *Ae*. *albopictus* males would be to combine SIT with incompatible insect technique (IIT) [79, 80]. The IIT is similar to SIT except that instead of releasing irradiated males, males infected (naturally or artificially) with Wolbachia are released. The presence of Wolbachia in these males may induce cytoplasmic incompatibility (CI) leading to embryonic mortality in offspring that result from mating between a Wolbachia-infected male and a female which is either uninfected or infected with a different Wolbachia strain [81, 82]. A proof-of-concept of a combined SIT/IIT strategy in suppressing natural *Aedes albopictus* populations is currently ongoing in Guangzhou, China (Xi Zhiyong, personal comm.). In this approach, the use wolbachia infected mosquitoes combined with low dose irradiation allow the full sterilisation of males [83–85], while insuring the sterility of females and preventing accidental population replacement [83].

## Conclusions

Currently there is a compelling commitment to SIT development and its regional implementation against *Aedes albopictus*, arguably the vector species that poses the greatest threat to human health in the islands of the SWIO. At the same time, area-wide vector control including SIT must have reliable supply of sterile insects, which implies expensive operations with numerous logistical challenges. Critical decisions need to be made concerning the most appropriate and effective way to implement the SIT strategy and how to share resources regionally. Our findings demonstrate that sterilized males from a single strain of *Ae*. *albopictus* could be used to control distinct geographic populations across Mauritius, the Seychelles and Reunion Islands.

The sterile males of each strain of *Ae*. *albopictus* from individual islands seem to provide efficient competition when confronted with fertile males of differing geographical origins in all tested scenarios. Other experiments with additional strains from other countries such as Madagascar, Mayotte, Comoros, or perhaps other nearby African countries would be needed to determine if this finding could apply on a larger, international scale. The development of a single mass-rearing facility for an entire region could allow a significant reduction in the financial investment required to implement effective SIT control strategies for multiple countries, by limiting the costs associated with construction of the mass rearing facility, and honing the expertise required for the mass production of irradiated male pupae [32–34].

## Supporting Information

S1 Fig Mean induced sterility in females under different competitive crosses with a 1:1:1 ratio of irradiated males: non-irradiated males: virgin females from Mauritius (Mau), Seychelles (Sey) or Reunion (Run) *Aedes albopictus* strains. (PDF)

S2 Fig Wing morphometric data of individual *Ae albopictus* males from three different islands of the SWOI: Reunion Island (Run), Seychelles (Sey) or Mauritius (Mau). (PDF)

S1 Table Mating compatibility: Mean insemination rates, egg production per female and egg hatching rates in reciprocal cross-mating experiments between laboratory-reared *Aedes albopictus* males and females from three islands of the SWIO (i.e. Reunion = Run, Seychelles = Sey and Mauritius = Mau). (PDF)

S2 Table Mean insemination rates and mean numbers of eggs produced by individual females in mating crosses with a 1:1 ratio of irradiated to non-irradiated males either from the same strain or from geographical different strains: Reunion Island (Run), Seychelles (Sey) or Mauritius (Mau). (PDF)
