# Supplementary material for: Cross-Mating Compatibility and Competitiveness among Aedes albopictus Strains from Distinct Geographic Origins - Implications for Future Application of SIT Programs in the South West Indian Ocean Islands
Source: PLoS One. 2016 Nov 2;11(11):e0163788. doi: 10.1371/journal.pone.0163788 (PMC5091895; doi:10.1371/journal.pone.0163788)
Supplement: S1 Fig — (PDF) [file pone.0163788.s001.pdf]

replicate	Ratio	mating	number of collected females	egg number	hatched egg number	hatch rate	nb of egg/female
2	1_1_1	Run*_Run_Run	48	927	527	56,85005394	19,3125
2	1_1_1	Sey*_Run_Run	38	708	323	45,62146893	18,63157895
2	1_1_1	Mau*_Run_Run	45	882	594	67,34693878	19,6
2	1_1_1	Run*_Sey_Sey	46	417	251	60,19184652	9,065217391
2	1_1_1	Sey*_Sey_Sey	46	919	482	52,44831338	19,97826087
2	1_1_1	Mau*_Sey_Sey	45	705	477	67,65957447	15,66666667
2	1_1_1	Run*_Mau_Mau	48	731	444	60,73871409	15,22916667
2	1_1_1	Sey*_Mau_Mau	49	1052	597	56,74904943	21,46938776
2	1_1_1	Mau*_Mau_Mau	47	763	488	63,95806029	16,23404255
2	1_0_1	Run*_Run	49	537	14	2,60707635	10,95918367
2	1_0_1	Run*_Sey	50	751	58	7,723035952	15,02
2	1_0_1	Run*_Mau	48	1134	36	3,174603175	23,625
2	1_0_1	Sey*_Run	45	751	22	2,92942743	16,68888889
2	1_0_1	Sey*_Sey	48	698	9	1,289398281	14,54166667
2	1_0_1	Sey*_Mau	50	652	22	3,374233129	13,04
2	1_0_1	Mau*_Run	50	714	46	6,442577031	14,28
2	1_0_1	Mau*_Sey	40	678	39	5,752212389	16,95
2	1_0_1	Mau*_Mau	43	682	63	9,237536657	15,86046512
2	0_1_1	Run_Run	51	732	646	88,25136612	14,35294118
2	0_1_1	Sey_Sey	45	723	624	86,30705394	16,06666667
2	0_1_1	Mau_Mau	41	928	723	77,90948276	22,63414634
3	1_1_1	Run*_Run_Run	49	1982	1103	55,65085772	40,44897959
3	1_1_1	Sey*_Run_Run	41	1534	975	63,55932203	37,41463415
3	1_1_1	Mau*_Run_Run	49	1559	1069	68,56959589	31,81632653
3	1_1_1	Run*_Sey_Sey	43	1301	762	58,57033051	30,25581395
3	1_1_1	Sey*_Sey_Sey	50	1470	978	66,53061224	29,4
3	1_1_1	Mau*_Sey_Sey	47	1446	963	66,59751037	30,76595745
3	1_1_1	Run*_Mau_Mau	54	1913	1078	56,35128071	35,42592593
3	1_1_1	Sey*_Mau_Mau	45	1390	876	63,02158273	30,88888889
3	1_1_1	Mau*_Mau_Mau	48	1527	871	57,03994761	31,8125
3	1_0_1	Run*_Run	43	1541	89	5,775470474	35,8372093
3	1_0_1	Run*_Sey	42	1819	87	4,782847719	43,30952381

3	1_0_1	Run*_Mau		1568	62	3,954081633	
3	1_0_1	Sey*_Run	50	1429	94	6,578026592	28,58
3	1_0_1	Sey*_Sey	51	1142	61	5,34150613	22,39215686
3	1_0_1	Sey*_Mau	52	1401	49	3,497501784	26,94230769
3	1_0_1	Mau*_Run	51	1844	171	9,273318872	36,15686275
3	1_0_1	Mau*_Sey	47	1884	192	10,1910828	40,08510638
3	1_0_1	Mau*_Mau		1530	113	7,385620915	
3	0_1_1	Run_Run	47	1948	1841	94,50718686	41,44680851
3	0_1_1	Sey_Sey	50	1187	955	80,45492839	23,74
3	0_1_1	Mau_Mau	50	1077	908	84,3082637	21,54
4	1_1_1	Run*_Run_Run	16	420	164	39,04761905	26,25
4	1_1_1	Sey*_Run_Run	41	620	388	62,58064516	15,12195122
4	1_1_1	Mau*_Run_Run	23	777	596	76,70527671	33,7826087
4	1_1_1	Run*_Sey_Sey	28	443	208	46,95259594	15,82142857
4	1_1_1	Sey*_Sey_Sey	43	870	405	46,55172414	20,23255814
4	1_1_1	Mau*_Sey_Sey	31	477	297	62,26415094	15,38709677
4	1_1_1	Run*_Mau_Mau	27	600	381	63,5	22,22222222
4	1_1_1	Sey*_Mau_Mau	45	419	234	55,84725537	9,311111111
4	1_1_1	Mau*_Mau_Mau	27	565	193	34,15929204	20,92592593
4	1_0_1	Run*_Run	34	499	18	3,607214429	14,67647059
4	1_0_1	Run*_Sey	41	440	9	2,045454545	10,73170732
4	1_0_1	Run*_Mau	30	717	49	6,834030683	23,9
4	1_0_1	Sey*_Run	43	541	22	4,066543438	12,58139535
4	1_0_1	Sey*_Sey	50	1561	101	6,470211403	31,22
4	1_0_1	Sey*_Mau	48	1248	94	7,532051282	26
4	1_0_1	Mau*_Run	24	569	85	14,93848858	23,70833333
4	1_0_1	Mau*_Sey	35	559	77	13,7745975	15,97142857
4	1_0_1	Mau*_Mau	21	682	107	15,68914956	32,47619048
4	0_1_1	Run_Run	27	433	412	95,15011547	16,03703704
4	0_1_1	Sey_Sey	37	1019	887	87,04612365	27,54054054
4	0_1_1	Mau_Mau	25	290	274	94,48275862	11,6
