# Supplementary material for: Cross-Mating Compatibility and Competitiveness among Aedes albopictus Strains from Distinct Geographic Origins - Implications for Future Application of SIT Programs in the South West Indian Ocean Islands
Source: PLoS One. 2016 Nov 2;11(11):e0163788. doi: 10.1371/journal.pone.0163788 (PMC5091895; doi:10.1371/journal.pone.0163788)
Supplement: S2 Fig — (PDF) [file pone.0163788.s002.pdf]

InsectID	Species	Side	Site	1_to_14	1_to_15	1_to_16
MAU_01	Aedes Albopictus	Right	Maurice	1,46191645	1,08063255	1,08643748
MAU_02	Aedes Albopictus	Right	Maurice	1,4765928	1,05879059	1,05437008
MAU_03	Aedes Albopictus	Right	Maurice	1,47009105	1,07060289	1,08330675
MAU_04	Aedes Albopictus	Right	Maurice	1,46158738	1,04922706	1,05091108
MAU_05	Aedes Albopictus	Right	Maurice	1,31101878	0,98866538	0,99739328
MAU_06	Aedes Albopictus	Right	Maurice	1,44708205	1,04469187	1,06471456
MAU_07	Aedes Albopictus	Right	Maurice	1,46229433	1,07086037	1,07827927
MAU_08	Aedes Albopictus	Right	Maurice	1,47264471	1,07947284	1,09728688
MAU_09	Aedes Albopictus	Right	Maurice	1,47762583	1,06452948	1,07025971
MAU_11	Aedes Albopictus	Right	Maurice	1,50179872	1,08225614	1,08010967
MAU_12	Aedes Albopictus	Right	Maurice	1,47155395	1,08093064	1,08733766
MAU_13	Aedes Albopictus	Right	Maurice	1,45477762	1,04230709	1,05204543
MAU_14	Aedes Albopictus	Right	Maurice	1,5896621	1,06742413	1,08297505
MAU_15	Aedes Albopictus	Right	Maurice	1,35251903	1,04826294	1,053234
MAU_16	Aedes Albopictus	Right	Maurice	1,45118589	1,03604793	1,06499588
MAU_17	Aedes Albopictus	Right	Maurice	1,44022045	1,04610589	1,05577788
MAU_18	Aedes Albopictus	Right	Maurice	1,45049626	1,0574103	1,05861112
MAU_19	Aedes Albopictus	Right	Maurice	1,42075064	1,07508022	1,0788814
MAU_20	Aedes Albopictus	Right	Maurice	1,48136442	1,08386417	1,09211023
MAU_21	Aedes Albopictus	Right	Maurice	1,40229486	1,05316652	1,06431108
MAU_22	Aedes Albopictus	Right	Maurice	1,4744755	1,10554445	1,11249751
MAU_23	Aedes Albopictus	Right	Maurice	1,45353934	1,04269471	1,05378623
MAU_24	Aedes Albopictus	Right	Maurice	1,51563198	1,0563739	1,06308658
MAU_25	Aedes Albopictus	Right	Maurice	1,44783757	1,05307973	1,06796579
MAU_26	Aedes Albopictus	Right	Maurice	1,45232134	1,08285396	1,08707009
MAU_27	Aedes Albopictus	Right	Maurice	1,47638696	1,07662622	1,08669443
MAU_28	Aedes Albopictus	Right	Maurice	1,49901964	1,0670227	1,08251738
MAU_29	Aedes Albopictus	Right	Maurice	1,48218845	1,08412268	1,09103135
MAU_30	Aedes Albopictus	Right	Maurice	1,4442438	1,07144392	1,08942209
MAU_31	Aedes Albopictus	Right	Maurice	1,47809604	1,04889411	1,06988916
MAU_32	Aedes Albopictus	Right	Maurice	1,38387588	1,07463818	1,08227081
MAU_33	Aedes Albopictus	Right	Maurice	1,48235251	1,05743602	1,05928296
MAU_34	Aedes Albopictus	Right	Maurice	1,3877088	1,08144412	1,06853075
MAU_35	Aedes Albopictus	Right	Maurice	1,53327066	1,05228062	1,06696305
MAU_36	Aedes Albopictus	Right	Maurice	1,45654337	1,08027232	1,07116334
MAU_37	Aedes Albopictus	Right	Maurice	1,5104455	1,08048512	1,09135612
MAU_38	Aedes Albopictus	Right	Maurice	1,4805414	1,05620246	1,06236577
MAU_39	Aedes Albopictus	Right	Maurice	1,45152462	1,08448496	1,07998016
MAU_40	Aedes Albopictus	Right	Maurice	1,43141054	1,07115666	1,07821014
MAU_41	Aedes Albopictus	Right	Maurice	1,43789421	1,07739065	1,07797771
MAU_42	Aedes Albopictus	Right	Maurice	1,48291332	1,07378034	1,08096722
MAU_43	Aedes Albopictus	Right	Maurice	1,49184499	1,03114151	1,04103946
MAU_44	Aedes Albopictus	Right	Maurice	1,48731663	1,0360722	1,04891725
MAU_45	Aedes Albopictus	Right	Maurice	1,49005306	1,0572658	1,06637349
MAU_46	Aedes Albopictus	Right	Maurice	1,48684338	1,06001669	1,0763405
MAU_47	Aedes Albopictus	Right	Maurice	1,4532298	1,04939386	1,05873486
MAU_48	Aedes Albopictus	Right	Maurice	1,49977251	1,05901444	1,09637998
MAU_49	Aedes Albopictus	Right	Maurice	1,47348455	1,04969195	1,03368233
MAU_50	Aedes Albopictus	Right	Maurice	1,49628211	1,09333027	1,08171654

MAU_51	Aedes Albopictus	Right	Maurice	1,46929225	1,06696264	1,07768776
MAU_52	Aedes Albopictus	Right	Maurice	1,45633977	1,05286102	1,06202237
MAU_53	Aedes Albopictus	Right	Maurice	1,2755966	1,02737815	1,0341658
MAU_54	Aedes Albopictus	Right	Maurice	1,43274426	1,01727723	1,01619105
MAU_55	Aedes Albopictus	Right	Maurice	1,45444994	1,03670587	1,0499976
MAU_56	Aedes Albopictus	Right	Maurice	1,45875411	1,06833301	1,08543356
MAU_57	Aedes Albopictus	Right	Maurice	1,47985964	1,0492337	1,09097416
MAU_58	Aedes Albopictus	Right	Maurice	1,47208926	1,04447008	1,05283775
MAU_59	Aedes Albopictus	Right	Maurice	1,48294445	1,06821935	1,09154515
MAU_60	Aedes Albopictus	Right	Maurice	1,42492063	1,07059063	1,07127443
MAU_61	Aedes Albopictus	Right	Maurice	1,45906173	1,06027812	1,0684749
MAU_62	Aedes Albopictus	Right	Maurice	1,45283453	1,0765905	1,0838899
MAU_63	Aedes Albopictus	Right	Maurice	1,47043393	1,09107006	1,09754836
MAU_64	Aedes Albopictus	Right	Maurice	1,43686505	1,06621268	1,07209544
MAU_65	Aedes Albopictus	Right	Maurice	1,59139232	1,08695756	1,08775122
MAU_66	Aedes Albopictus	Right	Maurice	1,46052687	1,04377009	1,07762295
MAU_67	Aedes Albopictus	Right	Maurice	1,45254631	1,04999275	1,08557505
MAU_68	Aedes Albopictus	Right	Maurice	1,50812752	1,080545	1,09731876
MAU_69	Aedes Albopictus	Right	Maurice	1,48116063	1,085339	1,09711805
MAU_70	Aedes Albopictus	Right	Maurice	1,45500624	1,0761732	1,08737796
MAU_71	Aedes Albopictus	Right	Maurice	1,48062187	1,04135033	1,06329375
MAU_72	Aedes Albopictus	Right	Maurice	1,43515203	1,06390304	1,06469599
MAU_73	Aedes Albopictus	Right	Maurice	1,45058966	1,09660454	1,11746867
MAU_74	Aedes Albopictus	Right	Maurice	1,49847871	1,10229295	1,1201525
MAU_75	Aedes Albopictus	Right	Maurice	1,48744789	1,08901189	1,10846715
MAU_76	Aedes Albopictus	Right	Maurice	1,45689342	1,04681809	1,05720018
MAU_77	Aedes Albopictus	Right	Maurice	1,49246528	1,07304376	1,08159873
MAU_78	Aedes Albopictus	Right	Maurice	1,4184921	1,02847408	1,049532
MAU_79	Aedes Albopictus	Right	Maurice	1,46165302	1,09838743	1,09303236
MAU_80	Aedes Albopictus	Right	Maurice	1,45887099	1,02548578	1,03904501
MAU_81	Aedes Albopictus	Right	Maurice	1,46872696	1,06877805	1,07950633
MAU_82	Aedes Albopictus	Right	Maurice	1,51396983	1,06928674	1,08213516
MAU_83	Aedes Albopictus	Right	Maurice	1,48103023	1,03473333	1,05358349
MAU_84	Aedes Albopictus	Right	Maurice	1,46558641	1,04845953	1,08132064
MAU_85	Aedes Albopictus	Right	Maurice	1,47787066	1,06361962	1,095421
MAU_86	Aedes Albopictus	Right	Maurice	1,46412442	1,05168202	1,07520754
MAU_87	Aedes Albopictus	Right	Maurice	1,47862011	1,16425906	1,19287375
MAU_88	Aedes Albopictus	Right	Maurice	1,43561302	1,05685045	1,06132032
MAU_91	Aedes Albopictus	Right	Maurice	1,45627724	1,05152956	1,06878569
MAU_92	Aedes Albopictus	Right	Maurice	1,45796661	1,07459714	1,09901957
MAU_93	Aedes Albopictus	Right	Maurice	1,5099462	1,06790684	1,09379202
MAU_102	Aedes Albopictus	Right	Maurice	1,49108115	1,02770287	1,07195198
MAU_103	Aedes Albopictus	Right	Maurice	1,487018	1,07800369	1,08968503
MAU_104	Aedes Albopictus	Right	Maurice	1,49228923	1,05391492	1,09059836
MAU_105	Aedes Albopictus	Right	Maurice	1,52039015	1,08962442	1,12835544
MAU_107	Aedes Albopictus	Right	Maurice	1,47402183	1,03650201	1,08170096
MAU_108	Aedes Albopictus	Right	Maurice	1,45837543	1,066267	1,07396315
MAU_121	Aedes Albopictus	Right	Maurice	1,418729	1,04435423	1,04482079
MAU_122	Aedes Albopictus	Right	Maurice	1,45730181	1,07080665	1,08177245
MAU_123	Aedes Albopictus	Right	Maurice	1,45044347	1,09193452	1,09663406

MAU_124	Aedes Albopictus	Right	Maurice	1,49371094	1,03961145	1,04828799
MAU_125	Aedes Albopictus	Right	Maurice	1,44595062	0,95415143	0,99001988
MAU_126	Aedes Albopictus	Right	Maurice	1,50456514	1,04165608	1,05626949
MAU_127	Aedes Albopictus	Right	Maurice	1,43430303	1,05251913	1,0646389
MAU_128	Aedes Albopictus	Right	Maurice	1,46479156	1,06906621	1,0931842
MAU_129	Aedes Albopictus	Right	Maurice	1,3976113	1,0669504	1,07418544
MAU_130	Aedes Albopictus	Right	Maurice	1,45227435	1,04698096	1,05237895
MAU_131	Aedes Albopictus	Right	Maurice	1,47129527	1,06560141	1,07402079
MAU_132	Aedes Albopictus	Right	Maurice	1,46228117	1,04385088	1,04781646
MAU_133	Aedes Albopictus	Right	Maurice	1,49203515	1,07149091	1,08118207
MAU_134	Aedes Albopictus	Right	Maurice	1,45605184	1,07561911	1,09842104
MAU_135	Aedes Albopictus	Right	Maurice	1,48076169	1,08522075	1,0997537
MAU_136	Aedes Albopictus	Right	Maurice	1,42398301	1,04932471	1,07242815
MAU_137	Aedes Albopictus	Right	Maurice	1,47739435	1,06951721	1,08114555
MAU_138	Aedes Albopictus	Right	Maurice	1,47023141	1,07048859	1,09624896
MAU_139	Aedes Albopictus	Right	Maurice	1,48540705	1,03391024	1,04461655
MAU_140	Aedes Albopictus	Right	Maurice	1,48754191	1,06546537	1,0712698
MAU_142	Aedes Albopictus	Right	Maurice	1,48049787	1,1048723	1,11000266
MAU_143	Aedes Albopictus	Right	Maurice	1,45771556	1,07202828	1,07930147
MAU_144	Aedes Albopictus	Right	Maurice	1,43957721	1,0099054	1,02661794
MAU_145	Aedes Albopictus	Right	Maurice	1,51612942	1,09228675	1,11135897
MAU_146	Aedes Albopictus	Right	Maurice	1,49618086	1,07851659	1,09920961
MAU_147	Aedes Albopictus	Right	Maurice	1,43068046	1,05831369	1,0832775
MAU_148	Aedes Albopictus	Right	Maurice	1,46793253	1,0751489	1,08861749
MAU_149	Aedes Albopictus	Right	Maurice	1,43805829	1,08093501	1,08111909
MAU_150	Aedes Albopictus	Right	Maurice	1,49064889	1,05079602	1,05381829
MAU_151	Aedes Albopictus	Right	Maurice	1,41975596	1,03461714	1,05107189
MAU_152	Aedes Albopictus	Right	Maurice	1,49459091	1,09611179	1,11481332
MAU_153	Aedes Albopictus	Right	Maurice	1,48215418	1,04727798	1,06747915
MAU_154	Aedes Albopictus	Right	Maurice	1,48422377	1,07222423	1,06081535
MAU_155	Aedes Albopictus	Right	Maurice	1,44425468	1,05756504	1,08006875
MAU_156	Aedes Albopictus	Right	Maurice	1,45986155	1,05817991	1,06328106
MAU_157	Aedes Albopictus	Right	Maurice	1,45326684	1,08818126	1,08900259
MAU_158	Aedes Albopictus	Right	Maurice	1,5030172	1,07224314	1,07982513
MAU_159	Aedes Albopictus	Right	Maurice	1,45766749	1,0674859	1,09053161
MAU_160	Aedes Albopictus	Right	Maurice	1,4518477	0,97371883	0,97608067
RUN_01	Aedes Albopictus	Right	Réunion	1,4946868	1,01831573	1,02357472
RUN_02	Aedes Albopictus	Right	Réunion	1,50254584	1,03184947	1,03942861
RUN_03	Aedes Albopictus	Right	Maurice	1,44810188	1,04216924	1,04235846
RUN_04	Aedes Albopictus	Right	Réunion	1,45894787	1,05672287	1,05448218
RUN_05	Aedes Albopictus	Right	Réunion	1,46781621	1,06124989	1,06088401
RUN_06	Aedes Albopictus	Right	Réunion	1,4593995	1,07640033	1,09155441
RUN_07	Aedes Albopictus	Right	Réunion	1,45813729	1,03820808	1,06524812
RUN_08	Aedes Albopictus	Right	Réunion	1,42970864	1,05652756	1,07683093
RUN_09	Aedes Albopictus	Right	Réunion	1,51593989	1,04510564	1,06004913
RUN_10	Aedes Albopictus	Right	Réunion	1,48206319	1,06777244	1,07626275
RUN_11	Aedes Albopictus	Right	Réunion	1,42953753	1,0818946	1,0888545
RUN_12	Aedes Albopictus	Right	Réunion	1,41404655	1,09013366	1,08337023
RUN_13	Aedes Albopictus	Right	Réunion	1,39712248	1,05322666	1,05168781
RUN_14	Aedes Albopictus	Right	Réunion	1,45335366	1,08468424	1,07776896

RUN_15	Aedes Albopictus	Right	Réunion	1,49256506	1,08142374	1,09231431
RUN_16	Aedes Albopictus	Right	Réunion	1,38947399	1,10313753	1,10326629
RUN_17	Aedes Albopictus	Right	Réunion	1,50302329	1,09091214	1,0999775
RUN_18	Aedes Albopictus	Right	Réunion	1,4424296	1,06792784	1,06990528
RUN_19	Aedes Albopictus	Right	Réunion	1,47473226	1,05227866	1,05603861
RUN_20	Aedes Albopictus	Right	Réunion	1,4760481	1,07314969	1,07877051
RUN_21	Aedes Albopictus	Right	Réunion	1,43217924	1,09341383	1,07876373
RUN_22	Aedes Albopictus	Right	Réunion	1,43680656	1,08132788	1,0827048
RUN_23	Aedes Albopictus	Right	Réunion	1,47866751	1,02095065	1,02597836
RUN_24	Aedes Albopictus	Right	Réunion	1,48914549	1,08191546	1,08516121
RUN_25	Aedes Albopictus	Right	Réunion	1,49767578	1,08895786	1,09491715
RUN_26	Aedes Albopictus	Right	Réunion	1,47997059	1,07441795	1,08600985
RUN_27	Aedes Albopictus	Right	Réunion	1,49855629	1,05878017	1,07107216
RUN_28	Aedes Albopictus	Right	Réunion	1,47469395	1,08408231	1,07562005
RUN_29	Aedes Albopictus	Right	Réunion	1,48407671	1,08472205	1,08811499
RUN_30	Aedes Albopictus	Right	Réunion	1,47293393	1,01964294	1,02756723
RUN_31	Aedes Albopictus	Right	Réunion	1,46503718	1,04823612	1,05244917
RUN_32	Aedes Albopictus	Right	Réunion	1,47437117	1,04743812	1,05481347
RUN_33	Aedes Albopictus	Right	Réunion	1,52445206	1,08131988	1,08855727
RUN_34	Aedes Albopictus	Right	Réunion	1,53829757	0,9579448	0,95628683
RUN_35	Aedes Albopictus	Right	Réunion	1,4668926	1,05695862	1,07396298
RUN_36	Aedes Albopictus	Right	Réunion	1,47622628	1,08789801	1,09690026
RUN_37	Aedes Albopictus	Right	Réunion	1,44167991	1,05465977	1,07330349
RUN_38	Aedes Albopictus	Right	Réunion	1,48128557	1,07963833	1,09072314
RUN_39	Aedes Albopictus	Right	Réunion	1,49834231	1,04501583	1,05559567
RUN_40	Aedes Albopictus	Right	Réunion	1,47237784	1,05600927	1,06241395
RUN_41	Aedes Albopictus	Right	Réunion	1,49424512	1,00835076	1,01867215
RUN_42	Aedes Albopictus	Right	Réunion	1,44810702	1,02293387	1,03781666
RUN_43	Aedes Albopictus	Right	Réunion	1,49400399	1,05880704	1,07479535
RUN_44	Aedes Albopictus	Right	Réunion	1,48654626	1,06254757	1,105358
RUN_45	Aedes Albopictus	Right	Réunion	1,51369403	1,04337284	1,05264736
RUN_46	Aedes Albopictus	Right	Réunion	1,46719062	1,0994489	1,11513453
RUN_47	Aedes Albopictus	Right	Réunion	1,50547734	1,06670804	1,09565909
RUN_49	Aedes Albopictus	Right	Réunion	1,47425231	1,17217218	1,18928255
RUN_50	Aedes Albopictus	Right	Réunion	1,48623936	1,07748757	1,10344546
RUN_51	Aedes Albopictus	Right	Réunion	1,53562496	1,06629965	1,0772619
RUN_52	Aedes Albopictus	Right	Réunion	1,49184358	1,05959248	1,0763718
RUN_53	Aedes Albopictus	Right	Réunion	1,45060283	1,02292611	1,03195195
RUN_54	Aedes Albopictus	Right	Réunion	1,44075268	0,99122236	1,00272994
RUN_55	Aedes Albopictus	Right	Réunion	1,48548411	1,04163695	1,08140531
RUN_56	Aedes Albopictus	Right	Réunion	1,38623451	1,04602239	1,08707657
RUN_57	Aedes Albopictus	Right	Réunion	1,48320718	1,06259154	1,09296085
RUN_58	Aedes Albopictus	Right	Réunion	1,44010962	1,05786745	1,07322973
RUN_59	Aedes Albopictus	Right	Réunion	1,49444474	1,14694111	1,13355528
RUN_60	Aedes Albopictus	Right	Réunion	1,44422215	0,93767771	0,89767827
RUN_61	Aedes Albopictus	Right	Réunion	1,42607436	1,08744347	1,11186076
RUN_62	Aedes Albopictus	Right	Réunion	1,43098117	1,06005569	1,09072169
RUN_63	Aedes Albopictus	Right	Réunion	1,46979289	1,0719104	1,09005694
RUN_64	Aedes Albopictus	Right	Réunion	1,51271708	1,06662506	1,0864321
RUN_65	Aedes Albopictus	Right	Réunion	1,49532106	1,10246397	1,13146125

RUN_66	Aedes Albopictus	Right	Réunion	1,49364222	1,04482141	1,04107316
RUN_67	Aedes Albopictus	Right	Réunion	1,5499565	1,04716975	1,03512368
RUN_68	Aedes Albopictus	Right	Réunion	1,51801465	1,04813643	1,06884275
RUN_69	Aedes Albopictus	Right	Réunion	1,49460249	1,08137441	1,09898882
RUN_70	Aedes Albopictus	Right	Réunion	1,4685341	1,04527581	1,06171958
RUN_71	Aedes Albopictus	Right	Réunion	1,53651865	1,06312549	1,05810566
RUN_72	Aedes Albopictus	Right	Réunion	1,47287203	1,0729694	1,08714799
RUN_73	Aedes Albopictus	Right	Réunion	1,52017514	1,04799117	1,06729581
RUN_74	Aedes Albopictus	Right	Réunion	1,51319845	1,07007976	1,09175504
RUN_75	Aedes Albopictus	Right	Réunion	1,48441636	1,09805859	1,12604942
RUN_76	Aedes Albopictus	Right	Réunion	1,42245639	1,00368981	1,02816112
RUN_77	Aedes Albopictus	Right	Réunion	1,48575578	1,06171004	1,07430785
RUN_78	Aedes Albopictus	Right	Réunion	1,3571013	1,00838868	1,03691382
RUN_79	Aedes Albopictus	Right	Réunion	1,49663348	1,08022873	1,08594034
RUN_80	Aedes Albopictus	Right	Réunion	1,43355906	1,07307284	1,10052535
RUN_81	Aedes Albopictus	Right	Réunion	1,43835611	1,02800422	1,05581765
RUN_83	Aedes Albopictus	Right	Réunion	1,48253573	1,07698583	1,07960178
RUN_84	Aedes Albopictus	Right	Réunion	1,45634067	1,06064297	1,08220973
RUN_85	Aedes Albopictus	Right	Réunion	1,49664597	1,01255209	1,0365303
RUN_86	Aedes Albopictus	Right	Réunion	1,49784706	1,05159912	1,06180019
RUN_87	Aedes Albopictus	Right	Réunion	1,45414633	1,01989663	1,03355834
RUN_88	Aedes Albopictus	Right	Réunion	1,48216603	1,06185588	1,0568495
RUN_89	Aedes Albopictus	Right	Réunion	1,45407085	0,9852812	1,01655064
RUN_90	Aedes Albopictus	Right	Réunion	1,49324317	1,03519761	1,0612627
RUN_91	Aedes Albopictus	Right	Réunion	1,47857089	1,09079467	1,09179081
RUN_92	Aedes Albopictus	Right	Réunion	1,51259573	1,09897738	1,101703
RUN_102	Aedes Albopictus	Right	Réunion	1,46459665	1,08263635	1,12271557
RUN_103	Aedes Albopictus	Right	Réunion	1,43482926	1,07967127	1,10210916
RUN_105	Aedes Albopictus	Right	Réunion	1,49744889	1,0535026	1,09161291
RUN_107	Aedes Albopictus	Right	Réunion	1,47502236	1,0683098	1,09149837
RUN_108	Aedes Albopictus	Right	Réunion	1,50249379	1,08084496	1,0964952
RUN_109	Aedes Albopictus	Right	Réunion	1,43067418	1,1108477	1,14126364
RUN_110	Aedes Albopictus	Right	Réunion	1,4593862	1,06557434	1,09829578
RUN_112	Aedes Albopictus	Right	Réunion	1,49121774	1,06329427	1,08671648
RUN_121	Aedes Albopictus	Right	Réunion	1,49327349	1,0175689	1,02304844
RUN_122	Aedes Albopictus	Right	Réunion	1,52422994	1,08682801	1,10021997
RUN_123	Aedes Albopictus	Right	Réunion	1,43941254	1,03839277	1,03928933
RUN_124	Aedes Albopictus	Right	Réunion	1,53983685	1,08795811	1,11921436
RUN_125	Aedes Albopictus	Right	Réunion	1,49830435	0,9883733	0,99548757
RUN_127	Aedes Albopictus	Right	Réunion	1,49894229	1,09088508	1,0847917
RUN_128	Aedes Albopictus	Right	Réunion	1,48906652	1,07506951	1,08389579
RUN_129	Aedes Albopictus	Right	Réunion	1,41359904	1,06668182	1,10609666
RUN_130	Aedes Albopictus	Right	Réunion	1,48986307	1,06717288	1,08185832
RUN_131	Aedes Albopictus	Right	Réunion	1,45605633	1,07641188	1,08597049
RUN_132	Aedes Albopictus	Right	Réunion	1,48342958	1,05083769	1,08149259
RUN_133	Aedes Albopictus	Right	Réunion	1,47112201	1,05842102	1,07775431
RUN_134	Aedes Albopictus	Right	Réunion	1,54233362	1,04517632	1,05352246
RUN_136	Aedes Albopictus	Right	Réunion	1,44414802	1,02347559	1,04250611
RUN_137	Aedes Albopictus	Right	Réunion	1,50813937	1,08213285	1,09750487
RUN_139	Aedes Albopictus	Right	Réunion	1,50186889	1,05729465	1,06587926

RUN_140	Aedes Albopictus	Right	Réunion	1,48050786	0,96045828	0,97119087
RUN_141	Aedes Albopictus	Right	Réunion	1,47119364	1,07756456	1,07261297
RUN_142	Aedes Albopictus	Right	Réunion	1,49126981	1,12321118	1,1356978
RUN_143	Aedes Albopictus	Right	Réunion	1,44906392	1,09324578	1,0953762
RUN_144	Aedes Albopictus	Right	Réunion	1,42089908	0,9377171	0,96118539
RUN_145	Aedes Albopictus	Right	Réunion	1,485377	0,99485484	1,04103029
RUN_146	Aedes Albopictus	Right	Réunion	1,48434048	1,03335868	1,0534665
RUN_147	Aedes Albopictus	Right	Réunion	1,46483002	1,05883003	1,0653453
RUN_148	Aedes Albopictus	Right	Réunion	1,46240575	1,04332886	1,05810479
RUN_150	Aedes Albopictus	Right	Réunion	1,4760826	1,0337178	1,06123747
RUN_151	Aedes Albopictus	Right	Réunion	1,47891372	1,07285748	1,08046658
RUN_153	Aedes Albopictus	Right	Réunion	1,50533054	1,08468006	1,08541192
RUN_154	Aedes Albopictus	Right	Réunion	1,43480454	1,01999651	1,04611456
RUN_155	Aedes Albopictus	Right	Réunion	1,49941301	1,06231736	1,07574635
RUN_156	Aedes Albopictus	Right	Réunion	1,43268702	1,04593184	1,07609929
RUN_158	Aedes Albopictus	Right	Réunion	1,51499293	1,10681915	1,11832451
RUN_159	Aedes Albopictus	Right	Réunion	1,3563352	1,05962426	1,07478073
RUN_160	Aedes Albopictus	Right	Réunion	1,48068936	1,04309442	1,05992389
SEY_01	Aedes Albopictus	Right	Seychelles	1,47136795	1,05639379	1,07635498
SEY_02	Aedes Albopictus	Right	Seychelles	1,47968674	1,05023185	1,04975589
SEY_03	Aedes Albopictus	Right	Seychelles	1,43715461	1,04733505	1,04796362
SEY_05	Aedes Albopictus	Right	Seychelles	1,44312324	1,06312504	1,07109643
SEY_06	Aedes Albopictus	Right	Seychelles	1,47486975	1,03946231	1,04834748
SEY_07	Aedes Albopictus	Right	Seychelles	1,4442207	1,01378926	1,04382572
SEY_08	Aedes Albopictus	Right	Seychelles	1,44285866	1,06499669	1,06682379
SEY_09	Aedes Albopictus	Right	Seychelles	1,50181545	1,07187435	1,07297333
SEY_10	Aedes Albopictus	Right	Seychelles	1,47111099	0,95544119	0,97504177
SEY_11	Aedes Albopictus	Right	Seychelles	1,45678662	1,03894659	1,04612824
SEY_12	Aedes Albopictus	Right	Seychelles	1,45763233	1,09090139	1,08517865
SEY_13	Aedes Albopictus	Right	Seychelles	1,48522449	1,06794834	1,07565642
SEY_14	Aedes Albopictus	Right	Seychelles	1,47657798	1,0989098	1,1117982
SEY_15	Aedes Albopictus	Right	Seychelles	1,46925159	1,09385512	1,08580442
SEY_17	Aedes Albopictus	Right	Seychelles	1,42439186	1,02519679	1,04370433
SEY_18	Aedes Albopictus	Right	Seychelles	1,47796421	1,0773816	1,07502556
SEY_19	Aedes Albopictus	Right	Seychelles	1,45073288	1,08009081	1,07981858
SEY_20	Aedes Albopictus	Right	Seychelles	1,47939513	1,03010864	1,04303595
SEY_21	Aedes Albopictus	Right	Seychelles	1,43481511	1,04927766	1,05287409
SEY_23	Aedes Albopictus	Right	Seychelles	1,42848942	1,06298566	1,06821414
SEY_24	Aedes Albopictus	Right	Seychelles	1,4542069	1,08741222	1,09597279
SEY_25	Aedes Albopictus	Right	Seychelles	1,46071807	1,0802561	1,08319287
SEY_26	Aedes Albopictus	Right	Seychelles	1,53034948	1,08460131	1,08698107
SEY_27	Aedes Albopictus	Right	Seychelles	1,46959208	1,01743937	1,02562944
SEY_28	Aedes Albopictus	Right	Seychelles	1,45251394	1,06785498	1,07461299
SEY_29	Aedes Albopictus	Right	Seychelles	1,45614886	1,04836769	1,05482578
SEY_30	Aedes Albopictus	Right	Seychelles	1,4797902	1,08700359	1,0856533
SEY_31	Aedes Albopictus	Right	Seychelles	1,48840999	1,08211347	1,08595214
SEY_32	Aedes Albopictus	Right	Seychelles	1,49314067	1,10911744	1,10805492
SEY_33	Aedes Albopictus	Right	Seychelles	1,49487612	1,09886004	1,09332597
SEY_34	Aedes Albopictus	Right	Seychelles	1,44782564	1,13257774	1,17385475
SEY_36	Aedes Albopictus	Right	Seychelles	1,45262297	1,05897209	1,06365154

SEY_37	Aedes Albopictus	Right	Seychelles	1,46570834	1,06053839	1,06485049
SEY_38	Aedes Albopictus	Right	Seychelles	1,39207626	1,06246823	1,08162122
SEY_39	Aedes Albopictus	Right	Seychelles	1,46650366	1,07036101	1,08449563
SEY_40	Aedes Albopictus	Right	Seychelles	1,46908845	1,06509124	1,07671966
SEY_41	Aedes Albopictus	Right	Seychelles	1,32667609	0,83128197	0,87304196
SEY_42	Aedes Albopictus	Right	Seychelles	1,43967544	1,08661977	1,10504318
SEY_43	Aedes Albopictus	Right	Seychelles	1,42913223	1,06146535	1,06652473
SEY_44	Aedes Albopictus	Right	Seychelles	1,39971316	1,06823136	1,09977324
SEY_45	Aedes Albopictus	Right	Seychelles	1,42901252	1,03451177	1,05600943
SEY_46	Aedes Albopictus	Right	Seychelles	1,44937855	1,06846306	1,08083188
SEY_47	Aedes Albopictus	Right	Seychelles	1,43943551	1,05942587	1,06733517
SEY_48	Aedes Albopictus	Right	Seychelles	1,48221365	1,09066743	1,08748409
SEY_49	Aedes Albopictus	Right	Seychelles	1,4677943	1,0765519	1,09071803
SEY_50	Aedes Albopictus	Right	Seychelles	1,50551285	1,11873902	1,10566933
SEY_51	Aedes Albopictus	Right	Seychelles	1,47034239	1,09883856	1,09978443
SEY_52	Aedes Albopictus	Right	Seychelles	1,4346424	1,08719068	1,09131154
SEY_53	Aedes Albopictus	Right	Seychelles	1,46035048	1,05519109	1,07615508
SEY_54	Aedes Albopictus	Right	Seychelles	1,4315258	1,04493076	1,05396355
SEY_55	Aedes Albopictus	Right	Seychelles	1,45917531	1,06778419	1,08349465
SEY_56	Aedes Albopictus	Right	Seychelles	1,43957286	1,09555218	1,0999067
SEY_57	Aedes Albopictus	Right	Seychelles	1,40788687	1,05700474	1,06780939
SEY_58	Aedes Albopictus	Right	Seychelles	1,42750605	1,02345713	1,04191785
SEY_59	Aedes Albopictus	Right	Seychelles	1,4735212	1,0956006	1,09330287
SEY_60	Aedes Albopictus	Right	Seychelles	1,45374242	1,06510229	1,07386774
SEY_61	Aedes Albopictus	Right	Seychelles	1,53312446	1,11824103	1,1321332
SEY_62	Aedes Albopictus	Right	Seychelles	1,46484743	1,08239645	1,08964245
SEY_63	Aedes Albopictus	Right	Seychelles	1,46796513	1,09374741	1,09423722
SEY_64	Aedes Albopictus	Right	Seychelles	1,49056113	1,07035954	1,09619994
SEY_65	Aedes Albopictus	Right	Seychelles	1,48615224	1,06875186	1,09360954
SEY_66	Aedes Albopictus	Right	Seychelles	1,43732314	1,04476162	1,05946696
SEY_67	Aedes Albopictus	Right	Seychelles	1,45354942	1,07360855	1,09435692
SEY_68	Aedes Albopictus	Right	Seychelles	1,43835864	0,93773574	0,93795238
SEY_69	Aedes Albopictus	Right	Seychelles	1,42696264	0,99517097	0,9934899
SEY_70	Aedes Albopictus	Right	Seychelles	1,45186858	1,07872855	1,08137582
SEY_71	Aedes Albopictus	Right	Seychelles	1,44932118	0,99514196	1,00655755
SEY_72	Aedes Albopictus	Right	Seychelles	1,45048226	1,06942071	1,09118184
SEY_73	Aedes Albopictus	Right	Seychelles	1,4453556	1,08376675	1,11706313
SEY_74	Aedes Albopictus	Right	Seychelles	1,47906063	1,0583376	1,0697306
SEY_75	Aedes Albopictus	Right	Seychelles	1,47067142	1,0321456	1,0684805
SEY_76	Aedes Albopictus	Right	Seychelles	1,44849081	1,05748566	1,06373314
SEY_77	Aedes Albopictus	Right	Seychelles	1,43827635	1,08861599	1,11106835
SEY_78	Aedes Albopictus	Right	Seychelles	1,44762767	1,06120112	1,09100993
SEY_79	Aedes Albopictus	Right	Seychelles	1,49975883	0,9885269	0,99771281
SEY_80	Aedes Albopictus	Right	Seychelles	1,4569193	1,03508326	1,06277299
SEY_81	Aedes Albopictus	Right	Seychelles	1,45801541	1,12524835	1,15042528
SEY_82	Aedes Albopictus	Right	Seychelles	1,44854539	1,13771883	1,15438004
SEY_101	Aedes Albopictus	Right	Seychelles	1,46125679	1,02224802	1,06801167
SEY_103	Aedes Albopictus	Right	Seychelles	1,4747815	1,03895987	1,06373391
SEY_104	Aedes Albopictus	Right	Seychelles	1,47775353	1,10228358	1,12530452
SEY_122	Aedes Albopictus	Right	Seychelles	1,50512259	1,06709707	1,07733214

SEY_123	Aedes Albopictus	Right	Seychelles	1,47129089	1,03823117	1,06012562
SEY_124	Aedes Albopictus	Right	Seychelles	1,48381062	1,08387514	1,08403487
SEY_125	Aedes Albopictus	Right	Seychelles	1,45898985	1,07543507	1,09542724
SEY_127	Aedes Albopictus	Right	Seychelles	1,45994302	1,10615582	1,11237342
SEY_128	Aedes Albopictus	Right	Seychelles	1,47609157	1,0734871	1,07141706
SEY_129	Aedes Albopictus	Right	Seychelles	1,46532967	1,10873505	1,11800061
SEY_130	Aedes Albopictus	Right	Seychelles	1,47508749	1,05962103	1,08066855
SEY_131	Aedes Albopictus	Right	Seychelles	1,41554293	1,0396862	1,05105341
SEY_132	Aedes Albopictus	Right	Seychelles	1,48353851	1,05997874	1,06317922
SEY_133	Aedes Albopictus	Right	Seychelles	1,49953996	1,07252537	1,07866681
SEY_134	Aedes Albopictus	Right	Seychelles	1,46667981	1,05783952	1,07757845
SEY_135	Aedes Albopictus	Right	Seychelles	1,47275701	1,10486288	1,10287294
SEY_136	Aedes Albopictus	Right	Seychelles	1,38249679	1,04154852	1,05896576
SEY_137	Aedes Albopictus	Right	Seychelles	1,41642154	1,03970517	1,03768891
SEY_138	Aedes Albopictus	Right	Seychelles	1,48999082	0,96139511	0,97766296
SEY_139	Aedes Albopictus	Right	Seychelles	1,43326911	1,07349203	1,10341007
SEY_140	Aedes Albopictus	Right	Seychelles	1,46958867	1,0648557	1,09863919
SEY_141	Aedes Albopictus	Right	Seychelles	1,43563205	1,09463037	1,13772787
SEY_142	Aedes Albopictus	Right	Seychelles	1,49418799	1,11010982	1,11772758
SEY_143	Aedes Albopictus	Right	Seychelles	1,50557231	1,07498882	1,09363645
SEY_144	Aedes Albopictus	Right	Seychelles	1,44102055	1,05416653	1,10006753
SEY_145	Aedes Albopictus	Right	Seychelles	1,44879684	1,06876701	1,06663601
SEY_146	Aedes Albopictus	Right	Seychelles	1,50028977	1,09396661	1,10851333
SEY_147	Aedes Albopictus	Right	Seychelles	1,4518604	1,03881431	1,0602943
SEY_149	Aedes Albopictus	Right	Seychelles	1,42823215	1,06408079	1,05306273
SEY_150	Aedes Albopictus	Right	Seychelles	1,45390381	1,0863897	1,10895706
SEY_151	Aedes Albopictus	Right	Seychelles	1,41908353	1,05338437	1,05704794
SEY_152	Aedes Albopictus	Right	Seychelles	1,49130519	1,0682857	1,06657173
SEY_153	Aedes Albopictus	Right	Seychelles	1,48612622	1,09621829	1,0937622
SEY_154	Aedes Albopictus	Right	Seychelles	1,40216991	1,08647863	1,08756446
SEY_155	Aedes Albopictus	Right	Seychelles	1,47674356	1,13261061	1,15883499
SEY_156	Aedes Albopictus	Right	Seychelles	1,4666931	1,06405529	1,07864163
SEY_157	Aedes Albopictus	Right	Seychelles	1,46124243	1,03897242	1,03454106
SEY_158	Aedes Albopictus	Right	Seychelles	1,47260053	1,0870809	1,09442342
SEY_159	Aedes Albopictus	Right	Seychelles	1,43406799	1,05080019	1,07315005
SEY_160	Aedes Albopictus	Right	Seychelles	1,4389603	1,07777632	1,07903807

4_to_15	5_to_15	5_to_16	6_to_14	6_to_15	6_to_16	7_to_14
0,30371422	0,52493231	0,53536007	0,40200759	0,71084279	0,71414175	0,52087252
0,32991043	0,57701985	0,59560738	0,39032607	0,72263248	0,73553502	0,47182616
0,32074214	0,53572654	0,53847503	0,3918368	0,71411675	0,71022289	0,47644262
0,31539643	0,55564031	0,56884333	0,3937215	0,72010922	0,72688706	0,50901082
0,5042305	0,67459551	0,67618339	0,52799232	0,81376478	0,80953119	0,60495602
0,3303076	0,57182653	0,56788807	0,41779508	0,73565231	0,72466935	0,49823292
0,34840718	0,54713044	0,55315023	0,40483708	0,70341212	0,70488054	0,51335865
0,3186182	0,55407815	0,54949114	0,40568797	0,7093881	0,69881132	0,48324033
0,33275094	0,54508771	0,55488168	0,39017805	0,70801368	0,71161264	0,47625447
0,29814674	0,53132134	0,54645563	0,37500791	0,70655243	0,7157828	0,45429786
0,34979826	0,54298439	0,55412454	0,40834953	0,71593085	0,72018908	0,50373317
0,33728448	0,5635936	0,57087453	0,42707737	0,75045101	0,75086821	0,52483064
0,29736143	0,52323936	0,52475549	0,31591887	0,70472058	0,6987969	0,36600099
0,36539427	0,58057101	0,58783783	0,49648377	0,7396997	0,74207699	0,59646147
0,34344157	0,56890515	0,55724307	0,43088099	0,76815065	0,74864795	0,49538219
0,35263519	0,5627099	0,56905236	0,4380017	0,75017963	0,74941096	0,53741211
0,32873048	0,55260281	0,56667792	0,41825417	0,72404904	0,73224966	0,50123665
0,32467232	0,51649359	0,52646168	0,44422885	0,70887685	0,71269429	0,54180657
0,3251699	0,55985894	0,56344416	0,39303908	0,71856729	0,71720054	0,48485372
0,31050744	0,55206828	0,55448393	0,45747424	0,7358138	0,73166111	0,5516926
0,30511153	0,50868956	0,5233985	0,40879194	0,69501795	0,69999588	0,47802363
0,34240448	0,58469984	0,58938946	0,40364316	0,73392715	0,73258626	0,50021314
0,30707622	0,53945885	0,54617794	0,3746379	0,73105864	0,73118232	0,4512457
0,34934332	0,56236372	0,56095119	0,41217787	0,72466424	0,71731732	0,52386011
0,31487472	0,52734027	0,53788612	0,42064442	0,71686828	0,72028806	0,50353342
0,33972871	0,54699619	0,55311948	0,40529982	0,71694436	0,71553359	0,50240968
0,3225624	0,51707814	0,51759669	0,38739516	0,74829568	0,74036657	0,45118003
0,31173832	0,53727324	0,54565723	0,3966646	0,6985238	0,7002532	0,48796912
0,32507113	0,54491449	0,54483401	0,40710179	0,70526606	0,69732567	0,51193585
0,3495856	0,56488846	0,55712142	0,40987162	0,74553146	0,73276082	0,47028239
0,34002083	0,54316481	0,54947421	0,48883166	0,74134604	0,74067966	0,57211972
0,33540934	0,53019135	0,54709644	0,41006612	0,74896132	0,75729582	0,47533762
0,31152446	0,53795032	0,56360614	0,46383807	0,69303997	0,71353866	0,57730486
0,30774945	0,55769498	0,55461569	0,34632343	0,71807178	0,70976033	0,40409997
0,31507297	0,52880434	0,55791223	0,42333596	0,68996251	0,71040769	0,50714123
0,30479157	0,54045328	0,54748622	0,37347894	0,71808403	0,71706852	0,44591439
0,33338735	0,52093278	0,52652718	0,39507428	0,73363452	0,73463227	0,48952539
0,28844988	0,54415647	0,55991611	0,41323586	0,70804719	0,71858753	0,48878485
0,30964237	0,5445939	0,55068035	0,4324027	0,71635821	0,71656188	0,53210399
0,32867059	0,53310093	0,54835151	0,41164906	0,69718209	0,70522861	0,50218117
0,33397876	0,54881409	0,55190358	0,37634784	0,70705853	0,70530566	0,44724391
0,34420386	0,56382149	0,56510541	0,36632353	0,7271764	0,72340809	0,4517724
0,3656632	0,58587348	0,58621376	0,39946721	0,74658052	0,74183764	0,48562474
0,32622214	0,53090349	0,54263053	0,40280115	0,719555	0,72190184	0,48768692
0,34209781	0,55153774	0,55247068	0,38711261	0,72180182	0,71502198	0,47754281
0,35626327	0,59242696	0,59921471	0,40562319	0,72945556	0,7302304	0,48496769
0,33544536	0,57452042	0,55115425	0,3726851	0,72144789	0,69146468	0,44846475
0,36810352	0,59106914	0,59529902	0,40699618	0,73585931	0,74512345	0,47141841
0,36860118	0,5292107	0,52231709	0,37887248	0,69545991	0,69768386	0,45290223

0,31067216	0,53771265	0,54080342	0,40587026	0,71569618	0,71293605	0,48691952
0,34042187	0,58517121	0,59114039	0,41792211	0,73345695	0,73326007	0,49757446
0,39950473	0,61255136	0,6186069	0,56301286	0,75085199	0,75152431	0,66134327
0,37461727	0,59043808	0,60361927	0,45065658	0,78515371	0,79212674	0,52494742
0,36682852	0,58215034	0,58261731	0,40889818	0,7402405	0,73432397	0,48394974
0,316889	0,5275247	0,5228972	0,39071061	0,70131891	0,69006056	0,531645
0,35744857	0,57814975	0,55491416	0,40564786	0,74965163	0,71812713	0,47375427
0,33156875	0,56127679	0,56875626	0,3946587	0,72514112	0,72569071	0,47655115
0,35367606	0,54297738	0,53708509	0,38766777	0,7231916	0,70842152	0,45446255
0,34355743	0,55359898	0,56576977	0,4285114	0,70673959	0,71322495	0,52164751
0,33264254	0,56356659	0,56528768	0,39709557	0,72968862	0,72649535	0,48892299
0,31556179	0,53277284	0,54385783	0,42576727	0,70746676	0,71173336	0,50806215
0,30851946	0,50514215	0,51689928	0,3994028	0,69959658	0,70252823	0,48427865
0,33413452	0,53928314	0,55051618	0,44105649	0,72417676	0,72776173	0,53058881
0,29442584	0,50836094	0,5225499	0,27197127	0,67465432	0,68195394	0,33552354
0,34735334	0,57887644	0,56146892	0,41702201	0,74791135	0,72293064	0,48350927
0,32899979	0,56584432	0,54804811	0,42827168	0,74845714	0,72237169	0,50938361
0,30529068	0,53891003	0,53279461	0,37175552	0,71398934	0,70211719	0,42581832
0,31593362	0,52792124	0,53056686	0,3855545	0,7041992	0,69957107	0,45707324
0,31609765	0,54147625	0,54972559	0,41087398	0,69303063	0,69307018	0,48574878
0,36538174	0,56977418	0,56762844	0,38518849	0,7197052	0,70949214	0,47513433
0,34813781	0,55414588	0,56543378	0,43643241	0,73097833	0,73686308	0,51335526
0,3003956	0,53227353	0,52660401	0,42075164	0,70538634	0,69264605	0,50376983
0,30364297	0,51215931	0,50924929	0,38494355	0,69716767	0,68697575	0,45080661
0,31940017	0,5150173	0,51218984	0,38789378	0,70168768	0,68974817	0,45194111
0,3663945	0,56632516	0,5689318	0,42866461	0,75602203	0,75326408	0,51757917
0,31479799	0,5254968	0,53286098	0,39700388	0,73051058	0,73078871	0,47687615
0,37628014	0,5833152	0,57517896	0,43710164	0,74286691	0,72846243	0,52582292
0,30568553	0,52103608	0,54111901	0,41078531	0,68174291	0,69558832	0,48953529
0,34747265	0,58235698	0,58514739	0,41376169	0,75811097	0,75387369	0,49279903
0,32175077	0,54020048	0,54793656	0,40118864	0,71298643	0,71256253	0,47116856
0,33272539	0,53400178	0,53376829	0,35078705	0,70842457	0,70186063	0,42657179
0,35990719	0,59136347	0,58822419	0,40508647	0,75671128	0,7469138	0,46991657
0,34555603	0,55190641	0,54183809	0,43069539	0,75826828	0,7372087	0,49731865
0,35129378	0,54169118	0,52755822	0,37236422	0,71073042	0,68831672	0,45380174
0,3605504	0,57081356	0,56417213	0,40698801	0,74217818	0,72865901	0,49591601
0,26027432	0,45453152	0,44762309	0,36774529	0,60933557	0,59349008	0,45662949
0,34131523	0,57068014	0,57918769	0,43917962	0,74365622	0,74657888	0,51381069
0,36061834	0,55760088	0,55355181	0,39960636	0,73159519	0,72143286	0,50498165
0,30419995	0,53984793	0,53191104	0,40611897	0,70637156	0,68885763	0,49655446
0,33399393	0,5472996	0,53734295	0,3642668	0,70831607	0,69233368	0,44430998
0,3670236	0,59324002	0,5652935	0,37925747	0,74911109	0,71448612	0,45522826
0,3390166	0,53282476	0,53990132	0,36409496	0,67793372	0,67788397	0,4652506
0,35830246	0,56735659	0,5480558	0,38141545	0,7318886	0,70398883	0,46611777
0,31923586	0,53063015	0,51254475	0,38212803	0,70355795	0,67643489	0,4591659
0,34657895	0,59869504	0,56993658	0,38836864	0,75171169	0,71552457	0,48021367
0,35109872	0,54274138	0,54815013	0,40783232	0,72271753	0,72249446	0,49530401
0,32839701	0,57975329	0,59150818	0,45615977	0,75428921	0,76104327	0,5220495
0,30986426	0,52888269	0,53056718	0,41167293	0,71641989	0,71198382	0,49407596
0,30408264	0,53789836	0,55406268	0,43311375	0,70189019	0,71044063	0,50538989

0,33940877	0,56953212	0,5759412	0,388874	0,74922608	0,7496262	0,4650618
0,35781753	0,6032952	0,59677083	0,4602316	0,81403232	0,79709425	0,55065097
0,35201787	0,57953246	0,57792065	0,38927839	0,75392543	0,74656221	0,44447219
0,33934581	0,56527318	0,56609756	0,41576809	0,72455357	0,71936173	0,51192252
0,30625131	0,53261838	0,52596867	0,40515318	0,70470738	0,6900169	0,50359246
0,33534926	0,56538784	0,57335557	0,47002965	0,72087496	0,72216822	0,56363409
0,35445987	0,5515823	0,56101662	0,37569185	0,69828923	0,70082187	0,49234126
0,36701413	0,54977989	0,55119808	0,42967124	0,74277841	0,73960278	0,50115008
0,33543028	0,55008244	0,5572299	0,40560178	0,73181426	0,73375999	0,52239252
0,3480105	0,55085461	0,55967464	0,40020462	0,73545439	0,73528141	0,46449223
0,3177099	0,55125859	0,54684807	0,4267341	0,72137633	0,70946996	0,49631164
0,31153945	0,52755233	0,52794327	0,40962305	0,69754573	0,691473	0,48507825
0,30076391	0,53964918	0,53384343	0,42153209	0,72150368	0,70796045	0,50444551
0,32469006	0,56065517	0,56632533	0,39806226	0,71966744	0,71783855	0,46287457
0,31973233	0,54321551	0,54105963	0,40568637	0,71503199	0,70227793	0,48745983
0,35790815	0,57300564	0,57810156	0,39029272	0,74129785	0,74052933	0,47054886
0,33280199	0,52568315	0,53428514	0,36976649	0,70956888	0,71216943	0,48850962
0,29957145	0,52606777	0,5356454	0,39302585	0,68509515	0,6879372	0,47335307
0,33803678	0,55680898	0,56421673	0,43996855	0,72764913	0,7286562	0,53135287
0,37328406	0,61407033	0,6142868	0,44502492	0,77314154	0,76657913	0,50851722
0,30731356	0,52699776	0,52734319	0,37932736	0,70503732	0,69677553	0,42950118
0,33888234	0,56242425	0,55943434	0,40532862	0,72322263	0,71328125	0,497378
0,33368143	0,57931628	0,57075611	0,45591925	0,7486328	0,73169864	0,52526355
0,31443232	0,5209493	0,52515096	0,39425855	0,70267252	0,69784976	0,48506765
0,30465344	0,53083121	0,54675693	0,4355499	0,7185237	0,72612875	0,50017249
0,34943215	0,56569755	0,57599218	0,37875378	0,73283586	0,73661191	0,44587687
0,39098215	0,62077029	0,61397346	0,44438461	0,76377297	0,75273593	0,53734387
0,31166863	0,48988727	0,48271853	0,35814761	0,66813447	0,65477444	0,45291032
0,34981784	0,56060932	0,55589837	0,38158629	0,73096978	0,71807486	0,47372494
0,33622842	0,54270218	0,56620536	0,39366703	0,71535591	0,73370381	0,46468928
0,36765947	0,54785261	0,54863361	0,43284545	0,73431189	0,72474249	0,51029975
0,33218812	0,5341492	0,54161065	0,42591227	0,74402171	0,74502553	0,49288901
0,3337762	0,54482136	0,5556824	0,44051865	0,71844357	0,72463928	0,5103352
0,34765401	0,52583584	0,53854312	0,39483615	0,71397264	0,71694022	0,47145952
0,33217868	0,53689647	0,52757867	0,41573212	0,70878595	0,69307699	0,51360913
0,39905311	0,63203462	0,64811641	0,42776831	0,81847002	0,82847915	0,50317291
0,36105984	0,61016445	0,61742746	0,3768793	0,74948338	0,75222448	0,4671432
0,33535732	0,56918838	0,57414059	0,36262678	0,74183293	0,74184338	0,44756113
0,37414594	0,56586575	0,57574757	0,41749509	0,73677029	0,74343235	0,52853383
0,31172661	0,56617856	0,58443291	0,42954325	0,75720473	0,76799826	0,48844411
0,32407165	0,55939608	0,57513671	0,39114223	0,71121755	0,72079652	0,48458059
0,32965379	0,53363436	0,53745144	0,41628398	0,70593837	0,70147726	0,50319793
0,35625152	0,59356053	0,58482378	0,41086781	0,74837784	0,73225428	0,49103756
0,29262496	0,52785627	0,51785833	0,43333698	0,73056619	0,71366849	0,53116447
0,3524234	0,55400621	0,54880822	0,34491306	0,74006868	0,7301308	0,39079982
0,32692056	0,53926361	0,54281668	0,37628744	0,71392156	0,71194052	0,46880292
0,32941421	0,52940387	0,5375775	0,44765263	0,70899074	0,71037414	0,53939383
0,30879473	0,56801767	0,58647846	0,43723371	0,6920388	0,70631964	0,53731623
0,51256191	0,57359418	0,5918449	0,44020198	0,71959214	0,73043051	0,51815433
0,29787077	0,54466797	0,56443577	0,40949243	0,69293765	0,70782655	0,49351252

0,29204247	0,52915993	0,53099411	0,36846993	0,69152904	0,68716734	0,44234542
0,29675368	0,5150293	0,52729441	0,46797939	0,68983306	0,69686694	0,54934234
0,31146863	0,51030697	0,5149599	0,36862747	0,67430386	0,67273772	0,44494792
0,33388776	0,55539833	0,56270296	0,41884605	0,71683899	0,72010119	0,51188169
0,33481955	0,55295093	0,5648118	0,39081132	0,72939595	0,7347633	0,4781214
0,31161239	0,55202882	0,55939255	0,39959729	0,71399265	0,71659831	0,48017527
0,30286726	0,54525234	0,57313151	0,4248619	0,68232131	0,70470999	0,52378812
0,31867621	0,56829423	0,58035216	0,41722912	0,70059099	0,70785542	0,5096098
0,37778744	0,60902378	0,6192995	0,39055107	0,76405164	0,76852334	0,46190398
0,33132106	0,5169368	0,53032494	0,37464951	0,68395643	0,6898045	0,4538132
0,29099466	0,5407998	0,55383155	0,39893464	0,70975722	0,71450438	0,47035867
0,28984395	0,54101888	0,5446352	0,38623862	0,69285735	0,69052971	0,46662856
0,33412167	0,53039003	0,53563501	0,38403919	0,72357132	0,72121021	0,45713241
0,32069662	0,54093538	0,56217186	0,39171396	0,69035057	0,70595067	0,49189714
0,33675852	0,53088113	0,54085213	0,39329434	0,69205203	0,69670352	0,45769037
0,35599075	0,60586538	0,61453133	0,43158451	0,80657534	0,80729369	0,50004394
0,31947879	0,54250863	0,55097142	0,41398601	0,73408006	0,73791862	0,49728926
0,32664092	0,57489522	0,57970097	0,39129424	0,7348086	0,73477283	0,47500238
0,32303467	0,52501651	0,53516087	0,34347762	0,67879493	0,68182059	0,42800355
0,39929693	0,65240325	0,66871864	0,27988087	0,79433406	0,80560462	0,36772172
0,3220683	0,5416289	0,54083504	0,4066422	0,72193564	0,71400628	0,50064571
0,31698935	0,53173641	0,53295223	0,38028903	0,6814639	0,67829639	0,48868199
0,35939367	0,56162294	0,55803763	0,43512824	0,75136138	0,74152605	0,49978658
0,30364562	0,54425233	0,54857127	0,38871618	0,6900156	0,68873187	0,45062115
0,36084993	0,56887327	0,57119602	0,38089808	0,73963588	0,73692646	0,46873693
0,33145253	0,56123488	0,57199575	0,39327265	0,72155187	0,72512107	0,48859746
0,33384199	0,59622768	0,59792585	0,37916292	0,78233903	0,77702092	0,44857649
0,31243185	0,57876365	0,57134649	0,37695264	0,7399871	0,72710209	0,47434071
0,31706248	0,52108648	0,52755986	0,38544936	0,72557259	0,72246704	0,47382033
0,33291559	0,52660542	0,50938975	0,38067066	0,71759789	0,68746731	0,45540517
0,34203772	0,57173173	0,57317984	0,37158711	0,75161079	0,74810077	0,41893368
0,28613961	0,50224311	0,50334771	0,3962386	0,6755258	0,66846315	0,48992811
0,31145601	0,53168133	0,52273089	0,38331977	0,72606194	0,70823411	0,45740355
0,25035402	0,43169514	0,42864784	0,37284781	0,60254212	0,59178157	0,46818423
0,31453035	0,52700891	0,51646505	0,36053872	0,68720761	0,66969529	0,46364716
0,30592886	0,53711296	0,53864048	0,33112658	0,70772762	0,70341909	0,40902543
0,31826182	0,52525926	0,52482268	0,37573365	0,72217508	0,71408248	0,45053933
0,36731349	0,58765555	0,59135247	0,42517136	0,76447111	0,76256164	0,49673164
0,37449892	0,59726768	0,60396443	0,41176918	0,78359647	0,78362092	0,51158493
0,35258142	0,55772583	0,53355977	0,38423435	0,74005613	0,70873823	0,46471437
0,31629721	0,53627916	0,5151596	0,48385274	0,76272248	0,73409641	0,57511402
0,31919266	0,55627475	0,54133725	0,38089227	0,72908146	0,70795624	0,4710571
0,32615712	0,56846975	0,56461407	0,41576802	0,73031798	0,72142003	0,49063751
0,26933031	0,44574243	0,47712872	0,37212108	0,6308034	0,654461	0,45814006
0,41849512	0,64547388	0,69496371	0,42678659	0,85106203	0,89732613	0,50126671
0,28360538	0,52126421	0,51274052	0,43457903	0,70792552	0,69137356	0,53325035
0,33858657	0,57256282	0,55832066	0,42480661	0,72251293	0,7008914	0,52757864
0,33968211	0,54692572	0,54519873	0,3869555	0,70535449	0,69703555	0,4585783
0,36263617	0,5520434	0,55072875	0,35908824	0,70804441	0,6996717	0,43841401
0,28857982	0,51906339	0,50549798	0,3701615	0,68228309	0,66208106	0,44148584

0,3751781	0,58743661	0,57814545	0,36790896	0,74839996	0,74550027	0,44058402
0,37129505	0,56673027	0,56174343	0,32840005	0,74017435	0,74147856	0,41394656
0,3382815	0,55888359	0,55085024	0,35345206	0,72866008	0,71565095	0,44534959
0,33458878	0,54590722	0,5422877	0,38142476	0,70445459	0,6945519	0,46369752
0,33646459	0,57273373	0,57336027	0,3886987	0,73634225	0,72899166	0,47795023
0,31556883	0,54061562	0,55748518	0,32181777	0,70111852	0,71418189	0,39126067
0,31903217	0,56738864	0,56468321	0,39675786	0,72313374	0,71575986	0,45505714
0,33026036	0,5621302	0,5570575	0,35006332	0,74566625	0,7349419	0,41914002
0,33399713	0,56710634	0,56125307	0,37386205	0,72576421	0,71330116	0,43416595
0,31560642	0,53354981	0,52633935	0,39631318	0,70404237	0,68803546	0,47142157
0,37030655	0,62082537	0,61283212	0,4545724	0,78836896	0,7727108	0,52904508
0,31549316	0,54116945	0,54133555	0,38667642	0,73209002	0,72663946	0,47319488
0,39433219	0,5830743	0,57532758	0,5105961	0,79562256	0,77623342	0,59402921
0,30462991	0,51719007	0,52764791	0,3732838	0,70385838	0,70762361	0,45971233
0,30005672	0,54015983	0,52827764	0,4531402	0,74372077	0,72467335	0,5299368
0,3583597	0,58152105	0,56817751	0,41159213	0,74770822	0,72838883	0,51782571
0,34074115	0,52042258	0,52989711	0,36680493	0,69840558	0,70291981	0,45709962
0,34532677	0,54198903	0,53479254	0,39922336	0,71815566	0,70391141	0,50002477
0,37328387	0,59269992	0,5835564	0,39431046	0,79058373	0,77521646	0,46348823
0,31447587	0,54110639	0,5461775	0,35538491	0,72424041	0,72312289	0,4513624
0,37042378	0,59598725	0,59784511	0,41026108	0,77647612	0,77246357	0,49113625
0,32953343	0,55741653	0,57548413	0,35386687	0,69474936	0,70872229	0,45503771
0,38982938	0,63480256	0,61885626	0,42112909	0,82026666	0,79833002	0,48552418
0,34448132	0,55056508	0,541833	0,36380394	0,73932199	0,72257848	0,43878779
0,2758827	0,52535663	0,53897504	0,38803254	0,68233106	0,68972591	0,46757405
0,2913562	0,5011134	0,51780962	0,36084776	0,67587218	0,68446283	0,43074285
0,31295299	0,52644924	0,50707195	0,41113429	0,71430343	0,68490694	0,48013979
0,31843558	0,54025974	0,53245613	0,43694128	0,72286504	0,70812749	0,51113926
0,32633912	0,56749401	0,55020261	0,36804312	0,72086921	0,69506755	0,4616004
0,33458851	0,54376562	0,54206621	0,40314062	0,70952395	0,69829293	0,48651931
0,31541626	0,53690868	0,53579732	0,3521263	0,68192758	0,67548702	0,45330127
0,29044263	0,50240773	0,49303419	0,42700181	0,67825092	0,65970916	0,49324067
0,33763885	0,54096341	0,52814508	0,40689928	0,72417437	0,70141418	0,46991532
0,34368726	0,55473718	0,54794713	0,37190178	0,70870785	0,69520291	0,4499623
0,33918117	0,6072239	0,61483867	0,37469757	0,77528611	0,77751203	0,44409714
0,29912899	0,54322416	0,54548015	0,35672367	0,68560836	0,6809553	0,43322483
0,35408822	0,55759769	0,57069525	0,42085838	0,74417822	0,75007885	0,51069553
0,32171323	0,52507799	0,51844751	0,32939576	0,65700503	0,63987747	0,41671175
0,38663162	0,64165879	0,64484381	0,36870355	0,80737342	0,8060276	0,45209744
0,31534521	0,50961728	0,53127182	0,37255092	0,67956059	0,69489241	0,46726609
0,30425082	0,5223469	0,5277191	0,36712953	0,6975084	0,69631653	0,44309213
0,3475091	0,53421537	0,51318284	0,42926734	0,70529966	0,67587636	0,53933429
0,31391227	0,52679866	0,526502	0,39161467	0,70781521	0,70123635	0,46948155
0,33468845	0,56107243	0,56481715	0,41626068	0,71939204	0,71745971	0,49759141
0,35157276	0,53591336	0,52805387	0,38168133	0,72038645	0,70332519	0,4889914
0,34786712	0,55334329	0,54268028	0,39793395	0,7332788	0,71832856	0,47236924
0,35981675	0,56437961	0,5666951	0,32756485	0,71910465	0,71761623	0,40722191
0,36718981	0,5956652	0,59207697	0,41283597	0,75462853	0,7438547	0,49608096
0,29792977	0,52569523	0,52431879	0,37442321	0,70326927	0,69578074	0,44249663
0,33813333	0,54009748	0,54641206	0,37128646	0,72100505	0,72156782	0,46040185

0,41079285	0,66150541	0,6612768	0,39997625	0,83287755	0,82797154	0,45289629
0,3314008	0,53100078	0,5497643	0,38515962	0,70982991	0,72173935	0,47391737
0,29380751	0,50721211	0,50682376	0,39362803	0,69013498	0,684423	0,45119586
0,32485784	0,55352606	0,56602043	0,40171769	0,69413993	0,70034956	0,47326893
0,43559524	0,69120535	0,67813303	0,44647508	0,86742253	0,84972726	0,50670506
0,39598022	0,62491508	0,59382122	0,38800402	0,7982929	0,76117048	0,46355861
0,3567643	0,58821244	0,5803591	0,37843435	0,750138	0,73657406	0,45858278
0,34162042	0,58151364	0,58454981	0,41497433	0,75329731	0,75147928	0,47722438
0,3461165	0,57512382	0,57398207	0,40467072	0,74834573	0,74128656	0,47819764
0,36502228	0,62179481	0,61054895	0,39662627	0,76401904	0,7468147	0,4584125
0,31353036	0,53583548	0,5391905	0,38898375	0,72927253	0,72693726	0,46944147
0,30422005	0,5287368	0,53737722	0,37157334	0,70439106	0,71008688	0,45458654
0,36403954	0,61069894	0,59693295	0,41206478	0,75692964	0,73641255	0,49405063
0,33723696	0,56033758	0,55728374	0,3680912	0,7315128	0,72431927	0,43140437
0,33106795	0,57606422	0,56409741	0,43845659	0,7577706	0,73717515	0,50486147
0,30853249	0,4923694	0,49085634	0,33154239	0,64639418	0,6403823	0,41747489
0,34580501	0,55618515	0,55774688	0,51797059	0,7550174	0,74910444	0,60826744
0,35435356	0,58813048	0,58867786	0,3886654	0,73717618	0,73012105	0,46538584
0,34258167	0,56282304	0,55893365	0,39839566	0,72595161	0,71567531	0,47749739
0,33974614	0,58436902	0,59905857	0,37987195	0,72916894	0,73868108	0,45531791
0,31960618	0,58557913	0,60115726	0,41855259	0,7375112	0,74697895	0,48821646
0,31745879	0,55695818	0,56349716	0,42878516	0,7311025	0,73224811	0,50958469
0,32743195	0,57200659	0,57445637	0,39637871	0,74827984	0,74551762	0,48015282
0,35233428	0,60139245	0,5875602	0,42469446	0,78008009	0,75940197	0,50092685
0,35605736	0,56109157	0,57218635	0,43839441	0,7405853	0,74572308	0,5119359
0,32612361	0,54242192	0,55424351	0,34848584	0,69051599	0,69697167	0,43027266
0,42452355	0,67581486	0,66765374	0,40377405	0,83902135	0,82649908	0,45088942
0,32143959	0,5755007	0,58268525	0,40897351	0,74585053	0,74741417	0,48228791
0,31237579	0,53074407	0,54915395	0,40984786	0,70014319	0,71287085	0,50059555
0,3137869	0,54076665	0,54794743	0,36811138	0,69350705	0,69452391	0,46436676
0,26873706	0,50995087	0,51255438	0,29970972	0,64222198	0,6378981	0,39262909
0,31655804	0,51906264	0,53865413	0,37235472	0,67273729	0,68736795	0,43522127
0,34089305	0,60098547	0,59331597	0,43146087	0,75761684	0,74518546	0,52508784
0,32192029	0,53934097	0,55399778	0,40158432	0,70895972	0,71876302	0,48275852
0,30199708	0,53739651	0,55069015	0,41477428	0,70024347	0,70834171	0,50274802
0,37403648	0,56897974	0,56766984	0,39426907	0,75622671	0,74982428	0,46791863
0,32392801	0,56366999	0,57477436	0,42844868	0,73807048	0,74288255	0,5150407
0,3270766	0,56090075	0,565761	0,45015712	0,74577015	0,74636431	0,5241152
0,30489652	0,54318658	0,54928081	0,40591493	0,6978199	0,69702084	0,49515923
0,279822	0,55404927	0,5634049	0,39233828	0,70112072	0,70563718	0,46155372
0,2960212	0,5303759	0,53843445	0,34977967	0,68785342	0,69181627	0,39825507
0,34083195	0,57416399	0,57622802	0,39799081	0,77954557	0,77765299	0,48442273
0,29352163	0,58654546	0,59124878	0,44201364	0,75497096	0,75543233	0,49253665
0,38062654	0,60309411	0,61249878	0,42979777	0,75951691	0,76178923	0,50650287
0,2998335	0,5449177	0,56069886	0,37867663	0,69144881	0,70131564	0,45493285
0,31845333	0,53440348	0,54479364	0,3848477	0,69695691	0,70179233	0,46279164
0,28097564	0,51860477	0,52944693	0,36534444	0,66279271	0,66976018	0,43973835
0,2926033	0,51512979	0,52943293	0,35579641	0,66793643	0,67946834	0,45242151
0,24665784	0,48197688	0,46008692	0,39959074	0,63894992	0,60615823	0,48615431
0,33458768	0,61315915	0,61838587	0,41488966	0,73533058	0,73679191	0,48286466

0,31324772	0,56837712	0,57814328	0,4027761	0,72445814	0,72857169	0,49701487
0,32684899	0,54814883	0,54460437	0,44487194	0,70080591	0,68996016	0,56416091
0,32868538	0,54752785	0,55076229	0,41178509	0,72027115	0,71647082	0,47518861
0,30686576	0,56520152	0,56837064	0,40831651	0,72412549	0,72099448	0,48748195
0,43522028	0,70201219	0,68870622	0,51970461	0,91616685	0,89438455	0,62252836
0,31663264	0,53836914	0,53553577	0,43070821	0,70887425	0,6993005	0,50508999
0,33523038	0,55501038	0,56387494	0,43322251	0,73189191	0,73434353	0,5225782
0,32183593	0,56568816	0,54859665	0,45563131	0,73217699	0,70825969	0,52848681
0,34817867	0,58473532	0,5772793	0,43759128	0,76743566	0,75339893	0,51647167
0,32194197	0,56566477	0,56758983	0,39841938	0,7079813	0,70437307	0,49592425
0,32873079	0,58588462	0,58894593	0,42596464	0,74671348	0,74485483	0,4916648
0,30364461	0,53586251	0,55253789	0,38449879	0,70645082	0,71764164	0,45588161
0,30876664	0,54279842	0,53781956	0,37405646	0,6997841	0,69019089	0,45352378
0,280468	0,52486887	0,55088676	0,28744326	0,5910324	0,61145505	0,45743934
0,30559998	0,53994453	0,55421263	0,39082228	0,67894555	0,68809414	0,46163689
0,31081709	0,52930244	0,53947042	0,43846628	0,71067413	0,71481735	0,51083197
0,33715172	0,57583129	0,57309649	0,41020028	0,73658468	0,72669746	0,47802539
0,36200336	0,59607585	0,60131234	0,41521271	0,73322379	0,73281129	0,5193671
0,31997883	0,53621365	0,53839845	0,4179571	0,73391538	0,72743666	0,47383157
0,31863532	0,54422291	0,55051979	0,42338695	0,70318946	0,70475206	0,49489831
0,30994723	0,56387777	0,56611449	0,45113179	0,73967255	0,73592968	0,52792087
0,35836288	0,59855441	0,59560951	0,40865203	0,75082877	0,74084019	0,49099728
0,32516664	0,5347443	0,55299623	0,40496307	0,70298734	0,71469741	0,4718822
0,34477436	0,5577938	0,56032975	0,39733111	0,71120294	0,70922028	0,48812823
0,3092807	0,53144129	0,52749562	0,35657299	0,68997115	0,68095765	0,42747557
0,29676513	0,52920511	0,53749669	0,39432286	0,70651815	0,70861716	0,48839344
0,31890518	0,5270518	0,54096969	0,40864857	0,70671975	0,71448384	0,48219811
0,33913986	0,55297649	0,54783216	0,38611594	0,71574311	0,70290232	0,44044292
0,36702061	0,61500136	0,60089267	0,39923016	0,74413244	0,72565037	0,48546564
0,36074514	0,58987323	0,58603498	0,41093444	0,73663248	0,72797049	0,51190125
0,28514024	0,52730249	0,51841904	0,39672959	0,7138702	0,69990814	0,50182764
0,43362883	0,67579079	0,6890832	0,42714469	0,85859446	0,867237	0,50402625
0,38586096	0,63482005	0,64614236	0,44986443	0,82508523	0,83213812	0,5080398
0,31836516	0,5741284	0,59480089	0,40247146	0,71343054	0,72413215	0,49260043
0,38343641	0,63616384	0,64167259	0,4310002	0,81231228	0,81197892	0,51308696
0,32751767	0,5465363	0,53299952	0,41526207	0,7252206	0,7077061	0,47652305
0,29961769	0,52849855	0,51589555	0,41726729	0,71529116	0,69305836	0,48697033
0,35934493	0,57477699	0,57490303	0,38988962	0,73364907	0,72880896	0,46050375
0,38829398	0,60162502	0,57981428	0,39923945	0,76164956	0,73351872	0,46246945
0,35271863	0,57738986	0,58185907	0,42898951	0,73346516	0,73391115	0,53181209
0,31317142	0,54397685	0,53499256	0,4209683	0,70324964	0,68817493	0,51316719
0,33039957	0,5713401	0,55422248	0,40424629	0,73073786	0,70692874	0,490172
0,38952557	0,6403511	0,64270998	0,36706331	0,80352673	0,80129574	0,43193667
0,35265103	0,55759023	0,54369658	0,41981057	0,7767056	0,75688109	0,47966103
0,2840446	0,51067611	0,5019124	0,40152371	0,66282372	0,64701991	0,46110644
0,29387618	0,48341262	0,47994959	0,40539223	0,6448762	0,63530451	0,47362747
0,3822659	0,5936485	0,56440884	0,41038906	0,77451755	0,73775919	0,48987877
0,36510558	0,58877166	0,57785214	0,37346932	0,74391273	0,727286	0,44367971
0,29613289	0,52071883	0,51409308	0,39203536	0,69345908	0,67913692	0,45775477
0,31949525	0,56827886	0,57028612	0,37234859	0,73596772	0,73214945	0,44406131

0,38651904	0,60961654	0,60052336	0,39508338	0,748324	0,7340624	0,44539113
0,33080465	0,53288224	0,54676532	0,38231112	0,71178365	0,71955979	0,44948419
0,32228969	0,5398312	0,53359218	0,39405266	0,71317277	0,69878437	0,47467517
0,29151338	0,50089549	0,51454928	0,40518066	0,67642139	0,68228713	0,49872537
0,31504495	0,53487043	0,54677462	0,37442511	0,68905529	0,69642607	0,42991369
0,29537455	0,47911083	0,48481106	0,40004264	0,66942474	0,66814613	0,48840046
0,32783413	0,53112227	0,5247611	0,38737269	0,71572375	0,70239946	0,47170267
0,33773619	0,57126574	0,57169056	0,45212618	0,75465352	0,75023232	0,53512479
0,328438	0,52947424	0,54480568	0,3852624	0,71420323	0,72134983	0,45244313
0,32424254	0,53059755	0,54324704	0,373037	0,70516217	0,71002712	0,45478698
0,33762742	0,55893846	0,55443501	0,40448131	0,72552452	0,71392632	0,50138404
0,29602599	0,49602618	0,51471357	0,40890933	0,69415829	0,70459756	0,46482116
0,37477206	0,59434371	0,59237247	0,48612509	0,76391125	0,7555319	0,55334431
0,34813275	0,55246823	0,56816642	0,41623034	0,71413226	0,72350272	0,52780004
0,41097301	0,62474864	0,62271806	0,38494126	0,81361189	0,80618546	0,479405
0,32636783	0,51310976	0,50063755	0,42678051	0,72122984	0,69974746	0,53487146
0,32092872	0,54854886	0,53464964	0,39198451	0,71514085	0,69195929	0,46577828
0,30056924	0,5450079	0,51640211	0,41767112	0,68526774	0,64996908	0,49870048
0,32688577	0,50828723	0,51112604	0,37408313	0,68104641	0,67920739	0,44806199
0,32589469	0,56643815	0,55327396	0,36178373	0,71455852	0,69920474	0,42421808
0,34172361	0,55159933	0,53088701	0,42161207	0,72735463	0,69626004	0,51821485
0,3351705	0,53222965	0,54686649	0,39821583	0,71751618	0,72668958	0,48630215
0,32513738	0,49584619	0,49607089	0,35907593	0,66169924	0,65539579	0,45074181
0,36341818	0,58581885	0,57923393	0,43461695	0,74996966	0,73696738	0,52561364
0,33440133	0,52779084	0,55284265	0,42016908	0,69592138	0,71532971	0,5177711
0,33682918	0,49081947	0,48480386	0,40637517	0,70609911	0,69153824	0,48640289
0,35760632	0,585896	0,59444512	0,43376385	0,73812222	0,74186446	0,51949337
0,30235088	0,53580995	0,54983567	0,37599705	0,71709636	0,72611366	0,45160252
0,31106699	0,55071162	0,56784644	0,39826102	0,70925957	0,72068031	0,47434784
0,29625993	0,54178584	0,55091745	0,45869801	0,71554147	0,71906752	0,54811975
0,29048801	0,48428345	0,47955845	0,40210538	0,66180622	0,64602946	0,46057533
0,31393453	0,53776344	0,53636792	0,3994675	0,73243347	0,72447546	0,48572637
0,35145416	0,58161871	0,5983602	0,40475363	0,74359902	0,75439026	0,48876166
0,30157992	0,53839664	0,53963201	0,38522625	0,70163951	0,69922359	0,45117992
0,36409065	0,56734577	0,56745386	0,42533298	0,73214711	0,72173365	0,50199525
0,32255475	0,57474733	0,59025786	0,42027605	0,72375641	0,73190743	0,48425579

7_to_15	7_to_16	8_to_12	8_to_14	8_to_15	8_to_16	9_to_12
0,87276278	0,86991452	1,24564313	0,5699548	0,94089758	0,93387943	1,25518896
0,8497546	0,8577935	1,19945511	0,54735301	0,94775638	0,95234021	1,21097924
0,83811788	0,82958595	1,23214058	0,56449267	0,94973818	0,93724547	1,22820784
0,88363079	0,88561922	1,25246961	0,574954	0,97153616	0,97009915	1,25173178
0,91072835	0,90239926	1,14689372	0,60138186	0,92079415	0,90870341	1,0959784
0,85519849	0,83988295	1,23922798	0,59529965	0,98146923	0,96165135	1,23206528
0,86525157	0,86160692	1,20939234	0,56751147	0,94003292	0,93344206	1,20395921
0,82681135	0,81253989	1,17313891	0,56427699	0,94181159	0,92303645	1,17291415
0,84250272	0,84142778	1,17526045	0,53665359	0,9294249	0,9245274	1,16061201
0,82886469	0,83436621	1,17227001	0,52365348	0,92944679	0,93065878	1,18045603
0,85255765	0,85189928	1,19211592	0,57070523	0,94270303	0,9379805	1,20215089
0,8907737	0,88665513	1,22256967	0,58566622	0,97606245	0,96812915	1,22632627
0,82357339	0,81305224	1,16205655	0,43470401	0,93435234	0,91938244	1,16447466
0,86857262	0,86707163	1,22385411	0,67793244	0,96797259	0,96331632	1,21487869
0,87080031	0,84638951	1,22956445	0,56713038	0,96908732	0,94006828	1,22246637
0,8832357	0,87895413	1,20944421	0,5936127	0,96642726	0,95790496	1,22674664
0,8510545	0,8546298	1,22099595	0,58903316	0,96436676	0,96410324	1,22059454
0,8472408	0,84700146	1,19324405	0,61694746	0,94309071	0,940044	1,17814549
0,84352095	0,83928331	1,17005142	0,54854796	0,92687682	0,92014112	1,17545948
0,86344012	0,85536266	1,20906851	0,61851189	0,95006533	0,93888962	1,20653043
0,79892948	0,79939306	1,19122651	0,56130999	0,90820971	0,90406413	1,19562669
0,86837828	0,86301182	1,20644188	0,5596983	0,95084807	0,94180891	1,21225495
0,85316545	0,84997979	1,20888758	0,51822744	0,95474053	0,94765275	1,22368269
0,8812857	0,86960538	1,18961704	0,55024166	0,92596469	0,91164335	1,1917909
0,83548378	0,83479593	1,16659773	0,57589066	0,93067501	0,9262983	1,17319932
0,85308911	0,8477423	1,21282539	0,55678633	0,93925031	0,92885311	1,25525021
0,84191106	0,83085764	1,16282009	0,50874995	0,925108	0,91015327	1,18479989
0,83671327	0,83409967	1,23012751	0,57002395	0,94851816	0,94183168	1,23783741
0,84670419	0,83395529	1,20493617	0,58219151	0,9367055	0,92028741	1,19768905
0,85228248	0,83542639	1,19792554	0,54858446	0,95733108	0,93736386	1,20514371
0,84879629	0,84474825	1,22174369	0,64987671	0,94589141	0,93831433	1,23173642
0,85192172	0,85583189	1,20230254	0,54276296	0,94798953	0,94710542	1,20723549
0,84428234	0,86052444	1,22893085	0,6527745	0,94291745	0,95539584	1,22376543
0,82740817	0,81590054	1,21966415	0,47000952	0,92802356	0,9133524	1,2319086
0,82920022	0,84285852	1,21881846	0,5813782	0,93192331	0,94081717	1,21313314
0,83085961	0,82518325	1,18870606	0,51145014	0,92384253	0,91349099	1,20369201
0,86662522	0,86482266	1,19210182	0,5577781	0,96102659	0,95627599	1,19316995
0,81587133	0,82335532	1,16739004	0,54500991	0,89325352	0,89791871	1,17093049
0,85111394	0,84787734	1,23348059	0,5961967	0,93973219	0,93297362	1,23712076
0,82321701	0,82689935	1,16306274	0,58770755	0,93244652	0,93198094	1,17234919
0,81778651	0,81275604	1,08591648	0,51402808	0,91054321	0,90237976	1,07771199
0,86260712	0,85566572	1,26275914	0,5601635	1,00293734	0,99290769	1,25252962
0,88730553	0,87851944	1,23043865	0,54820841	0,9755108	0,96396406	1,24433871
0,86400143	0,86025142	1,2494885	0,567887	0,97721599	0,96856721	1,25361133
0,855441	0,84411163	1,20495088	0,55931618	0,96294781	0,94775148	1,21416488
0,85051603	0,8459603	1,17029038	0,55229936	0,94011731	0,931529	1,16537091
0,83982396	0,80616727	1,20243241	0,52571129	0,94686414	0,9096262	1,19641637
0,84654114	0,85939524	1,21090788	0,55089571	0,95424641	0,97017247	1,20973729
0,81109199	0,81801752	1,21782462	0,53576113	0,92106804	0,93270437	1,22629555

0,84210311	0,83529308	1,18709721	0,5548285	0,9387257	0,92820859	1,19351783
0,85712048	0,85202517	1,19325308	0,54806865	0,93267894	0,92361545	1,19242171
0,87539876	0,87195793	1,24547667	0,7297606	0,96463488	0,9571012	1,25328218
0,89848346	0,90174885	1,2466767	0,58568174	0,98602672	0,98568324	1,25022985
0,85794032	0,84780026	1,23383458	0,55801183	0,96014727	0,94607461	1,237532
0,8914798	0,87561787	1,18776797	0,5651664	0,94607	0,92702363	1,18281015
0,86025633	0,82370266	1,19498378	0,54952781	0,96367336	0,92274309	1,19655551
0,85755522	0,85317392	1,20578194	0,56056603	0,97139621	0,9627388	1,20806375
0,83102409	0,81108386	1,20029086	0,52887602	0,93107897	0,90669904	1,21222535
0,83796504	0,8402279	1,19008294	0,5925608	0,93113992	0,92994113	1,19622963
0,85597803	0,84973223	1,20972157	0,56130733	0,94869591	0,93980741	1,21293699
0,83508859	0,83384909	1,2162408	0,57505522	0,92932826	0,92346553	1,21493175
0,82372329	0,82166311	1,19180007	0,56275349	0,92643403	0,91999938	1,19222994
0,8542521	0,8532642	1,25867411	0,6300044	0,98064544	0,97549604	1,26075924
0,79854144	0,80156168	1,18264832	0,39353903	0,88727427	0,88619157	1,18970085
0,85336545	0,82403616	1,21927143	0,56963211	0,96711722	0,93380633	1,22138296
0,86654015	0,83583281	1,23159955	0,57638775	0,95803403	0,92327952	1,24481705
0,80134082	0,7870971	1,16862173	0,48740081	0,89152312	0,87451669	1,17588016
0,81283875	0,80411009	1,19034638	0,521517	0,9032216	0,89041919	1,20049362
0,81065201	0,80591106	1,31125082	0,57825467	0,93045828	0,9216911	1,3202672
0,86494408	0,8488963	1,25095396	0,54430782	0,96284174	0,94229573	1,26009386
0,84550219	0,84758817	1,17973645	0,56328496	0,91770751	0,91664819	1,18398487
0,82095847	0,8040899	1,19742395	0,58077614	0,91970767	0,89915173	1,19563931
0,7995553	0,78597124	1,17434554	0,51269474	0,88964679	0,87238884	1,17886837
0,80497359	0,78886483	1,15763188	0,50717524	0,88869256	0,86839429	1,16365638
0,88665377	0,8798767	1,18653746	0,57683007	0,96760743	0,95788249	1,20457235
0,85499499	0,85053734	1,19393879	0,52758545	0,92550969	0,91810502	1,21354184
0,87309986	0,85448314	1,26460003	0,59911336	0,97242293	0,95024302	1,26308017
0,80812527	0,81712589	1,22012753	0,56466563	0,91178171	0,91658438	1,21560653
0,87794951	0,86962581	1,23844187	0,57423209	0,98469343	0,97286174	1,23809401
0,82327159	0,81831488	1,21321861	0,5558368	0,93506497	0,92591247	1,21950086
0,8309755	0,8202944	1,20495638	0,48860629	0,91995283	0,90566611	1,21886603
0,86768718	0,85313842	1,16777748	0,53746186	0,9614689	0,94329578	1,17954635
0,86784533	0,84093191	1,22669012	0,58972759	0,98878417	0,95681	1,23127153
0,83193379	0,80462053	1,18956637	0,51765911	0,9206399	0,88881391	1,19911859
0,86867948	0,85053669	1,23154923	0,57515149	0,97196979	0,94958262	1,23548708
0,73503675	0,71384112	1,18171303	0,54418589	0,84422635	0,8186156	1,18033866
0,85356887	0,85279103	1,21194887	0,61503309	0,97566246	0,97204315	1,2044869
0,87457576	0,86078026	1,24080351	0,59554233	0,98503863	0,96847731	1,24680217
0,83715482	0,81530114	1,19437587	0,55241148	0,91903488	0,8932412	1,19806558
0,83440184	0,8144174	1,19235553	0,51713794	0,93543808	0,91187277	1,18707804
0,87139001	0,83188506	1,24658907	0,55036872	0,99909774	0,95471582	1,2337627
0,83052145	0,82466745	1,18594769	0,53808694	0,92879632	0,91850468	1,18887286
0,86096778	0,82819189	1,19846064	0,54222259	0,96177901	0,92526225	1,21061408
0,83873978	0,80533243	1,2177572	0,53783315	0,94913091	0,91099374	1,22631827
0,88300692	0,84157521	1,23305654	0,55423499	0,98040385	0,93453674	1,22181088
0,84903248	0,845116	1,16786687	0,57000194	0,94656889	0,9394972	1,17291463
0,86091497	0,86268222	1,24341452	0,60004003	0,96190501	0,95995554	1,24458066
0,83856656	0,83034522	1,20432109	0,57435945	0,9463545	0,93443406	1,1893009
0,81540861	0,8181397	1,16721466	0,57916786	0,91327579	0,91152215	1,17215377

0,86590829	0,86283393	1,2100244	0,53999933	0,96909435	0,96246709	1,22856244
0,96747694	0,9435654	1,3448831	0,61312893	1,06410792	1,03494335	1,34690729
0,85333517	0,84259143	1,22065745	0,52334426	0,96336846	0,94938858	1,24338384
0,8585605	0,84942503	1,20233331	0,5780495	0,94292386	0,93116298	1,19661545
0,85114748	0,83134932	1,22680021	0,57993574	0,95564989	0,93161078	1,22389119
0,85304858	0,84929632	1,22627955	0,64928423	0,96426436	0,95607102	1,21689907
0,85728067	0,85558276	1,20950698	0,56756268	0,96039831	0,9540599	1,20131558
0,85092833	0,84565368	1,23841102	0,58507963	0,9689439	0,96090143	1,256198
0,89839039	0,89643366	1,24146664	0,58608341	0,9900722	0,98460493	1,24942011
0,83629696	0,83202707	1,15945254	0,51643962	0,91824174	0,90908758	1,18941912
0,82886561	0,81207811	1,18697142	0,5812167	0,94274921	0,9210013	1,19733137
0,82548326	0,81490056	1,17741995	0,53887251	0,90784192	0,8939035	1,17498049
0,84241865	0,82360052	1,25889753	0,59290393	0,95215534	0,92945964	1,2553274
0,82491905	0,81824007	1,16715692	0,51265058	0,90077792	0,8897263	1,16564491
0,83181666	0,81452789	1,20659386	0,58214595	0,96267527	0,93847929	1,20728619
0,87808011	0,87182193	1,22924223	0,52347941	0,95905487	0,94859097	1,23928996
0,87606213	0,87412528	1,16712279	0,52011316	0,92714714	0,92210282	1,17620978
0,80665886	0,80536477	1,22613409	0,55095888	0,91331939	0,90769938	1,23977584
0,87059921	0,86638871	1,22043351	0,61090675	0,9788856	0,97063032	1,21750531
0,88530388	0,87328331	1,20555486	0,5632239	0,96715749	0,95114769	1,20263229
0,80130561	0,78796852	1,16344653	0,50496565	0,91132084	0,89270749	1,16450589
0,86422725	0,84929377	1,1961035	0,55330469	0,95131423	0,93161151	1,21719843
0,8545663	0,83322763	1,21167252	0,59968384	0,95611771	0,93058171	1,23767806
0,8414257	0,83085236	1,20429345	0,54634671	0,93069088	0,91503412	1,19979448
0,81724816	0,82040739	1,25058072	0,60474733	0,94821982	0,94688979	1,25100496
0,84982129	0,848761	1,19065037	0,52354956	0,95208696	0,94756586	1,19996934
0,88935589	0,87524751	1,23252016	0,58153089	0,95177093	0,93527352	1,22205287
0,80847297	0,79195862	1,20338767	0,51677597	0,90092052	0,88136031	1,21293896
0,86664485	0,84954881	1,20333562	0,52390778	0,94405581	0,92291091	1,21050262
0,83213389	0,84666647	1,21419867	0,54033844	0,93606253	0,94705286	1,21554108
0,8514506	0,83589416	1,23921938	0,60904125	0,97687493	0,95599389	1,23952355
0,85017787	0,84774815	1,20613125	0,56743392	0,95249826	0,94672148	1,20387986
0,8222719	0,82575547	1,1964024	0,58244483	0,92139054	0,92195217	1,20583363
0,84973187	0,84618474	1,19103373	0,5339054	0,94264796	0,93431078	1,19232049
0,85559052	0,83615098	1,24848367	0,59206254	0,96133054	0,93902079	1,25518901
0,94416188	0,9474065	1,24280408	0,57313862	1,03615241	1,03503473	1,23279757
0,89468693	0,89344948	1,18526722	0,52013391	0,97203587	0,9680692	1,17538725
0,86920276	0,86624411	1,18869533	0,52068159	0,96880864	0,96295466	1,17449378
0,89018936	0,8941243	1,21799779	0,58532036	0,9646211	0,96686752	1,21161924
0,848002	0,85478064	1,22073895	0,55441472	0,93856707	0,94115081	1,22667611
0,84105577	0,84718795	1,20588318	0,55123991	0,93581104	0,93774502	1,21313189
0,83699002	0,82738363	1,20830977	0,55988097	0,92034377	0,90635537	1,21758282
0,86892045	0,84744835	1,18406922	0,54748622	0,94905642	0,92320391	1,19176557
0,86211123	0,8417304	1,17459274	0,55631079	0,91597362	0,8910223	1,18487392
0,81964414	0,80744213	1,13119051	0,45489769	0,91297861	0,89788425	1,12758234
0,84789242	0,84222859	1,21359115	0,53159759	0,93236613	0,92376365	1,2209319
0,8372137	0,8346476	1,24155765	0,60834228	0,93113939	0,92489964	1,25686371
0,82585478	0,83632875	1,19077407	0,58217088	0,88893962	0,89643392	1,19678447
0,82457543	0,83145755	1,17636128	0,57459479	0,89931689	0,90257927	1,17418363
0,81947163	0,83022838	1,24758443	0,57061909	0,92205427	0,92919288	1,2540499

0,81026327	0,80172484	1,22852884	0,51382696	0,9075675	0,89565184	1,22997279
0,79859018	0,80219875	1,24897045	0,65644956	0,92727457	0,92742775	1,24201348
0,80372918	0,79806145	1,25599586	0,51105131	0,90363654	0,89404732	1,26047457
0,85202678	0,85190864	1,20392112	0,58342855	0,94116286	0,93911338	1,20325807
0,8558221	0,85744173	1,21214798	0,54170343	0,9449884	0,94283622	1,1999196
0,83701157	0,83569005	1,21695773	0,53811199	0,91689895	0,91273027	1,22695808
0,81487773	0,83405794	1,24831985	0,59695925	0,91747401	0,93225212	1,24611188
0,82800144	0,83141533	1,21986224	0,57906406	0,91996759	0,91969829	1,23027127
0,87263793	0,87328553	1,22254322	0,53941924	0,97754017	0,97404038	1,22148107
0,80725962	0,80899625	1,20894978	0,5417244	0,92732303	0,92461218	1,21864176
0,828146	0,8274142	1,22254261	0,54170238	0,93155009	0,92544679	1,23908595
0,82534919	0,817882	1,21693183	0,53329163	0,9192243	0,90790668	1,20463293
0,84303742	0,8362702	1,24795484	0,54456096	0,9612081	0,95027858	1,24993466
0,83490689	0,84678908	1,20137755	0,55561782	0,92431607	0,93286715	1,20867498
0,80202998	0,80280808	1,14940785	0,54096867	0,91677336	0,91387433	1,14774308
0,90448789	0,90199507	1,24380336	0,56925529	0,99845021	0,99222629	1,29411988
0,86076947	0,86108758	1,18979018	0,56212749	0,95388371	0,9508567	1,20069208
0,86335877	0,85941449	1,20156218	0,54437931	0,95160388	0,945354	1,20133407
0,81423146	0,81291813	1,16309713	0,4900115	0,90733921	0,90178066	1,17257058
0,92665969	0,93342216	1,21655272	0,45987759	1,03654818	1,03936899	1,20216332
0,86597655	0,85268494	1,26061734	0,58898197	0,97601245	0,95966057	1,26448841
0,84009564	0,833067	1,14657415	0,51832552	0,88876388	0,87948665	1,1626066
0,8417598	0,82901043	1,28141253	0,57934156	0,94553292	0,92913515	1,28182875
0,80355437	0,79675453	1,16506944	0,53400831	0,91315599	0,90272309	1,15924193
0,8779913	0,87142376	1,22459138	0,52693394	0,95917504	0,94996522	1,23207022
0,8602614	0,85887148	1,19291258	0,53660517	0,93357005	0,9277415	1,19970536
0,8966107	0,88661635	1,28778351	0,51376354	0,98688331	0,97318124	1,3042952
0,87766092	0,86037099	1,21444308	0,54726431	0,97321859	0,95220801	1,22547118
0,86184749	0,85265485	1,18431126	0,55233717	0,96635844	0,95237426	1,17711556
0,83980725	0,80229883	1,20485327	0,52312901	0,93146533	0,88891495	1,2172673
0,84174792	0,83504903	1,20105321	0,50045537	0,95303302	0,94331747	1,20778976
0,82017752	0,80661454	1,19633413	0,54074054	0,89447756	0,87668942	1,21155731
0,84779272	0,82449448	1,2158928	0,52497522	0,93885719	0,9119491	1,23097206
0,73564053	0,72063813	1,1312988	0,5000017	0,78985244	0,77121392	1,14719341
0,83493666	0,81284191	1,23844719	0,52793994	0,92237241	0,89664955	1,23797724
0,83167207	0,82403005	1,1762405	0,47241059	0,92331468	0,91239385	1,18730786
0,8398503	0,82724888	1,20527785	0,53229726	0,94747504	0,93100861	1,19862916
0,88460803	0,878145	1,20946215	0,56660442	0,97497522	0,96597713	1,20712362
0,9231745	0,917827	1,18839458	0,60353616	1,03692053	1,02723329	1,1769936
0,86536058	0,82982516	1,21100757	0,54511795	0,97037659	0,93169504	1,20574182
0,88018076	0,84667447	1,22433813	0,63073767	0,95369309	0,91588584	1,23132715
0,84907873	0,82474544	1,18426689	0,5556146	0,9577446	0,92956914	1,18862444
0,83893431	0,82624161	1,2086368	0,56228798	0,93054033	0,91485572	1,20341521
0,75957141	0,77844348	1,25401813	0,55162744	0,87924039	0,89381626	1,25687522
0,96587431	1,00921997	1,19959649	0,58735558	1,07693799	1,1173784	1,19351673
0,8394681	0,81795027	1,24481961	0,62847931	0,95558352	0,92992899	1,25113594
0,85887434	0,83289871	1,20981447	0,58673127	0,9421147	0,91168844	1,21056416
0,81528837	0,80217663	1,22473083	0,54575415	0,92684793	0,90957409	1,22738984
0,83345906	0,82082567	1,18932359	0,52103092	0,94585742	0,92902656	1,20278289
0,79224375	0,76742427	1,16090936	0,49216536	0,86640514	0,83772072	1,16936549

0,85324533	0,85374049	1,16800169	0,51273341	0,94596856	0,94986965	1,18472966
0,87069642	0,87617349	1,18207529	0,46434662	0,94782698	0,95799118	1,19881069
0,87488344	0,85758861	1,23010253	0,49418614	0,94601097	0,92597963	1,23974536
0,8257792	0,81245337	1,18791293	0,52973543	0,91975362	0,90285383	1,20593918
0,86653873	0,85349261	1,19075094	0,53749443	0,9493638	0,93151586	1,19160337
0,81599622	0,82614068	1,18924046	0,48236181	0,93373062	0,94118024	1,1803173
0,80797001	0,79843711	1,18534685	0,53911607	0,91575309	0,90366897	1,20189365
0,84724635	0,83362356	1,2087458	0,49757842	0,95351164	0,93601539	1,20533557
0,82601975	0,8099732	1,21158273	0,49364476	0,90868671	0,8898689	1,2327663
0,81228888	0,7914338	1,14282948	0,53669893	0,9068478	0,87986238	1,12184748
0,90806814	0,88645147	1,23957953	0,58039897	0,98432804	0,95831672	1,25934122
0,85230131	0,84403412	1,19633183	0,52491355	0,92804256	0,9167153	1,19815476
0,90442128	0,88055175	1,25717807	0,65061528	0,98291304	0,95405725	1,25196765
0,83354108	0,83275009	1,15864532	0,51521333	0,91100854	0,90677509	1,14978014
0,84872473	0,82605709	1,15620959	0,5864653	0,92444852	0,89864122	1,17222382
0,89255344	0,86843466	1,19814094	0,59158428	0,98603154	0,95847421	1,2019532
0,82382917	0,82544415	1,18926835	0,52590273	0,91578613	0,91436108	1,18983979
0,86262192	0,84328473	1,18855455	0,54486597	0,9245985	0,90245542	1,19219236
0,89945957	0,8801871	1,19209446	0,5218465	0,9826955	0,96000161	1,19964435
0,86125477	0,85579956	1,21457606	0,53858963	0,97064526	0,96151128	1,22430884
0,88601616	0,87865445	1,15662066	0,56520913	0,98333889	0,971933	1,1583754
0,83964451	0,84944518	1,20735396	0,5289427	0,93561636	0,9418834	1,21772837
0,91442121	0,88895599	1,23719453	0,5542572	1,00413419	0,97532387	1,25131555
0,8532988	0,8323257	1,2104371	0,51767073	0,9644807	0,93802239	1,21196303
0,80835517	0,81118925	1,20221519	0,54060435	0,9075186	0,90671967	1,20306888
0,79536957	0,7981602	1,20670377	0,51515652	0,90583153	0,90443188	1,21579327
0,8216732	0,78651073	1,18630651	0,54929542	0,91531045	0,87540524	1,19905239
0,82857397	0,8099761	1,18558836	0,57931349	0,91741732	0,89555945	1,1770759
0,8642611	0,83193489	1,18126359	0,5389117	0,96596318	0,92899501	1,19277222
0,84675058	0,82814926	1,21500551	0,5632972	0,95484077	0,9302057	1,22643058
0,82806946	0,81766636	1,17484316	0,5032654	0,90464622	0,89040416	1,18011994
0,77736158	0,7525545	1,17783804	0,55976921	0,86764428	0,8370397	1,17288819
0,82716981	0,79794454	1,20560488	0,55131117	0,93337544	0,89898684	1,21129588
0,83115735	0,81308975	1,1906088	0,54149716	0,95096312	0,92873408	1,18534822
0,87806266	0,87730533	1,22258476	0,50952423	0,9657369	0,96204373	1,22697164
0,82100666	0,81087563	1,1880888	0,51130718	0,93211689	0,91739365	1,19281047
0,87300996	0,87436027	1,24924995	0,5781333	0,96673613	0,96358379	1,24942272
0,80647861	0,78292525	1,16976419	0,4926597	0,92356433	0,89299692	1,17710047
0,92314937	0,91940497	1,19111526	0,51389912	1,00515046	0,99908705	1,21302677
0,82254026	0,8338293	1,22738614	0,52255817	0,90613189	0,91360769	1,23263696
0,81268788	0,80786928	1,2712989	0,51812048	0,91345782	0,90516821	1,27026776
0,85207767	0,81729805	1,22934954	0,62104989	0,95278614	0,91437101	1,23068709
0,84086286	0,82963977	1,20768988	0,49915077	0,89543671	0,88119057	1,2234134
0,83597749	0,83060937	1,19958329	0,58262378	0,94410242	0,93562296	1,20786004
0,87505826	0,85192972	1,19000233	0,55909615	0,97009345	0,94217388	1,19650968
0,84870281	0,831029	1,18409115	0,53449482	0,93082148	0,91138315	1,19516675
0,85372225	0,84948539	1,20513787	0,48628231	0,95548746	0,94956378	1,21099855
0,87890607	0,86350941	1,19830198	0,55130331	0,95687475	0,93782203	1,18793174
0,81793123	0,80652009	1,17841248	0,50140582	0,90438152	0,88970385	1,17687712
0,85505422	0,85217306	1,21460802	0,53225967	0,95335139	0,94732968	1,21667371

0,92202926	0,91480364	1,21814935	0,51224577	1,01370177	1,00341561	1,22719072
0,83130242	0,83956467	1,2157738	0,5479938	0,92986152	0,93389481	1,22601308
0,77893103	0,77012863	1,18061311	0,51751021	0,8720461	0,85956643	1,19799693
0,79999453	0,80119211	1,16076962	0,52707636	0,87322743	0,87039123	1,16488355
0,95150057	0,93174799	1,17602974	0,57303129	1,03778888	1,01574251	1,16898698
0,91414902	0,87275411	1,253042	0,54157958	1,01633536	0,97135339	1,25284872
0,86449133	0,84795565	1,19204175	0,51650037	0,95281062	0,93211855	1,19029623
0,84757586	0,84310222	1,19832879	0,54400551	0,93702521	0,92995717	1,20149445
0,86230923	0,85077546	1,18521727	0,52677425	0,93502648	0,91955793	1,18156857
0,86151839	0,83978861	1,19005998	0,52740956	0,95623695	0,92987634	1,19004704
0,84288486	0,8374267	1,20478856	0,51866623	0,91514542	0,90637656	1,2278203
0,82565102	0,82929779	1,23079255	0,53186786	0,92987225	0,93123765	1,24736241
0,87687379	0,85210698	1,20890237	0,57402219	0,97913279	0,95098461	1,1990977
0,82307276	0,81388119	1,22731139	0,51230178	0,93032726	0,91840718	1,22899445
0,85692443	0,83139327	1,19063444	0,56766026	0,94001643	0,91051572	1,19240849
0,78495	0,77572438	1,16889704	0,49952947	0,8894769	0,87825096	1,16127179
0,86764872	0,85818393	1,25587508	0,6718063	0,95216355	0,93832576	1,26465896
0,85242307	0,84158837	1,21954542	0,54572158	0,96155227	0,94660827	1,21815718
0,84816077	0,83319127	1,19040846	0,5405181	0,93485378	0,91629409	1,18768461
0,84724742	0,85218707	1,21181134	0,52890902	0,9418176	0,94347313	1,20839092
0,84064574	0,84572989	1,19836684	0,55464929	0,92812937	0,92932771	1,18873936
0,84787696	0,84468544	1,17724864	0,55758491	0,9230811	0,9150756	1,16737751
0,87246548	0,86631352	1,20462435	0,50491565	0,92211202	0,91272942	1,22232243
0,89423121	0,86862946	1,28667996	0,57653492	0,99084274	0,96160805	1,28460107
0,85096209	0,85229606	1,19536972	0,58055221	0,94115928	0,93947876	1,19416506
0,81074123	0,81436737	1,22576387	0,49493139	0,90596886	0,90583876	1,21646613
0,9183078	0,90301164	1,19506132	0,53728332	1,03182006	1,0134479	1,19939659
0,86042411	0,85778494	1,21626396	0,55657155	0,95482507	0,94937579	1,21359634
0,8281592	0,83721215	1,1837031	0,5513009	0,90261249	0,90811095	1,20432417
0,83949056	0,83549931	1,17749742	0,51542415	0,91580307	0,90782703	1,16242474
0,75799368	0,74894218	1,14170846	0,48524855	0,86452325	0,84973202	1,14227458
0,77535828	0,78612557	1,20197858	0,5484956	0,9092791	0,91738906	1,19299173
0,88861399	0,87285844	1,2115473	0,57640812	0,96119951	0,94274715	1,2086447
0,8386322	0,84429719	1,19055009	0,53726844	0,91871025	0,92113579	1,19535667
0,83229202	0,83602795	1,16114939	0,5412877	0,89332193	0,89378256	1,16169849
0,87014322	0,86059836	1,17973493	0,53508097	0,96194216	0,94970298	1,18614427
0,8632379	0,86328277	1,17888086	0,56783911	0,93927222	0,93515622	1,18116528
0,85203252	0,84959091	1,18809202	0,58994783	0,9422598	0,93662526	1,19335869
0,81704348	0,81321372	1,18583936	0,55988079	0,90555909	0,89821535	1,18231995
0,80486644	0,80561959	1,15034831	0,51169471	0,87914482	0,87602676	1,14851731
0,78731806	0,78822126	1,169126	0,4519632	0,87426468	0,87224917	1,17396496
0,9003576	0,8958631	1,21576618	0,52300717	0,95708593	0,95049611	1,21127449
0,83588683	0,83308222	1,19994065	0,56243297	0,92903448	0,92300298	1,20194806
0,87070957	0,86895717	1,22546965	0,57290006	0,96693018	0,96003687	1,23776319
0,80968976	0,81491708	1,24069515	0,52184797	0,90297318	0,90374234	1,23290203
0,82068785	0,8212071	1,20259922	0,53698774	0,92894574	0,92449927	1,20313923
0,77790715	0,78217079	1,16349289	0,51095521	0,87540009	0,87706065	1,15900798
0,80012598	0,80970377	1,16180654	0,506081	0,88080036	0,88777821	1,16822191
0,76369451	0,7251121	1,16874744	0,54837747	0,84863062	0,80569152	1,16473951
0,83269588	0,83174672	1,18188004	0,52587505	0,89955837	0,8957858	1,20344035

0,8598013	0,85989398	1,21556164	0,53063306	0,91557576	0,91234948	1,23245757
0,85267253	0,83791591	1,21742663	0,63556701	0,94787046	0,9290416	1,19184096
0,82097508	0,81292266	1,17660327	0,5443875	0,91863363	0,90616026	1,15649241
0,84214189	0,83539164	1,20481268	0,54318798	0,92309066	0,91300146	1,19765394
1,05799949	1,03115959	1,47673326	0,71633804	1,17741321	1,14607267	1,48361586
0,81699391	0,80313429	1,21115523	0,58035818	0,91381896	0,89644612	1,20965059
0,85029067	0,84950608	1,19979034	0,5818972	0,93157002	0,92727001	1,19625168
0,82713829	0,79997993	1,21406834	0,60859813	0,92688281	0,89606932	1,21469907
0,87485927	0,85695435	1,20736139	0,57806868	0,95748142	0,93571459	1,2207958
0,83985055	0,83234771	1,23667171	0,56644167	0,93017017	0,91941484	1,24093509
0,838633	0,83386182	1,19051468	0,54749988	0,91384453	0,90612524	1,19306876
0,81085505	0,81829412	1,17669255	0,52124205	0,89697084	0,90101515	1,18844777
0,81068786	0,79854515	1,15135357	0,52914727	0,90721108	0,89266589	1,15239772
0,80543939	0,82296003	1,19262024	0,53130162	0,90033627	0,91481082	1,20873111
0,78646654	0,79159819	1,18839162	0,53387574	0,88445146	0,88557223	1,19455689
0,81635153	0,81664087	1,19108877	0,56529187	0,89368692	0,89037643	1,20203591
0,83945519	0,8250181	1,2020955	0,54987458	0,93592166	0,9171228	1,2126351
0,87511965	0,86982393	1,21423985	0,58434685	0,95675676	0,94837569	1,21118665
0,81676572	0,80691286	1,21407058	0,55360844	0,92718419	0,91210991	1,22323743
0,80560422	0,80393912	1,14760184	0,54772741	0,87767893	0,87326982	1,15278367
0,84902531	0,84114681	1,21835374	0,58935557	0,92752332	0,91677565	1,20107338
0,8639652	0,84981717	1,18460096	0,56942781	0,96127855	0,9435453	1,17796144
0,80618224	0,81381547	1,17743137	0,52919957	0,89125543	0,89428402	1,19268993
0,83818787	0,83299387	1,20848229	0,56624537	0,93689995	0,92910575	1,20966685
0,79434266	0,78312802	1,22951431	0,4995867	0,89971552	0,88508844	1,22597437
0,84449289	0,84067818	1,14348351	0,51769709	0,88697147	0,88052515	1,13525871
0,8109836	0,81558342	1,14243087	0,53406538	0,89012878	0,89054115	1,16238175
0,81292328	0,79412002	1,1085933	0,49249832	0,89257245	0,86853864	1,1192808
0,8598677	0,83910168	1,25023669	0,56069539	0,9587531	0,93534802	1,26422871
0,87293551	0,86078671	1,18056044	0,55762755	0,93487323	0,92043035	1,17559025
0,84827401	0,83148415	1,25155111	0,55308062	0,91810834	0,89863064	1,24393864
0,96789637	0,9730687	1,2432121	0,56304107	1,04609233	1,04822662	1,24578584
0,90328384	0,90845881	1,23147731	0,57118125	0,98912861	0,99123981	1,22791038
0,83423919	0,83911161	1,20572758	0,56112955	0,92212462	0,92158435	1,21167439
0,92557612	0,92129556	1,19192662	0,5616172	0,99408579	0,98615595	1,20520881
0,82050046	0,80021295	1,15232701	0,52012246	0,88784688	0,86501096	1,16895512
0,81238702	0,78518222	1,16049067	0,54691658	0,89577828	0,8628463	1,16410582
0,83707145	0,82968126	1,18152626	0,52582728	0,92801283	0,91771559	1,18191773
0,86213527	0,83003584	1,16656013	0,52823457	0,95090115	0,91552611	1,1776761
0,87938705	0,87656078	1,2086934	0,56658008	0,93561329	0,93035248	1,22224364
0,82639613	0,80752358	1,16497646	0,57005333	0,90625995	0,88342675	1,16352745
0,840056	0,81378188	1,16425023	0,55700602	0,92879599	0,89919438	1,15697741
0,90540342	0,90022679	1,2062947	0,49346841	0,98886971	0,98104228	1,211913
0,86260889	0,8400308	1,20689291	0,55901729	0,96065185	0,93556582	1,20153604
0,75324686	0,73317696	1,12303254	0,52491503	0,84032538	0,81614012	1,12774746
0,74768799	0,73408921	1,13862576	0,54035917	0,83863948	0,82134618	1,14345516
0,88697734	0,84627692	1,22279357	0,55658756	0,97817737	0,93345135	1,23248434
0,84182814	0,8222406	1,20595139	0,51302549	0,93261463	0,90972845	1,20540928
0,7938111	0,77527678	1,16369658	0,49531731	0,85286164	0,83075927	1,17889421
0,84216829	0,83539521	1,19885908	0,48937894	0,91313899	0,90296492	1,20627702

0,84258627	0,82406467	1,09172013	0,46761058	0,88675906	0,8652226	1,09992714
0,81551064	0,81906157	1,11999086	0,51652497	0,90159668	0,90206294	1,13244335
0,82510283	0,80699294	1,19912269	0,51033217	0,88700576	0,86420974	1,22126223
0,81092125	0,8110957	1,24935327	0,57640708	0,91520391	0,90993286	1,23933861
0,78752723	0,79146396	1,1742971	0,52153476	0,90938186	0,90999196	1,16916972
0,80620171	0,79960759	1,19150118	0,54851393	0,8919085	0,88129917	1,19854518
0,84347075	0,82629023	1,23729238	0,56670429	0,96526798	0,94472029	1,21404684
0,87412289	0,86584919	1,19275822	0,60818327	0,96756068	0,95642234	1,17978751
0,82797354	0,82993321	1,21664406	0,52648481	0,92843323	0,9262947	1,22403478
0,83264184	0,83275358	1,16106289	0,5181089	0,92005705	0,91651094	1,17056587
0,86632179	0,85049959	1,21212965	0,56210512	0,95113058	0,93196537	1,22694879
0,78973232	0,79520847	1,17414491	0,54493047	0,89837827	0,89906654	1,17808737
0,8549034	0,84302321	1,26678078	0,62931883	0,95142769	0,93573983	1,28261675
0,86486148	0,87017251	1,21393859	0,62340195	0,98432314	0,98596637	1,21024479
0,96069185	0,94900377	1,18262088	0,53605201	1,04300686	1,02803904	1,19159159
0,86050657	0,8349753	1,24262311	0,61652858	0,96502267	0,93525776	1,24477273
0,83152471	0,8023009	1,18542324	0,57111943	0,95747144	0,92464638	1,19318014
0,80029028	0,76085267	1,20373203	0,57508614	0,90115634	0,85796325	1,21451374
0,78721247	0,78312068	1,13826818	0,50027493	0,86496479	0,85819013	1,16671391
0,81811056	0,80097834	1,20425627	0,50224406	0,9182719	0,89986114	1,22537211
0,86327715	0,82521177	1,20198858	0,58516691	0,95542063	0,91119374	1,19684199
0,83796386	0,84313021	1,18066398	0,53527618	0,90317231	0,90538176	1,18250424
0,80590537	0,79543184	1,16820474	0,49729089	0,88082681	0,86686252	1,19293613
0,88818139	0,87087244	1,24730517	0,5720272	0,95999017	0,93945277	1,26480725
0,84103694	0,85533623	1,18286936	0,57425564	0,92423294	0,93412469	1,1744796
0,82122265	0,80230285	1,17605799	0,54507493	0,90173586	0,87900773	1,19321683
0,8523804	0,85286137	1,17512961	0,57698075	0,92896573	0,92628841	1,18438986
0,83278808	0,83845463	1,19387873	0,52482014	0,93034412	0,93294913	1,20635169
0,82323385	0,83097212	1,18185889	0,52729809	0,89847078	0,90296882	1,20625608
0,83406405	0,83439696	1,20625161	0,61458221	0,91962054	0,91712569	1,2180103
0,75831353	0,73721719	1,12211042	0,52805975	0,85509986	0,82872716	1,13952452
0,85282329	0,84158754	1,20332755	0,54020401	0,92746205	0,91342007	1,20962848
0,86887694	0,87589625	1,25949767	0,5704617	0,98230203	0,98477574	1,2639703
0,79943524	0,79478325	1,13976923	0,51145516	0,8808525	0,87410636	1,13936717
0,84718272	0,83059733	1,16773715	0,5649858	0,93345301	0,91175343	1,16664495
0,81731335	0,82037091	1,15173141	0,56827141	0,91950441	0,91834521	1,15308082

9_to_14	9_to_15	9_to_16	10_to_12	10_to_14	10_to_15	10_to_16
0,57033098	0,95065805	0,9409534	1,21000846	0,51482057	0,90720937	0,89295602
0,5416425	0,95946061	0,9599313	1,19245114	0,5153284	0,94196379	0,93953501
0,54657872	0,94612873	0,9300046	1,18770016	0,49849015	0,90709944	0,88782718
0,55838778	0,97069055	0,96561585	1,21140312	0,51022849	0,93141862	0,92345477
0,54063589	0,8719316	0,85567343	1,04304477	0,48282565	0,82157182	0,80198905
0,57398002	0,97515033	0,95189794	1,1991269	0,53246205	0,94427937	0,91781946
0,54496244	0,93447695	0,92441638	1,15367345	0,48623184	0,88535473	0,87258534
0,55114951	0,94184173	0,92058554	1,13675115	0,50265114	0,90733365	0,88271639
0,50392534	0,91592164	0,90682387	1,13705736	0,47306374	0,89409391	0,88230997
0,51615832	0,93894679	0,9364719	1,14031169	0,46960103	0,90066842	0,89557429
0,5667744	0,95228794	0,94421975	1,14494504	0,49680386	0,89631402	0,88326748
0,57306468	0,9791858	0,96780561	1,20276489	0,53985969	0,9560034	0,94177023
0,41674823	0,93661862	0,91796964	1,12685157	0,36781376	0,90024828	0,87788818
0,65525588	0,95852456	0,95070467	1,17171571	0,6032788	0,91617019	0,90545604
0,54908172	0,96179036	0,93014842	1,18904647	0,5061008	0,92918405	0,89430912
0,59314913	0,98269504	0,97057038	1,17817684	0,53490387	0,93422437	0,91929888
0,57332486	0,96374549	0,96004722	1,20495937	0,54909377	0,9489759	0,94239297
0,58596323	0,92769668	0,92163652	1,14637073	0,54403926	0,89694805	0,88813719
0,53974442	0,93136319	0,92220176	1,13076561	0,48622073	0,88675463	0,87537828
0,60003923	0,94747427	0,93291629	1,19227261	0,57711128	0,93412194	0,91707041
0,54952853	0,91242616	0,90410701	1,16409522	0,50882503	0,88194603	0,87011455
0,55048769	0,95620999	0,94384246	1,18780823	0,51848705	0,93222962	0,91742541
0,51542798	0,97069807	0,96044842	1,19387547	0,47724923	0,9424432	0,92996166
0,53649635	0,92816032	0,91083427	1,16264878	0,49862338	0,90012549	0,88035771
0,56811679	0,93694064	0,92932524	1,13571384	0,52250688	0,90001662	0,88976965
0,58557038	0,9816182	0,96837583	1,20336947	0,5229939	0,93075489	0,91414135
0,51590949	0,94701572	0,92876462	1,15675718	0,48182418	0,91955372	0,89923303
0,56166807	0,95701985	0,946927	1,19949519	0,51469591	0,92042937	0,90755688
0,55878374	0,92898124	0,9084429	1,17788215	0,53065534	0,90987902	0,88629772
0,53563667	0,96269736	0,93951714	1,16990226	0,49097551	0,92721901	0,9017594
0,64719335	0,95683148	0,94590155	1,19375118	0,60275614	0,92065926	0,90714098
0,52987066	0,95330881	0,94781662	1,17952744	0,4949822	0,92661671	0,91831175
0,63257344	0,9386933	0,9475267	1,1984393	0,59913309	0,91521274	0,9212028
0,46345913	0,94161501	0,92405789	1,20295727	0,42483261	0,9153968	0,8952277
0,55278364	0,92758726	0,9304441	1,17744717	0,5054824	0,89434287	0,89268432
0,50999955	0,93980877	0,92499868	1,17450118	0,47416259	0,91210724	0,89439891
0,54322851	0,96170569	0,95439249	1,15821956	0,50039934	0,92723568	0,91801936
0,53263753	0,89832839	0,89962627	1,14778355	0,50102262	0,87794665	0,87626315
0,58545307	0,94387069	0,93412948	1,20784169	0,54874535	0,91606026	0,90400886
0,58197614	0,94192249	0,93758114	1,15414133	0,55589799	0,92468032	0,91742349
0,49185009	0,90176151	0,89115504	1,05470618	0,45947066	0,87894794	0,86599969
0,53559247	0,99236846	0,98013571	1,23770433	0,51157307	0,97804572	0,963864
0,54320201	0,98892083	0,97446768	1,19245461	0,47918632	0,93830126	0,92067634
0,55434122	0,98188257	0,96924119	1,22109816	0,50866018	0,95154012	0,93442045
0,55140943	0,97207776	0,95313261	1,1830064	0,50814853	0,94201	0,91924573
0,53339788	0,93598407	0,92347798	1,13474985	0,49431235	0,9073316	0,89098881
0,5048758	0,94088389	0,90107757	1,1654005	0,46142624	0,91141037	0,86851666
0,53224558	0,94997425	0,96838071	1,1807805	0,49387935	0,91951852	0,93979746
0,52774504	0,92621045	0,94167096	1,18271285	0,47430689	0,88090023	0,90013431

0,54536945	0,94539267	0,93185836	1,17060952	0,51444382	0,92353806	0,90777743
0,5322916	0,9320799	0,91968663	1,16853919	0,49861952	0,9093998	0,89392613
0,72574549	0,97343284	0,9628007	1,2367958	0,70101784	0,95900898	0,9454974
0,57413174	0,98957131	0,98633575	1,21576346	0,52957146	0,95610738	0,95006748
0,54750279	0,9638111	0,94687914	1,21512504	0,51504898	0,94245725	0,9226148
0,54807541	0,9409699	0,91955412	1,13132506	0,48571454	0,8906237	0,86608417
0,53691829	0,96566991	0,92144605	1,14827914	0,48081614	0,91882112	0,87179765
0,54652708	0,97388711	0,96161048	1,18733347	0,51861004	0,95395782	0,93938639
0,52656624	0,94397094	0,91573894	1,17738342	0,48383531	0,9113358	0,87952516
0,58512415	0,9374274	0,93326589	1,17769331	0,55675189	0,92008826	0,91291483
0,54977137	0,9516032	0,93989558	1,17460984	0,50434869	0,91419524	0,900214
0,55835555	0,92810183	0,91855514	1,1824146	0,51519917	0,89689616	0,88374076
0,54931517	0,92697481	0,91689103	1,15860522	0,50428309	0,89510083	0,88039168
0,61904596	0,98336932	0,97519955	1,21394022	0,56040765	0,9389933	0,92677492
0,38596933	0,89488281	0,88985405	1,16319682	0,35642922	0,87052074	0,86194054
0,55538056	0,97007312	0,93319293	1,19193652	0,51809681	0,9422318	0,90284363
0,57322417	0,97151787	0,93307531	1,21294535	0,53114873	0,94112166	0,89940105
0,47698827	0,89896489	0,87944904	1,15973668	0,44902259	0,88418631	0,86229041
0,51634546	0,9132679	0,89702537	1,15900149	0,4649798	0,8730306	0,85333821
0,57008159	0,94044447	0,92773815	1,30153615	0,5399859	0,92493297	0,90808289
0,53546235	0,97216786	0,94742447	1,21646802	0,48357911	0,92986127	0,90203749
0,55394108	0,92279377	0,91893541	1,16038943	0,5201077	0,90161625	0,89448531
0,56508764	0,91860911	0,89459293	1,15539853	0,51725852	0,88022349	0,85330838
0,50264015	0,89390922	0,87400572	1,14062191	0,45352451	0,8567663	0,83392518
0,49563369	0,89573206	0,87154082	1,13138605	0,45487172	0,86545448	0,83831597
0,57819082	0,98571157	0,97279008	1,15468145	0,52092525	0,93661725	0,92155755
0,53254682	0,94535547	0,9349164	1,18153943	0,48847893	0,91518386	0,90079425
0,5820284	0,97178385	0,94650087	1,23585375	0,5442147	0,94695342	0,91859792
0,54592638	0,90839577	0,90985243	1,17974744	0,50060544	0,87527101	0,87319691
0,55729864	0,98401441	0,96883804	1,21945451	0,52978977	0,96593613	0,94827036
0,54434696	0,94132995	0,928016	1,20480298	0,51956002	0,92824691	0,91108651
0,48791447	0,93431807	0,91703261	1,17080583	0,43093305	0,88797697	0,8676324
0,53147474	0,97298915	0,95131921	1,14762087	0,48847963	0,94174771	0,9169305
0,57915725	0,99294344	0,95723457	1,18906554	0,52467051	0,95169604	0,91151663
0,51519866	0,93051439	0,89544702	1,1743353	0,48442838	0,90692822	0,86924265
0,56552389	0,97587303	0,95002733	1,1914415	0,51364895	0,93287504	0,90393613
0,52889105	0,83962489	0,81087577	1,16167677	0,50203182	0,81952704	0,78812609
0,58978501	0,96724208	0,96008507	1,16360039	0,54062691	0,92670986	0,91709572
0,58938977	0,99055665	0,97165146	1,20352751	0,53741365	0,94779475	0,92641895
0,54111	0,92406381	0,89496896	1,18209687	0,51626386	0,91020047	0,87841231
0,49564647	0,92922445	0,90277522	1,15448625	0,4514502	0,8968954	0,86745823
0,52566188	0,98644766	0,93937397	1,19734559	0,47932857	0,95137664	0,90107054
0,52425398	0,93177278	0,91707172	1,15575107	0,48186624	0,90014778	0,88139163
0,5387706	0,97441747	0,93444603	1,15839511	0,47624574	0,92388943	0,88047965
0,53162802	0,95820504	0,91684654	1,15061489	0,4449491	0,88468289	0,83959427
0,53180603	0,97012362	0,9209905	1,17917106	0,48149845	0,92976485	0,87709006
0,56148081	0,95066284	0,94105204	1,14326446	0,52257152	0,92100487	0,90886496
0,58704985	0,96322223	0,95795838	1,21648205	0,54984163	0,9363917	0,92801039
0,54513578	0,93144077	0,91669823	1,17764321	0,52426552	0,92085825	0,9036117
0,56929696	0,91844456	0,91278545	1,15653964	0,54178528	0,90440042	0,89431906

0,54187261	0,98744339	0,97773041	1,2025541	0,50567679	0,9621716	0,94966733
0,59130791	1,06639676	1,03205255	1,32510606	0,55503019	1,04660718	1,00760032
0,52771265	0,98588638	0,96903764	1,21165369	0,48650945	0,9549454	0,93583677
0,55574582	0,93732938	0,92210416	1,17360887	0,52457452	0,9156821	0,89778947
0,55916929	0,95258463	0,92478291	1,19461347	0,51974169	0,92432147	0,89354706
0,62515821	0,9548443	0,94314102	1,18147557	0,57851246	0,92062763	0,90533006
0,54397695	0,95236341	0,94209371	1,18115286	0,51786274	0,93294782	0,92044855
0,58420269	0,98660498	0,9763236	1,19777981	0,51562402	0,92964686	0,91737021
0,57928343	0,99804404	0,98995597	1,20228758	0,52092521	0,95216145	0,94107895
0,5300138	0,94798333	0,93511471	1,15300269	0,48512148	0,91217823	0,89642336
0,57549181	0,95284547	0,92726143	1,16232559	0,53322338	0,91836381	0,89038466
0,5189695	0,90608552	0,88919982	1,1463696	0,4773106	0,87969519	0,85969593
0,57497026	0,94915105	0,9225568	1,25970517	0,57136979	0,95531731	0,9254813
0,49185223	0,90002458	0,88406667	1,1469539	0,46518319	0,88297066	0,86379006
0,56422775	0,96372947	0,93408474	1,16785218	0,51727339	0,92553021	0,89252543
0,51966874	0,96910285	0,95560587	1,21269395	0,48458653	0,94350719	0,92710045
0,51486015	0,93662756	0,92822418	1,14466929	0,47775895	0,90610536	0,89552104
0,55202692	0,92773419	0,91919877	1,1981561	0,50317659	0,88799107	0,87675888
0,59226737	0,97691407	0,96531734	1,12805899	0,49143063	0,8899958	0,87467842
0,53833848	0,9633137	0,94294835	1,18822633	0,51060452	0,94936383	0,92532225
0,49108167	0,91303767	0,89078797	1,13328154	0,45300078	0,88299746	0,85827523
0,55796856	0,97218191	0,94902967	1,13827693	0,46921017	0,89425558	0,86785423
0,60834463	0,98168158	0,9526178	1,19726225	0,55795915	0,94214596	0,91011211
0,53009652	0,92618445	0,90748767	1,17844006	0,50037935	0,90577553	0,88398387
0,59099027	0,94989718	0,94464552	1,22118412	0,55217821	0,92281763	0,91366997
0,51424056	0,96114119	0,95236574	1,16979837	0,47791502	0,93176197	0,92045922
0,55614504	0,94136942	0,92219866	1,16566399	0,49310743	0,88604831	0,86504375
0,51140353	0,9114597	0,88947304	1,1924281	0,4835911	0,89293601	0,86906478
0,51401119	0,95118714	0,92641893	1,17098856	0,4651839	0,91297233	0,88497691
0,52719968	0,93724387	0,9454516	1,17657912	0,47790893	0,89936122	0,90447379
0,59336512	0,97751789	0,95191038	1,19276229	0,53696405	0,93245291	0,90252431
0,54667877	0,94940174	0,94043253	1,15963891	0,49215305	0,90571878	0,89405003
0,57368264	0,93085071	0,92857303	1,15867773	0,51494163	0,88534339	0,8802795
0,51604295	0,94441897	0,93159252	1,14180282	0,45216504	0,89581187	0,8783039
0,5812794	0,96864892	0,94366856	1,22234123	0,54008566	0,93780075	0,91080932
0,55183613	1,02627427	1,02179735	1,19941431	0,50832026	0,9940777	0,98489865
0,4919117	0,96146261	0,95451632	1,14994656	0,45639676	0,93625651	0,9269654
0,49016632	0,95429331	0,94565514	1,15711262	0,46323031	0,93756214	0,926469
0,55896921	0,95666442	0,95629327	1,18583961	0,52424591	0,93093347	0,92877003
0,54857474	0,94469191	0,94440811	1,19947944	0,51152982	0,91858849	0,91497653
0,54218609	0,94306016	0,94141206	1,18938147	0,50919191	0,92032034	0,9156684
0,55010541	0,93021162	0,91174129	1,18846077	0,51204304	0,9027688	0,88111892
0,54197379	0,95741103	0,92805989	1,17702627	0,51931059	0,94397654	0,91167726
0,55375407	0,93070124	0,90241565	1,1909692	0,55156886	0,94162139	0,91043292
0,44097534	0,90882477	0,8922119	1,11994887	0,42513517	0,90119361	0,88285516
0,5304161	0,94006959	0,92978768	1,1921796	0,49179635	0,91322956	0,89994441
0,60270729	0,9478476	0,93705694	1,21352889	0,54986279	0,90710367	0,89308866
0,57358877	0,89639229	0,90028041	1,16319993	0,53278898	0,86554064	0,86637253
0,55807175	0,89702076	0,89641872	1,14538783	0,51874936	0,8695198	0,8647467
0,56256752	0,92899974	0,93297895	1,22431528	0,52576559	0,90056902	0,902228

0,49925727	0,90993191	0,89471262	1,20040081	0,46042023	0,88243524	0,86431073
0,63767275	0,92084222	0,91821609	1,21325884	0,60064449	0,89388447	0,8885184
0,50048553	0,90905725	0,89674659	1,23607526	0,46564603	0,88718644	0,87197806
0,56940915	0,94049039	0,93633792	1,17908513	0,53627313	0,91730171	0,91113623
0,51519593	0,93276134	0,92743911	1,16309338	0,4690066	0,89721931	0,88861386
0,53068191	0,92789194	0,92028276	1,18980291	0,48532509	0,89263086	0,8824649
0,57981808	0,91731402	0,92833028	1,21064951	0,53667981	0,88500651	0,8928339
0,57732553	0,93027446	0,92716268	1,19629984	0,53591204	0,89749268	0,89156502
0,52521195	0,9771329	0,9704388	1,19198101	0,48537987	0,94973951	0,93898845
0,5344076	0,93755135	0,93112572	1,18495679	0,49070219	0,90554521	0,89580002
0,54357496	0,94918557	0,93929335	1,18881203	0,48236429	0,9017079	0,88731169
0,50557056	0,90770045	0,89300871	1,18471354	0,47435924	0,89048947	0,87192147
0,529317	0,96377713	0,94912023	1,21599991	0,4852488	0,93173455	0,91365378
0,54703597	0,93189337	0,93742449	1,16539384	0,48988017	0,89077171	0,89218964
0,52164077	0,9151118	0,90905619	1,12272123	0,48575916	0,89107027	0,88229537
0,60098385	1,0494535	1,03906529	1,23490087	0,53317135	0,99171349	0,97816132
0,55521286	0,96440342	0,95840629	1,18806862	0,53252631	0,95231086	0,9439799
0,53004633	0,95137108	0,94256607	1,17353088	0,493713	0,92446499	0,91337299
0,48049581	0,91704194	0,90751567	1,15386131	0,45134533	0,89955669	0,88676239
0,43957108	1,02259842	1,02101042	1,16349532	0,40316282	0,98473279	0,98072546
0,57444038	0,97988424	0,95968855	1,21917426	0,51662534	0,93622806	0,91213002
0,5189684	0,90522853	0,89350028	1,13169815	0,47948483	0,8756918	0,86189839
0,56587296	0,94636445	0,92688459	1,24738734	0,52408801	0,91341659	0,89147576
0,51093001	0,90785976	0,8936686	1,14613699	0,48664088	0,89649282	0,87880564
0,51804407	0,96635817	0,9544097	1,18779443	0,46406654	0,92316512	0,90849788
0,52880055	0,941323	0,93170372	1,16312214	0,48073473	0,90757641	0,89311698
0,51118988	1,00528862	0,98680813	1,25944069	0,45882393	0,96299718	0,94111718
0,54334631	0,98389789	0,95936757	1,21169912	0,52118204	0,97074972	0,94322318
0,52934284	0,95983814	0,94132058	1,14527642	0,48784895	0,92957707	0,90698013
0,52334185	0,94464303	0,89835722	1,19588266	0,49272033	0,92518115	0,87478077
0,49199813	0,95999885	0,94779965	1,16041545	0,43476178	0,91394234	0,89917437
0,54003979	0,91142035	0,88938288	1,17458069	0,4952198	0,87687657	0,85163909
0,52221219	0,95453034	0,92355679	1,18296092	0,4634368	0,90852955	0,87370771
0,50276996	0,80680375	0,78529975	1,10546074	0,45260812	0,76764789	0,74332212
0,51656892	0,92236794	0,89414687	1,20877759	0,47985713	0,89467749	0,86391234
0,46547903	0,93398139	0,91984328	1,15977574	0,42944412	0,9070936	0,89050216
0,50988858	0,94075298	0,92068775	1,18736434	0,49185163	0,93008606	0,90785685
0,54742848	0,97211396	0,96015073	1,18675192	0,5161216	0,95233377	0,93758946
0,58036927	1,02510551	1,01205565	1,14395543	0,53868435	0,99213494	0,97576433
0,52436441	0,96460117	0,92313971	1,17654567	0,48554772	0,93616703	0,89211314
0,62551986	0,9604128	0,91897532	1,22419976	0,61137026	0,95400732	0,90970406
0,54658079	0,96153575	0,93039274	1,143769	0,49229195	0,91712652	0,8828862
0,54465208	0,92609705	0,9075993	1,17768859	0,51032927	0,90260283	0,88107079
0,54090347	0,88336717	0,89422474	1,21151368	0,48536178	0,84165852	0,84782347
0,56979858	1,0699958	1,10841021	1,17176209	0,53763427	1,04758174	1,08343661
0,62713258	0,96237845	0,93461011	1,19876292	0,56261494	0,91269672	0,88023009
0,57437679	0,94354715	0,90975119	1,17746503	0,53336138	0,91210002	0,87539336
0,5337852	0,92965209	0,90861655	1,18415819	0,48210324	0,8878228	0,8634025
0,52052477	0,95768716	0,93824263	1,17077146	0,47677234	0,92468444	0,90208162
0,48204137	0,87616529	0,84286654	1,12338887	0,42693966	0,83376025	0,79615212

0,51619899	0,95996581	0,96673819	1,14661619	0,46949156	0,91989265	0,92954728
0,46502617	0,96098759	0,97483137	1,15998314	0,41765316	0,919851	0,9368234
0,48960446	0,95506905	0,93265275	1,21088361	0,4526174	0,92649755	0,90207723
0,5326476	0,93779372	0,91821229	1,16046906	0,47490989	0,89358574	0,87083302
0,52480969	0,94985414	0,92815534	1,16570636	0,48942095	0,92451566	0,89891855
0,45841848	0,92319434	0,92827693	1,14800727	0,41488516	0,89015813	0,89254836
0,53757949	0,93208366	0,91705861	1,17036629	0,49985967	0,90128276	0,88468505
0,48297694	0,94975032	0,92987675	1,17004978	0,43942922	0,91497491	0,89238122
0,49522791	0,92936262	0,90721571	1,20146584	0,45409294	0,89889445	0,87415497
0,4923154	0,88700041	0,8519156	1,06929517	0,43593305	0,83590256	0,79806504
0,58690231	1,00495608	0,97570346	1,23550058	0,55576208	0,98237818	0,95067778
0,50949241	0,92937085	0,9149539	1,17349132	0,47474384	0,90568205	0,88836681
0,63096278	0,97859828	0,94529309	1,23154103	0,6031732	0,95953354	0,92327899
0,49130483	0,90128041	0,89381971	1,12561376	0,45680527	0,87718284	0,86667474
0,58894093	0,94034901	0,91175067	1,14232133	0,54762063	0,9113668	0,87961246
0,58207917	0,9897203	0,95899353	1,15844489	0,52925179	0,94693119	0,91311025
0,51451576	0,91610419	0,91234099	1,16300466	0,48050654	0,88997318	0,88401956
0,53171074	0,92887424	0,90285852	1,15744966	0,48876682	0,89577584	0,8668528
0,51297886	0,98979625	0,96396188	1,1615811	0,46180548	0,95228527	0,92275204
0,53598059	0,98024793	0,96796425	1,19234017	0,49627852	0,94914039	0,9336118
0,55622571	0,98456092	0,97056115	1,12882519	0,51818236	0,95501753	0,93811315
0,5288573	0,94577174	0,94952525	1,20169788	0,50521174	0,93049306	0,93127346
0,55452808	1,0172134	0,98548935	1,21764273	0,51171903	0,98334622	0,94880315
0,5081711	0,96639508	0,93694704	1,18686564	0,47562058	0,94269232	0,90998206
0,52366546	0,90856613	0,90403477	1,17758938	0,48919358	0,88426571	0,87699622
0,50378058	0,91593145	0,90901788	1,17498408	0,45304418	0,87766004	0,86614487
0,54845952	0,92854814	0,88499625	1,1613796	0,50063712	0,89289293	0,8452883
0,55764251	0,90938146	0,88459246	1,15400679	0,52545344	0,88808249	0,86039646
0,53473845	0,97755466	0,93621122	1,15693737	0,49059454	0,94270364	0,89780994
0,56045595	0,96672695	0,9381438	1,17845334	0,50111747	0,92050396	0,88737181
0,49155637	0,90977939	0,89200661	1,1596828	0,4636839	0,89012983	0,87001758
0,54225567	0,86419673	0,82898878	1,15143812	0,51475719	0,8449701	0,80645022
0,54412081	0,9402862	0,90177538	1,17353229	0,49849522	0,90467136	0,86243621
0,52028545	0,94535905	0,91958698	1,15398748	0,47936272	0,9148884	0,8858502
0,49780069	0,96944266	0,96255411	1,19601826	0,45992974	0,93874635	0,92971836
0,50027082	0,93640838	0,91847239	1,14457359	0,43631738	0,88943839	0,86645526
0,56450669	0,96788849	0,96112893	1,23718716	0,54367944	0,95746726	0,94749323
0,48659423	0,93134521	0,89731495	1,15067039	0,448326	0,90641813	0,86814128
0,52026006	1,02587976	1,01730942	1,18009721	0,47836931	0,99251626	0,98171296
0,51040181	0,91171889	0,91567468	1,19494502	0,46105231	0,8761084	0,87626062
0,50145213	0,91376412	0,90194825	1,23779403	0,46121701	0,88411675	0,86920515
0,6139191	0,95375291	0,91335508	1,2194475	0,5913193	0,94304947	0,89925453
0,49225183	0,91210904	0,89398153	1,20415947	0,46463115	0,89451052	0,87417406
0,57611808	0,9516922	0,94044228	1,15664883	0,51409692	0,9012673	0,88683243
0,54885312	0,97596446	0,94365196	1,16274992	0,50547021	0,94252737	0,90664837
0,53027629	0,94235822	0,92095265	1,17130357	0,49877586	0,920049	0,89707396
0,47020517	0,95986972	0,95131656	1,17981702	0,43036247	0,92870801	0,9184361
0,52885107	0,94619269	0,92450414	1,17467129	0,50677572	0,93335345	0,90911134
0,48332452	0,90263918	0,88514572	1,1531544	0,44895481	0,8797634	0,85972952
0,51640445	0,95442359	0,94527322	1,18785939	0,47877494	0,92591526	0,91435011

0,50437959	1,02225066	1,00949172	1,21127203	0,48075143	1,0066794	0,99208773
0,54642029	0,94042532	0,94137461	1,1877299	0,49989991	0,90355819	0,90115882
0,52339792	0,89023037	0,87534208	1,14813705	0,46586982	0,84290105	0,8251951
0,52025387	0,87767731	0,87154998	1,13302705	0,4810105	0,84741149	0,83769708
0,55066987	1,02907421	1,00472248	1,14721896	0,52019894	1,00643274	0,98025463
0,5292045	1,01586936	0,96830841	1,21529921	0,48408701	0,97881545	0,9289089
0,50159301	0,95127671	0,92791908	1,16783884	0,46984126	0,93003539	0,90386209
0,53576502	0,94013642	0,93119291	1,16016416	0,48609904	0,89967123	0,8886931
0,51266376	0,93098627	0,91320066	1,15258894	0,47348568	0,90268289	0,88143007
0,51546122	0,95622729	0,92664818	1,14918113	0,46738096	0,91636132	0,88358522
0,53090132	0,93841003	0,92741929	1,18860288	0,48498092	0,90032181	0,88711951
0,53308806	0,94566903	0,94508867	1,2010982	0,47776136	0,90013168	0,89755352
0,55140248	0,96937844	0,93843458	1,16995194	0,51348958	0,94118775	0,9075583
0,49900602	0,93096923	0,91677567	1,19388836	0,45638156	0,8960047	0,88005509
0,55654542	0,94131653	0,9085181	1,17533888	0,53087612	0,92475008	0,8888665
0,4771652	0,88221535	0,86886587	1,12238317	0,42951035	0,8450448	0,82966523
0,67032077	0,96099111	0,94446489	1,22678569	0,62282721	0,92452752	0,90452526
0,52712535	0,96019733	0,94149072	1,18693769	0,48764331	0,92999642	0,90853513
0,52302338	0,93327295	0,91126011	1,15418131	0,48170382	0,90169427	0,87691127
0,51375958	0,93905952	0,93776244	1,16580938	0,4635991	0,89812285	0,89379403
0,53202583	0,91820867	0,91594605	1,1746572	0,50947009	0,90498978	0,89927215
0,53425504	0,91438834	0,90270216	1,13305211	0,49349074	0,8817996	0,86742379
0,50948743	0,94070247	0,9289445	1,19344023	0,47111315	0,91384942	0,8994602
0,55975452	0,98882538	0,95591522	1,24397874	0,51100589	0,94954863	0,91342779
0,56515673	0,93998323	0,93546515	1,14772665	0,50854592	0,89484498	0,88727197
0,47243277	0,8971667	0,89440376	1,18791736	0,43571649	0,87019799	0,86489979
0,52736862	1,03590898	1,01506978	1,16744112	0,4849385	1,0043301	0,98086007
0,53987219	0,95176576	0,94348427	1,18472748	0,50164806	0,92361515	0,9124941
0,55796838	0,92415366	0,92652758	1,15764233	0,50355433	0,87948497	0,87899967
0,48681153	0,901024	0,8899009	1,13480589	0,44779059	0,87510449	0,86007814
0,47976778	0,8626271	0,84366561	1,13159033	0,46861999	0,85129702	0,83024603
0,52521976	0,90047209	0,90543632	1,17875206	0,50467083	0,88720325	0,89006441
0,55915479	0,95898431	0,93786371	1,18736832	0,53160618	0,93927566	0,91625198
0,52612984	0,92377064	0,92322152	1,15769092	0,47985353	0,8872893	0,88431233
0,52612641	0,89396472	0,89126392	1,13447973	0,49024932	0,86780373	0,86257071
0,52219728	0,96820055	0,95281007	1,16043962	0,48845382	0,94314336	0,92580674
0,55763919	0,94208847	0,93475853	1,16129445	0,52939916	0,92358329	0,91316652
0,58374942	0,94803965	0,94021436	1,15349675	0,53815605	0,90912406	0,8997348
0,54149523	0,90218173	0,89171854	1,14363338	0,49343318	0,86475328	0,85147172
0,49797228	0,87861495	0,87241357	1,13888398	0,48187967	0,87120446	0,86224119
0,43726906	0,87918005	0,8744393	1,15093635	0,40635338	0,85707754	0,85063045
0,50577165	0,95207816	0,94346436	1,20178469	0,48889998	0,94295717	0,93256089
0,55308804	0,93168625	0,92330415	1,15252795	0,493536	0,88455661	0,87293804
0,57608638	0,97980082	0,97060389	1,17648584	0,50244263	0,92160827	0,90727575
0,5009095	0,89643185	0,89341246	1,19238651	0,45512862	0,85786038	0,85217184
0,526297	0,93034318	0,92326889	1,15937497	0,47301255	0,8889717	0,87841915
0,49002787	0,87081058	0,86993491	1,13065596	0,45455587	0,84331385	0,84076301
0,49632591	0,88637607	0,89108318	1,14496684	0,46471265	0,86353296	0,86638302
0,52900885	0,84732236	0,79991036	1,14080553	0,49818777	0,82627417	0,77607946
0,53099558	0,92083904	0,91438278	1,16809488	0,48786821	0,88631255	0,87785287

0,5318332	0,93307826	0,92652131	1,19804093	0,48947143	0,90028625	0,89102127
0,59478568	0,92320559	0,90063677	1,1719475	0,5677457	0,90486949	0,87991977
0,50854112	0,89991356	0,88349899	1,126288	0,47071154	0,87168814	0,85245821
0,51990384	0,91609911	0,90295528	1,16106343	0,47384671	0,88070584	0,86500255
0,7065301	1,18393777	1,14857834	1,44968482	0,66356679	1,15073903	1,11227088
0,56484296	0,91228648	0,89177613	1,17460171	0,52012468	0,87840756	0,85489436
0,56578262	0,92817918	0,92107725	1,16867153	0,52940387	0,90167601	0,8918518
0,59968709	0,92759086	0,89443151	1,18509806	0,56302221	0,89898004	0,86347061
0,57933089	0,97174642	0,9467899	1,19392787	0,54403827	0,94664273	0,91849976
0,55799858	0,93479838	0,92098442	1,21922869	0,52858066	0,9147465	0,89798823
0,53905243	0,91634185	0,90631127	1,16882814	0,50652728	0,89308529	0,88047461
0,52059354	0,90912876	0,91009902	1,15475501	0,480223	0,87657057	0,87499386
0,51826143	0,90800618	0,89150642	1,12220868	0,47977865	0,87845498	0,86000127
0,5328712	0,91648709	0,92769836	1,17089869	0,48730416	0,87990405	0,88817655
0,52685569	0,89136461	0,88931409	1,17678285	0,5000339	0,87571276	0,8702953
0,5635803	0,90543459	0,89928209	1,16897719	0,52201531	0,87427784	0,86531473
0,54810151	0,94649937	0,92463125	1,1821415	0,50910653	0,91692599	0,89202955
0,56788359	0,95389155	0,9419213	1,18026077	0,53069317	0,92390766	0,90941135
0,54891365	0,93841099	0,91938401	1,1974153	0,516568	0,91512793	0,89316453
0,53969605	0,88298762	0,87594535	1,11916435	0,49963762	0,85028199	0,84134369
0,56225684	0,91084056	0,89769348	1,17605263	0,52838612	0,88823909	0,87176429
0,55069792	0,95476291	0,93359145	1,16784224	0,53687031	0,94504475	0,92239729
0,53168289	0,90710355	0,90691593	1,14415877	0,47560729	0,86025145	0,85697024
0,55632216	0,93811901	0,9283113	1,19548359	0,53362535	0,92483995	0,91289534
0,47410376	0,89905656	0,88005211	1,16671773	0,41201482	0,84342401	0,82223564
0,49813407	0,87900707	0,86943506	1,11338889	0,46984062	0,85818875	0,84593535
0,54014363	0,91042057	0,90776817	1,12600513	0,49556389	0,87531836	0,86988267
0,48735327	0,90311446	0,87489849	1,08923737	0,44846238	0,87370049	0,8421457
0,56223948	0,97214641	0,94690536	1,19653998	0,4855163	0,90490989	0,87769542
0,54131023	0,92957367	0,91304532	1,15421061	0,51093722	0,90869289	0,88990622
0,53363552	0,91028673	0,88847847	1,21015792	0,49386798	0,87740017	0,85376466
0,55392465	1,04846603	1,04790732	1,20657369	0,50673402	1,00968731	1,00630814
0,55697366	0,98603619	0,98590448	1,19285577	0,51498498	0,95233786	0,94986682
0,55403885	0,92962237	0,92295109	1,15737211	0,49595919	0,87704003	0,8670346
0,56196418	1,00707385	0,99599043	1,16196657	0,50867779	0,96426813	0,94961266
0,52058034	0,90433215	0,87921742	1,14862511	0,49217241	0,88494609	0,85815506
0,54002338	0,90017566	0,86357939	1,14588845	0,51328851	0,88410962	0,84314969
0,51047754	0,92817498	0,91520387	1,15310243	0,4746764	0,90012878	0,8852755
0,52627795	0,96200683	0,9240007	1,14578634	0,48681282	0,93100392	0,89064832
0,56576133	0,94906875	0,9416848	1,16963136	0,50283486	0,89759407	0,8879117
0,55793113	0,90498539	0,87970016	1,12678487	0,51131147	0,86964447	0,84121668
0,53492436	0,92180822	0,88898245	1,12375853	0,4956158	0,88956915	0,8547882
0,48601149	0,99309442	0,9830499	1,17766347	0,44273225	0,95811816	0,94559511
0,53930765	0,95452041	0,92676157	1,18595781	0,51796787	0,93904802	0,90982317
0,51718964	0,8459676	0,81860248	1,1085687	0,49067629	0,82857953	0,79856085
0,53065519	0,84423688	0,82373202	1,11333666	0,49310708	0,81631323	0,79319072
0,55441963	0,98811522	0,94071592	1,20455912	0,51769431	0,96134233	0,91116093
0,49984099	0,93227102	0,90641183	1,18089069	0,46879255	0,9089956	0,8805549
0,4956303	0,86868891	0,84314991	1,15260559	0,462402	0,84393612	0,81591146
0,48336128	0,92132489	0,90833921	1,15852588	0,42864534	0,87575269	0,85996491

0,46323897	0,89523128	0,87125451	1,07440686	0,43026726	0,87110941	0,8445558
0,51398077	0,91296483	0,91004325	1,10281758	0,47628174	0,88325827	0,877574
0,5191723	0,91038701	0,88405351	1,18787654	0,48017063	0,87894436	0,85007817
0,55298198	0,90562103	0,89622512	1,20143029	0,50702474	0,86962599	0,85631948
0,49910376	0,90345781	0,90112041	1,15362279	0,47166126	0,88850875	0,88312044
0,54201846	0,8996267	0,88595253	1,16828377	0,50194623	0,87159792	0,85454379
0,53129447	0,94148728	0,91873204	1,1917262	0,49888966	0,91959925	0,89440088
0,58098065	0,95414194	0,94026662	1,14583465	0,53692423	0,92084764	0,90423059
0,51447292	0,93622724	0,92934351	1,1984639	0,4803087	0,9120951	0,9018741
0,51109959	0,92925212	0,92211608	1,1547523	0,48546169	0,91397741	0,90384353
0,56059072	0,96623796	0,94388239	1,19711722	0,52195948	0,93770779	0,9127858
0,53444364	0,9026619	0,89970556	1,14339834	0,48857639	0,86980237	0,86259942
0,63062003	0,96665774	0,94752502	1,23991624	0,57931321	0,92460826	0,90257063
0,60638151	0,98073412	0,97934801	1,21193198	0,59950035	0,98320694	0,97924975
0,52616022	1,0510081	1,03258682	1,15869278	0,48365116	1,01790661	0,99680322
0,60906448	0,96685255	0,93477858	1,20211891	0,55769195	0,92496721	0,88991693
0,5638092	0,96507174	0,92836637	1,16994477	0,5300459	0,94272749	0,90222741
0,5731005	0,91258854	0,86676535	1,19286645	0,54318087	0,8929173	0,84471284
0,51270699	0,89341048	0,88422215	1,12417081	0,46256472	0,85201667	0,84092
0,50889228	0,9385807	0,91898286	1,17598764	0,45301786	0,88983303	0,8692833
0,56853608	0,95022422	0,90230963	1,16676702	0,52454125	0,92198953	0,86757012
0,52576009	0,9054096	0,90459478	1,15861389	0,49504955	0,8829029	0,87919015
0,50413466	0,90599212	0,88891836	1,19992189	0,50025131	0,91486636	0,8948534
0,56984515	0,97717072	0,95337168	1,22915003	0,52390857	0,94246016	0,91619276
0,55672523	0,91661392	0,92423294	1,15523159	0,52504748	0,9002315	0,90344777
0,54856834	0,91790964	0,89209589	1,15792945	0,50629868	0,88287415	0,85462325
0,575101	0,93782674	0,93266846	1,15595624	0,54042953	0,90974007	0,90257547
0,52220069	0,94266009	0,94233236	1,17839136	0,48748019	0,91539668	0,91298699
0,53975033	0,9227693	0,92483799	1,14910615	0,47360009	0,86677997	0,86582538
0,613904	0,93141975	0,92645477	1,17866564	0,56692437	0,89303265	0,88591825
0,5286743	0,87306617	0,84243646	1,09685959	0,47664406	0,8322411	0,7980438
0,53362326	0,93356051	0,91704664	1,18502416	0,50045056	0,90997518	0,89088744
0,56262012	0,9877872	0,98733287	1,21225555	0,50253157	0,93845845	0,93467311
0,49842517	0,87986019	0,87136945	1,12119614	0,47208864	0,86211515	0,85194338
0,55170253	0,93258439	0,90715327	1,13722908	0,51293284	0,90444006	0,87492582
0,56050141	0,92141348	0,91702346	1,1114948	0,51070846	0,88193649	0,87278185

11_to_12	11_to_14	11_to_15	11_to_16	12_to_15	12_to_16	13_to_15
1,12271958	0,42350293	0,82286992	0,80465188	0,30475011	0,31811603	0,4147409
1,10965904	0,42763207	0,86154307	0,85545897	0,2518064	0,25442269	0,39148163
1,13899818	0,44676372	0,86060406	0,83863351	0,282454	0,30036574	0,40946751
1,16614127	0,46150249	0,88785021	0,87744177	0,281188	0,28880077	0,41026109
0,99377699	0,43183381	0,77474073	0,75271264	0,22690172	0,241118	0,39165714
1,15956333	0,49025742	0,90650024	0,8782165	0,25777337	0,28138636	0,40526107
1,10342511	0,43214925	0,8369575	0,82192609	0,26964451	0,28149903	0,4051146
1,06728979	0,42849284	0,839996	0,81306388	0,23133735	0,25425788	0,39879055
1,06978223	0,4019719	0,82953801	0,8148909	0,24584869	0,25494322	0,4221557
1,0701471	0,39658411	0,83317332	0,82541766	0,24292542	0,24482862	0,40305193
1,09242588	0,44147367	0,84525653	0,82978585	0,24986407	0,26304202	0,39539442
1,13247109	0,46326337	0,88707447	0,86980589	0,24714148	0,26332281	0,39464648
1,05697054	0,2963189	0,83215431	0,80722547	0,2279526	0,24983901	0,43368277
1,14047471	0,56791037	0,88623482	0,8734813	0,25637186	0,26713476	0,42759579
1,15105825	0,46410291	0,89281528	0,85536202	0,26069394	0,29580704	0,41239626
1,14459665	0,49575977	0,90132171	0,88418434	0,24411258	0,26182161	0,4226708
1,12546944	0,46564602	0,87093297	0,86198859	0,2569255	0,26375759	0,41023619
1,11294582	0,50767364	0,86434893	0,85436453	0,25051391	0,25869742	0,38782095
1,06663093	0,4175332	0,82342769	0,81018277	0,24417307	0,2568198	0,37520904
1,14260713	0,52147169	0,88634212	0,86667138	0,25920849	0,27596372	0,40281047
1,09277558	0,43175236	0,81336832	0,79735344	0,28330939	0,29554416	0,38452996
1,15139782	0,47826563	0,89665348	0,87999575	0,25605548	0,27215669	0,41795392
1,09850125	0,37469154	0,85054109	0,83471796	0,25429456	0,2640257	0,46093869
1,11373683	0,44491189	0,85343885	0,83119538	0,26381632	0,28258909	0,41082441
1,07834517	0,459526	0,84417324	0,83101537	0,23627988	0,24775233	0,39792778
1,14300172	0,45705148	0,87216441	0,85276826	0,27370327	0,29042095	0,39591126
1,100089	0,42035152	0,86509768	0,84124901	0,23789518	0,25891294	0,39935574
1,1061874	0,41604423	0,83047597	0,81433633	0,28161137	0,29207098	0,39804821
1,11616288	0,464349	0,84970244	0,82330171	0,26873939	0,29323849	0,38164112
1,12269899	0,43970425	0,88053292	0,85337733	0,24271885	0,26983578	0,41589095
1,11707318	0,52234183	0,8468398	0,83067003	0,27586466	0,28665419	0,39634908
1,11714816	0,4271492	0,86649037	0,85448313	0,25436178	0,26309011	0,38753761
1,13747246	0,53226394	0,8577071	0,8602458	0,28601701	0,27739222	0,36048137
1,1585047	0,37802484	0,87355877	0,85169257	0,29173568	0,30798449	0,44116834
1,1263727	0,44778665	0,84641617	0,84076553	0,28690843	0,28569588	0,38256625
1,07441511	0,36975321	0,81579118	0,79351302	0,26488161	0,28090343	0,400773
1,09034867	0,42727594	0,86072535	0,84936522	0,23148025	0,24105067	0,47070348
1,11291523	0,46321523	0,84585645	0,84203016	0,2742714	0,27159292	0,3737282
1,14678247	0,48376449	0,85715635	0,84302648	0,29377085	0,30389675	0,39470353
1,10033184	0,49624242	0,87309805	0,86219308	0,23068937	0,23846039	0,40204798
0,99798114	0,39834034	0,82295981	0,80810674	0,17597288	0,19059356	0,39904829
1,16450968	0,43302437	0,90616746	0,89003363	0,26017026	0,27450441	0,41996334
1,1316363	0,41586658	0,87877477	0,85967554	0,25543977	0,27196329	0,46119294
1,13494897	0,41666177	0,86911588	0,8476905	0,27227533	0,28727473	0,40583281
1,12553068	0,44441191	0,88663179	0,86042636	0,24218391	0,26537326	0,37344633
1,08862793	0,44551845	0,86335458	0,84429758	0,23018885	0,24438015	0,4205881
1,08668162	0,37868727	0,83553431	0,79014578	0,25567326	0,29695119	0,42395181
1,13107339	0,43850966	0,86915165	0,89118356	0,2619687	0,24135663	0,40517294
1,12356019	0,41183372	0,82156513	0,84395833	0,30206566	0,28514163	0,38640942

1,11214991	0,45077411	0,86721141	0,84894829	0,24843385	0,26321205	0,40885157
1,10452492	0,42866662	0,84771087	0,82902039	0,26061698	0,27555529	0,40519089
1,18740126	0,6464572	0,91253024	0,89636339	0,28090243	0,2913253	0,4057763
1,13969717	0,44720195	0,88232833	0,87317759	0,26078468	0,26653835	0,44629238
1,14897522	0,4439983	0,87821604	0,85582024	0,27383086	0,29316983	0,43945471
1,09072379	0,44144825	0,85165122	0,82510577	0,24188286	0,26561804	0,3853501
1,08677656	0,41638034	0,85935307	0,81008868	0,23132034	0,27672135	0,42711588
1,13371554	0,45872649	0,90265243	0,88453859	0,23445451	0,24928934	0,3967667
1,12162951	0,42571589	0,85863471	0,82373549	0,26924637	0,29801727	0,372683
1,1265258	0,50003117	0,87098461	0,86103597	0,25900885	0,26552468	0,37097782
1,1100027	0,43726601	0,85114519	0,83532802	0,26140324	0,27468044	0,40431107
1,14191821	0,46916185	0,85841599	0,84229835	0,28702836	0,29977357	0,39359007
1,09647558	0,43860359	0,83509489	0,81741009	0,26545194	0,27920206	0,3893367
1,14666702	0,488103	0,8750984	0,85956715	0,27804006	0,28728998	0,41319982
1,09585302	0,29376561	0,80574959	0,79455995	0,29541342	0,30137832	0,36931607
1,14075538	0,46268108	0,89351984	0,85174231	0,2521585	0,28918051	0,39067281
1,16946352	0,48340497	0,8993418	0,85549289	0,27362268	0,31397842	0,36398738
1,10044605	0,38401671	0,82702974	0,8031523	0,27717018	0,29750021	0,37812429
1,12388178	0,4261711	0,83959224	0,81755641	0,28733926	0,30639402	0,39165573
1,23462617	0,4698811	0,86097956	0,84170639	0,38079254	0,39346666	0,38416086
1,17900661	0,44276788	0,89400191	0,86399397	0,2882446	0,31508282	0,43209204
1,11144196	0,46860692	0,85428054	0,84566155	0,26206411	0,26590576	0,39011713
1,08376398	0,44233462	0,8111611	0,78180161	0,27771803	0,30215381	0,37023276
1,08628296	0,39466562	0,80454401	0,77931047	0,28499883	0,30701272	0,37892727
1,0750541	0,39472225	0,81176415	0,78209825	0,2689492	0,29313169	0,39369077
1,10273569	0,46331337	0,8865499	0,86883159	0,2190567	0,2339379	0,40685563
1,11703149	0,42011812	0,8528742	0,8358715	0,26845861	0,28116027	0,39192796
1,18162192	0,48619678	0,8951012	0,86481652	0,29218963	0,31726326	0,43187536
1,12592099	0,44356299	0,82407146	0,81967136	0,30840604	0,30655075	0,35519529
1,17002822	0,47398374	0,91810288	0,89753651	0,2541104	0,27277272	0,40938288
1,12741778	0,43918265	0,85285175	0,83315556	0,27838259	0,29428455	0,42323782
1,11854583	0,3755888	0,83852431	0,81534288	0,28500897	0,3033401	0,44532468
1,08617635	0,42030137	0,88214809	0,85390867	0,20658913	0,23261609	0,45114736
1,12292789	0,45455361	0,88715619	0,84414533	0,23833209	0,27913143	0,39003891
1,11101101	0,41893192	0,8455306	0,80548298	0,26895404	0,30552803	0,4398884
1,11909183	0,43813821	0,8621751	0,8307771	0,25974528	0,28843243	0,41350214
1,0903913	0,42686294	0,74819171	0,71443508	0,34233791	0,37697984	0,32014169
1,09378396	0,46653476	0,85784301	0,84620567	0,237248	0,24808584	0,41086577
1,13769774	0,46752961	0,88336959	0,85983869	0,25624606	0,27789759	0,42089759
1,11250019	0,44077641	0,8442424	0,80939864	0,2755649	0,3036884	0,37378368
1,1126566	0,40483498	0,8563466	0,82451079	0,25791657	0,28829416	0,3943599
1,12687228	0,40558498	0,88296749	0,83021538	0,24751744	0,29666662	0,444115
1,1045345	0,42782685	0,85093327	0,82925275	0,257323	0,27545348	0,4203434
1,11057481	0,42437426	0,87815376	0,8322975	0,23668234	0,27827904	0,42596714
1,09220871	0,38281507	0,82958298	0,78148068	0,26862658	0,31109608	0,40064255
1,09277322	0,39324658	0,84597352	0,79075701	0,25279728	0,30216097	0,44681631
1,08379617	0,45919609	0,86213718	0,8482197	0,22237822	0,23638368	0,41894077
1,16719137	0,49525887	0,88963862	0,87797225	0,28159493	0,28922573	0,38885061
1,08090383	0,42180668	0,82661261	0,80646885	0,25805966	0,27446324	0,40716399
1,08492418	0,46313697	0,83625402	0,82121731	0,25398191	0,26380116	0,38051019

1,13513593	0,43208754	0,89657377	0,88105466	0,24116942	0,25427589	0,4585236
1,25743193	0,48214448	0,98140317	0,93925493	0,28089743	0,31819441	0,46896585
1,14008659	0,40988617	0,88493038	0,86373534	0,25757023	0,27636054	0,43694659
1,12628621	0,47433694	0,86990439	0,85025846	0,2595728	0,27603081	0,41629905
1,13034596	0,44846339	0,86283957	0,82847465	0,27142436	0,30187593	0,39652655
1,11924462	0,5100852	0,86041146	0,84212455	0,26218191	0,27725774	0,37192522
1,14013907	0,47262102	0,89337493	0,87835614	0,24921986	0,26222672	0,41075911
1,12383481	0,43738511	0,85891525	0,84457824	0,26975536	0,28042942	0,4079848
1,14968936	0,46261293	0,90226339	0,8881075	0,25150683	0,26162686	0,3988731
1,08229921	0,40795669	0,84340941	0,82383914	0,24148217	0,2590191	0,41412043
1,10187162	0,46496293	0,86016823	0,82812391	0,24454136	0,27393735	0,40633267
1,10011576	0,42552591	0,83601234	0,81382234	0,26957868	0,28667505	0,3783868
1,21146547	0,52000677	0,90946547	0,87706213	0,30675954	0,33444373	0,39157022
1,09076044	0,40515599	0,82924591	0,80698563	0,26639335	0,28378975	0,37442037
1,09404205	0,43929631	0,85368025	0,81750914	0,24400642	0,27692019	0,42380615
1,14790965	0,4162756	0,88042613	0,86150815	0,27031477	0,28650907	0,44747073
1,08445772	0,41466207	0,8478476	0,83473536	0,24000716	0,24973719	0,41527621
1,11967059	0,42108197	0,81312858	0,79874001	0,31282324	0,32142644	0,3719256
1,08605656	0,44447103	0,85073197	0,83278386	0,24160461	0,25343538	0,39786446
1,15249612	0,46720517	0,91531374	0,88766131	0,23932607	0,26540369	0,41513585
1,04629145	0,36050866	0,79978482	0,77060996	0,25212711	0,27572384	0,39650836
1,07280663	0,39874337	0,83108153	0,80144382	0,24507656	0,27138132	0,41333128
1,14137902	0,49792149	0,88755618	0,85359832	0,2560268	0,2878605	0,36480887
1,10010734	0,41750691	0,82965284	0,80450608	0,27371763	0,29570215	0,3911985
1,15463071	0,48206227	0,85958812	0,84727278	0,30244844	0,30756885	0,36470492
1,07471203	0,3803583	0,83852519	0,82429473	0,23895751	0,2505896	0,42366851
1,08921523	0,41257038	0,81217627	0,78892584	0,28092419	0,30067443	0,45616368
1,14012489	0,42817648	0,84446786	0,81843296	0,30248073	0,32356363	0,35532953
1,12771422	0,41934384	0,8710006	0,84124876	0,25948069	0,28652404	0,41291564
1,12512175	0,42182556	0,8499388	0,85227624	0,27836463	0,27288128	0,3944693
1,14036725	0,48089698	0,88198854	0,84919011	0,2623979	0,29143988	0,40438376
1,11198295	0,43959024	0,8594977	0,8455413	0,25447883	0,26652836	0,42183047
1,10721522	0,45900912	0,83576599	0,82892513	0,27520347	0,27843336	0,39497915
1,08672579	0,39044262	0,84374181	0,82200269	0,24839416	0,26486414	0,379683
1,14561312	0,46050358	0,86339263	0,8350045	0,28719252	0,31171243	0,39896888
1,13482525	0,44081903	0,93103053	0,91858164	0,20667557	0,21802318	0,48915105
1,09410454	0,39386204	0,88162551	0,86954724	0,21393411	0,2250891	0,44172301
1,11065285	0,41401867	0,89176262	0,87945763	0,2202257	0,23162353	0,42701277
1,12887767	0,46281096	0,87471855	0,87105825	0,25504675	0,2579685	0,41361388
1,14887058	0,45381999	0,8705824	0,86290503	0,28218966	0,28618468	0,37932533
1,14947157	0,46461619	0,8819004	0,87482866	0,27023393	0,27498097	0,40447951
1,13419205	0,45263364	0,85132501	0,82647868	0,2880028	0,30773335	0,3776145
1,11159663	0,44858388	0,88112523	0,84530204	0,23503988	0,26634617	0,4072018
1,12054106	0,47620963	0,87726625	0,84296348	0,26702937	0,28373093	0,38855466
1,0669566	0,36866211	0,84877883	0,82898701	0,21884341	0,23826599	0,45020554
1,13805192	0,43471746	0,86182087	0,84615552	0,28124026	0,29225399	0,40131793
1,13442307	0,46533688	0,83186874	0,81467385	0,31042224	0,32044088	0,42248085
1,11795683	0,48489107	0,82339761	0,82180315	0,30188611	0,2968723	0,37136387
1,08402188	0,45297486	0,81003669	0,80222363	0,27726701	0,28219899	0,3588083
1,16828779	0,46428519	0,8476116	0,84606235	0,32555475	0,3223419	0,37561402

1,14361239	0,39917359	0,82861379	0,807847	0,32097464	0,33610823	0,40876636
1,14796451	0,53080274	0,83128951	0,82344923	0,32170203	0,32477816	0,372078
1,17115162	0,39696936	0,82502896	0,80776665	0,35238977	0,36415773	0,36539085
1,11700899	0,46904156	0,8571628	0,84901324	0,26289364	0,26809299	0,36501588
1,11655925	0,41913278	0,85266602	0,84143494	0,26730923	0,27515268	0,44821812
1,13826243	0,429145	0,84404813	0,83127457	0,30006357	0,30735172	0,41039639
1,13926915	0,46139184	0,81883508	0,82290081	0,33114397	0,31795336	0,3814418
1,13202142	0,4687316	0,83525377	0,82692113	0,30011074	0,30510532	0,40927157
1,1312761	0,42187186	0,8912565	0,87779552	0,24503185	0,25350206	0,44486059
1,14552029	0,44681766	0,86802528	0,85589324	0,28163518	0,28965267	0,40035425
1,11775522	0,40679251	0,83445362	0,81610306	0,29112894	0,30174207	0,36783454
1,13194649	0,41866789	0,84043461	0,81946965	0,2977095	0,31279661	0,38971285
1,15642237	0,42175608	0,87446008	0,85382275	0,28675344	0,30261073	0,40306758
1,11738869	0,4373325	0,8450357	0,84395595	0,2770966	0,27344286	0,38730322
1,05903906	0,4151274	0,82938663	0,81779644	0,23278248	0,24129244	0,40107112
1,17389168	0,46666054	0,93337601	0,91625661	0,24535331	0,25769164	0,40579766
1,13381094	0,47261178	0,89908885	0,8888016	0,23630589	0,24540248	0,41805711
1,12264714	0,438329	0,87525653	0,86199231	0,25008984	0,26065967	0,42199123
1,09470215	0,38563679	0,84340136	0,82641559	0,25582982	0,26832743	0,39103477
1,10671307	0,35569724	0,92948697	0,92249217	0,180022	0,18533194	0,51716677
1,17333466	0,46585936	0,89260783	0,86581115	0,28479463	0,30752362	0,3846395
1,08671109	0,42965016	0,8330152	0,81718873	0,25782357	0,2698224	0,36901272
1,17191699	0,44440052	0,84110076	0,81629083	0,33591856	0,35598974	0,43308021
1,08814327	0,42359847	0,84085248	0,82033237	0,25192013	0,26781236	0,39226253
1,1311579	0,40457017	0,86820745	0,85159279	0,26576369	0,27957427	0,41652552
1,12491701	0,4398854	0,87133648	0,8546118	0,25942911	0,27031676	0,41402554
1,18061694	0,37726671	0,88792634	0,86252444	0,30115386	0,31837176	0,45505347
1,13694064	0,44310498	0,89705354	0,86735152	0,24159249	0,27023755	0,45256011
1,08108288	0,41883711	0,86771619	0,84135371	0,21796156	0,24029813	0,41048393
1,11630099	0,40867053	0,84910959	0,79434748	0,27346874	0,32195357	0,4441297
1,11511915	0,38609756	0,87009539	0,85368439	0,24805451	0,26143718	0,45406793
1,10713994	0,42312836	0,81294311	0,78446478	0,30213566	0,32297427	0,39772507
1,14645709	0,42372474	0,8736256	0,83687743	0,27703971	0,30959502	0,4021045
1,05943626	0,40229329	0,72562321	0,69862517	0,34148967	0,36223554	0,31355151
1,14915145	0,4169218	0,83733045	0,80434414	0,3160748	0,34495534	0,39713031
1,10086131	0,36703461	0,84932272	0,83078866	0,25335517	0,2703004	0,40825888
1,112479	0,41092894	0,85731849	0,83156246	0,25800235	0,2810646	0,39332501
1,11247894	0,43699087	0,8792967	0,86240012	0,23501106	0,25030984	0,44407598
1,10680219	0,49630631	0,95547295	0,93609257	0,15191981	0,17641989	0,48974092
1,1131369	0,41947822	0,87394922	0,82837253	0,24114493	0,28476439	0,43110947
1,15568729	0,53847383	0,88745726	0,84002227	0,27096177	0,31574051	0,42271727
1,07873589	0,42321945	0,85359583	0,81654974	0,22709529	0,2623842	0,46062688
1,13145663	0,46174195	0,85830245	0,83507371	0,27811428	0,29661802	0,40683025
1,14227326	0,4128089	0,77713781	0,779317	0,3748669	0,3636905	0,34023807
1,11161716	0,4709128	0,98719535	1,02039364	0,12442303	0,10371557	0,50671261
1,16810728	0,52852083	0,8841218	0,84948006	0,28932933	0,31865889	0,34715978
1,12152679	0,47322151	0,85866787	0,81930471	0,26770378	0,30228624	0,38901607
1,14722619	0,44209088	0,85248022	0,82588106	0,29799253	0,32142871	0,4250446
1,11011567	0,41001984	0,8643602	0,83836367	0,24614809	0,2732279	0,4131306
1,07903238	0,38183948	0,79052655	0,75197529	0,29451234	0,32729516	0,39667703

1,09681299	0,41530887	0,86895064	0,8814153	0,22822755	0,21816848	0,46256261
1,08076099	0,33397861	0,83894341	0,85927567	0,24278142	0,22414798	0,47398062
1,13126746	0,36807921	0,84822013	0,82150044	0,28468144	0,30983052	0,41335458
1,0918884	0,39907921	0,82811562	0,80188584	0,26826453	0,29009451	0,42977984
1,1001456	0,4187544	0,86065985	0,83094548	0,2417525	0,2704898	0,4168881
1,10121184	0,36319977	0,84375549	0,84354814	0,25785808	0,25905553	0,47072831
1,11927881	0,44447791	0,85185977	0,83342672	0,26995719	0,28589677	0,43453837
1,10936157	0,37588083	0,85561343	0,83064241	0,25558527	0,27893006	0,44167482
1,13524742	0,3828998	0,83462801	0,80740209	0,30343528	0,32784706	0,39622431
0,92805285	0,29482257	0,69924654	0,6561016	0,23609185	0,27200443	0,37265367
1,17817469	0,49138821	0,92797403	0,89265394	0,25537776	0,28552101	0,40734153
1,09735591	0,39551325	0,83105867	0,81179725	0,26880698	0,28556356	0,42969571
1,18418954	0,54982055	0,91454541	0,87495541	0,27429076	0,30936923	0,39703007
1,07778573	0,40325116	0,83013364	0,81699507	0,24857729	0,26247081	0,36894947
1,08872553	0,48706683	0,85981306	0,82511273	0,23192584	0,26363866	0,40289759
1,11802379	0,48459756	0,90752979	0,87161952	0,21228142	0,24709082	0,47315306
1,11314121	0,42758835	0,84142897	0,8336219	0,27376835	0,27955153	0,41547751
1,10428236	0,43156582	0,84517922	0,81352806	0,26396488	0,29081916	0,4078439
1,10568744	0,40180767	0,8972449	0,86571973	0,2098497	0,24093625	0,45394161
1,12768908	0,42934005	0,88562481	0,86799347	0,24411964	0,26029519	0,4410923
1,08528787	0,47034089	0,91204692	0,89261209	0,17389419	0,19509749	0,45688226
1,14255899	0,44350953	0,87278257	0,87112925	0,2719756	0,27174022	0,42297088
1,16467072	0,45435577	0,93103343	0,89392352	0,23433256	0,27180397	0,50097708
1,13652355	0,42332936	0,8938082	0,85905231	0,2459564	0,27759632	0,45443467
1,12357411	0,4294063	0,83237766	0,82230401	0,29481215	0,30129621	0,3846717
1,11609632	0,39084024	0,82161326	0,80673057	0,3008732	0,30937483	0,4056423
1,12017904	0,4562286	0,85365992	0,80376344	0,27099702	0,3164157	0,39314597
1,08992488	0,45746752	0,82645962	0,79647246	0,26817769	0,2936663	0,37932683
1,09641968	0,42631729	0,8839312	0,835812	0,21540199	0,26113774	0,40412154
1,12468981	0,4416687	0,869358	0,83235919	0,26016485	0,2925399	0,39962109
1,08993322	0,38950051	0,82212441	0,79945623	0,27046676	0,29053667	0,40613864
1,09192887	0,45173602	0,78987728	0,74714128	0,31033435	0,34513198	0,37731828
1,12906658	0,45000686	0,86315155	0,81750477	0,27245365	0,31156267	0,41463681
1,09095878	0,41273571	0,85370366	0,82184722	0,24002357	0,26919346	0,42605317
1,15784936	0,41735705	0,90167892	0,89006323	0,25753009	0,26839919	0,44841666
1,08375091	0,37255138	0,8298276	0,80486728	0,25640246	0,27925605	0,38546094
1,15481577	0,45592991	0,87838529	0,86463473	0,28259883	0,29018707	0,38927364
1,07546276	0,36547965	0,83519283	0,79113882	0,24621267	0,28438391	0,40350814
1,11935576	0,41274314	0,93203135	0,91878577	0,18759239	0,20249567	0,4673399
1,14140708	0,40289094	0,8257526	0,82261322	0,32131561	0,31889904	0,39916609
1,20218597	0,42375349	0,85084423	0,83415758	0,35787829	0,36867494	0,40298232
1,15903957	0,52581763	0,88421933	0,83792359	0,27695431	0,32115902	0,37106946
1,16857335	0,42497965	0,86072597	0,8387441	0,31226275	0,32999367	0,36812571
1,11959369	0,4738137	0,86547136	0,84921908	0,25616992	0,27042524	0,38409016
1,11587859	0,45330729	0,89661366	0,85769444	0,22054908	0,26018133	0,40866541
1,10197139	0,4268482	0,85276538	0,82858813	0,25329313	0,27436962	0,41137755
1,11603257	0,36247799	0,86585076	0,85377385	0,2512214	0,26234085	0,45429955
1,12141746	0,44779553	0,88145937	0,85443638	0,24174117	0,26738103	0,43506372
1,09260068	0,38142906	0,82166202	0,79857683	0,27429698	0,2940396	0,39433891
1,13047407	0,41697612	0,86994374	0,85581253	0,26226618	0,27480365	0,41849086

1,13599041	0,40243638	0,93217719	0,91608829	0,20494094	0,22015656	0,49854174
1,13026989	0,43875641	0,8481888	0,84290709	0,28592268	0,28746795	0,3985891
1,08653291	0,40253134	0,78370351	0,76428993	0,3085879	0,32304458	0,35255365
1,08207388	0,42777125	0,79816317	0,78614899	0,28755905	0,29603289	0,38211226
1,10273353	0,47164855	0,96160529	0,93408751	0,14119247	0,17182995	0,53703715
1,15235549	0,41789312	0,91700728	0,86510005	0,23698796	0,28739011	0,44415804
1,09565931	0,39391251	0,86000555	0,83123177	0,23925775	0,26443012	0,44556121
1,09041495	0,41217762	0,83162742	0,81875637	0,26143145	0,27169444	0,41087414
1,10092062	0,41927896	0,85214769	0,82891646	0,25058571	0,27227049	0,43593296
1,10080309	0,4171311	0,86914181	0,83442461	0,23393745	0,26671241	0,4171311
1,1309125	0,42405349	0,84487626	0,82930354	0,28966544	0,30168938	0,40824347
1,16132963	0,43530327	0,86182234	0,85781737	0,30169418	0,30362622	0,40891388
1,13231003	0,47186937	0,90499847	0,8692885	0,22985647	0,26307607	0,41796383
1,13033382	0,38911367	0,83339974	0,81581534	0,29807494	0,31456385	0,4477992
1,12097156	0,47246249	0,87155445	0,83321474	0,25109217	0,28826941	0,40673416
1,06563931	0,36983276	0,79102019	0,77385854	0,27945819	0,29277364	0,43188319
1,14481863	0,53592433	0,84505121	0,8220902	0,30379879	0,32273618	0,45088108
1,12912213	0,4251752	0,87442128	0,84986481	0,25816313	0,27928154	0,41885462
1,1066403	0,43037768	0,85665813	0,82943415	0,25565448	0,27733875	0,41556604
1,14398734	0,43921578	0,87775069	0,87159896	0,270016	0,27244634	0,40370091
1,13497215	0,46723554	0,86626008	0,85868267	0,27056607	0,27710674	0,42229196
1,07863842	0,43549326	0,82990062	0,81281627	0,25426635	0,26584604	0,41258687
1,14395635	0,41680317	0,86760835	0,8505352	0,28264581	0,29398075	0,45344922
1,19753175	0,46179034	0,90492781	0,86650091	0,29598812	0,33103565	0,42853503
1,0906852	0,44700946	0,83977168	0,82978488	0,25433594	0,26090095	0,39028868
1,14482102	0,38913661	0,82942408	0,82185851	0,31979819	0,323098	0,4203662
1,12543518	0,43871216	0,96299679	0,93774716	0,16348845	0,18877377	0,52422617
1,15022181	0,46329614	0,89041242	0,87715662	0,26184104	0,2732472	0,45119023
1,11331732	0,45605022	0,83730181	0,83473096	0,28111446	0,27868664	0,38828504
1,09735918	0,40676626	0,83940498	0,82205352	0,26171075	0,27538525	0,39564353
1,10294399	0,44126366	0,82243533	0,799481	0,28050956	0,30800522	0,36589262
1,12235696	0,44486499	0,83227943	0,83321479	0,2928142	0,28917787	0,37009812
1,13353551	0,4759995	0,88673538	0,86266496	0,2503514	0,27112943	0,41448112
1,09937713	0,41575706	0,83151848	0,825643	0,27190107	0,27375979	0,38405489
1,09625143	0,44712147	0,83123163	0,8237707	0,26795236	0,27252163	0,38840817
1,09412517	0,41725729	0,87805166	0,85882527	0,21805336	0,23540849	0,45254221
1,10703137	0,47039216	0,87149874	0,85805367	0,23961316	0,24909168	0,41199684
1,09187428	0,47124058	0,84969833	0,83808023	0,24583394	0,25390419	0,4100637
1,08226616	0,42732856	0,80517503	0,78965799	0,28037607	0,29261505	0,42730547
1,08583915	0,42675898	0,82052441	0,80945961	0,27139994	0,27664321	0,3897627
1,11368422	0,36461831	0,82128521	0,8132666	0,29503672	0,30044501	0,38164882
1,13202608	0,41562981	0,87457806	0,86219318	0,25919671	0,26983657	0,45099831
1,09418007	0,43163651	0,8289021	0,81493874	0,27093851	0,27959425	0,41605077
1,10883947	0,43227637	0,85747925	0,83967663	0,25866172	0,26933316	0,39535909
1,12880855	0,38943892	0,7969745	0,78869754	0,33776107	0,34026609	0,37666389
1,09731359	0,40751452	0,82990142	0,81653194	0,27378678	0,28100198	0,40867843
1,0762685	0,39531271	0,79091749	0,78634889	0,28835872	0,29003582	0,38114663
1,09158226	0,40686735	0,8116205	0,81250361	0,28184886	0,27907878	0,41423659
1,07366986	0,42570097	0,76472813	0,71083909	0,32089761	0,36491109	0,34191469
1,12186859	0,4374954	0,84161459	0,83133744	0,28267842	0,29053616	0,41744397

1,14276756	0,43029151	0,8474368	0,83565846	0,29999212	0,30717838	0,41673106
1,12028349	0,51116915	0,85577044	0,82830002	0,26955693	0,29212859	0,38246065
1,08935262	0,4294963	0,83751052	0,81554337	0,25809826	0,27392533	0,36446497
1,10630764	0,41255579	0,82872702	0,80995792	0,28181405	0,29642163	0,42244056
1,37474016	0,58353393	1,07738324	1,03603374	0,29971809	0,33904374	0,47562284
1,12933198	0,46996594	0,8349029	0,80908555	0,29750144	0,32025939	0,36324921
1,12315512	0,47920155	0,8577368	0,84569232	0,26827716	0,27754895	0,3893619
1,13431507	0,50830252	0,84997047	0,81238209	0,28725378	0,32194641	0,42015433
1,14094008	0,48699292	0,89579093	0,86510916	0,24997772	0,27583402	0,41280602
1,14958526	0,45612725	0,84746504	0,82829758	0,30653413	0,32137902	0,39094754
1,10770898	0,44179823	0,83369333	0,81891285	0,27680779	0,2887966	0,39784918
1,10135525	0,42238869	0,825569	0,820824	0,27972844	0,28055749	0,37506998
1,09530298	0,44934088	0,85255685	0,83276005	0,24440922	0,26255054	0,41199279
1,11065454	0,42379576	0,82169053	0,82732996	0,29244502	0,28335042	0,37663207
1,12119116	0,44081214	0,82284586	0,81469348	0,30394914	0,30660185	0,35931756
1,13283526	0,48141939	0,84058492	0,8292668	0,2974311	0,30371891	0,38992362
1,13665416	0,45869526	0,87325818	0,84540809	0,26626141	0,29145964	0,41857527
1,1104795	0,45680947	0,85627104	0,83863796	0,25756578	0,27193202	0,3960542
1,14645057	0,46231836	0,86746191	0,84266695	0,28751492	0,30425206	0,39016097
1,05772073	0,43348586	0,79092209	0,7796564	0,27002708	0,27810316	0,38214646
1,13979454	0,48984272	0,85454771	0,83595587	0,29084078	0,30428936	0,39351541
1,13643147	0,50120125	0,91494778	0,88961367	0,22340373	0,24738564	0,40160489
1,10245936	0,43012573	0,8215619	0,81492992	0,286176	0,28756014	0,37521822
1,11864815	0,45193265	0,84984342	0,83576802	0,27166205	0,28289692	0,39679674
0,85150706	0,14650596	0,54589094	0,51881042	0,32980599	0,34607188	0,3994528
1,04713482	0,40038961	0,79380068	0,77884551	0,25654213	0,26840789	0,37831379
1,07491417	0,44102168	0,82558999	0,81829817	0,25232077	0,25672624	0,38759999
1,04209926	0,39612162	0,82777703	0,79319186	0,21622609	0,25048949	0,42543975
1,05562321	0,34044328	0,76544282	0,73654986	0,29209306	0,31911372	0,42984446
1,09159726	0,44318762	0,84755294	0,82651869	0,2460175	0,2651101	0,40394864
1,16679286	0,44847804	0,83505919	0,81033831	0,33374017	0,35647655	0,39553479
1,15944984	0,45633766	0,96331679	0,95784229	0,19732764	0,20347044	0,53437696
1,13497848	0,4544507	0,89630001	0,89183455	0,24235174	0,24316283	0,45929225
1,09106693	0,42795719	0,813408	0,79961635	0,28368141	0,29182273	0,40095613
1,12218298	0,46597715	0,92493823	0,90875599	0,19813511	0,21642154	0,45987131
1,08258216	0,42250249	0,82035525	0,79228331	0,26474313	0,29049042	0,42246724
1,08715974	0,45186639	0,82739133	0,78382706	0,26479352	0,30341645	0,38658113
1,07972927	0,39799654	0,82824915	0,81161539	0,25383188	0,26811952	0,4084328
1,07369589	0,41234632	0,85996979	0,81818525	0,21578075	0,25553662	0,4585949
1,11934154	0,44753072	0,84924223	0,83766846	0,27326457	0,28181229	0,41591879
1,07799951	0,45904758	0,82228413	0,79210581	0,2587424	0,28590031	0,35799507
1,07975657	0,44870265	0,84676672	0,81052969	0,23552427	0,26922851	0,37641533
1,13622396	0,39879075	0,91670057	0,90268397	0,2195798	0,23574663	0,46485526
1,1144861	0,44104478	0,86864908	0,83712111	0,2470273	0,2775094	0,440336
1,04971122	0,4278371	0,77224128	0,73981688	0,28275335	0,31006819	0,34337641
1,05331444	0,43045779	0,75883849	0,73383253	0,29999609	0,32018439	0,32982141
1,13321049	0,44222	0,89181567	0,83932967	0,24462546	0,29388131	0,435017
1,1300927	0,41627825	0,85962448	0,82949272	0,27340569	0,30060006	0,45321534
1,09804283	0,4040222	0,7921509	0,76146238	0,31084272	0,33679513	0,37561623
1,09479673	0,36385572	0,81387108	0,79651993	0,28572374	0,29856496	0,39776905

1,03922216	0,39393898	0,83714617	0,80928585	0,20498176	0,22995013	0,42953913
1,04639167	0,41515679	0,82755353	0,81898104	0,21961652	0,22927738	0,39102136
1,13237434	0,42220736	0,82651298	0,79499444	0,31219144	0,33780576	0,38709574
1,12841021	0,43057264	0,8002966	0,78281062	0,3341911	0,34563692	0,35716158
1,11968328	0,4342808	0,85541602	0,84851074	0,26571759	0,27150297	0,40564716
1,10651753	0,43638305	0,81260053	0,79299689	0,29959455	0,31377318	0,36583439
1,12470899	0,42659779	0,85415994	0,82661173	0,27256681	0,29810603	0,40210398
1,11966379	0,50729028	0,8954878	0,87744942	0,2256491	0,24249636	0,40212248
1,15577832	0,43419062	0,87090921	0,85846959	0,28828763	0,29761134	0,42302348
1,09081318	0,41406284	0,85184572	0,83786002	0,24133368	0,25373701	0,39462859
1,11422709	0,4340776	0,85739433	0,82973252	0,26105513	0,2845837	0,40462724
1,08326712	0,42357113	0,81235104	0,80163812	0,27578778	0,28167653	0,37464681
1,20094022	0,53610482	0,88679147	0,86270116	0,31596307	0,33852196	0,42890711
1,13358491	0,51442924	0,90682224	0,89957777	0,22968055	0,2343161	0,41384372
1,09453249	0,41511193	0,9539701	0,93074821	0,14078946	0,16736666	0,50896446
1,13109816	0,48310026	0,85566384	0,81827411	0,27792187	0,31282642	0,3959986
1,11430712	0,46897969	0,88893648	0,84503482	0,22816664	0,26968358	0,3824844
1,1231177	0,47050605	0,82517163	0,77554416	0,30257569	0,34820196	0,3748613
1,07620527	0,41106627	0,8060532	0,79320239	0,27349674	0,28327541	0,39608684
1,11164664	0,38763095	0,82679402	0,805612	0,28679151	0,30675874	0,44252319
1,12717003	0,4825261	0,8837209	0,82706455	0,24668272	0,30066898	0,3833683
1,10024831	0,43380401	0,82675719	0,82029013	0,27750018	0,27996488	0,43316426
1,07195185	0,36849567	0,78944634	0,76714543	0,28741006	0,30508992	0,4302499
1,16289401	0,45074797	0,8787368	0,84965753	0,28771492	0,3133286	0,37775459
1,12739395	0,4933699	0,87480326	0,87551647	0,25890328	0,25190719	0,41654157
1,10532122	0,45063278	0,83127773	0,80101929	0,27533725	0,30451849	0,41692912
1,10443219	0,48511235	0,85912167	0,85004736	0,24656455	0,25485335	0,4319329
1,11350218	0,41858719	0,85206392	0,84734616	0,26378729	0,26623367	0,43800393
1,10689255	0,42819564	0,82620833	0,82313254	0,28355584	0,28377633	0,38174305
1,12466577	0,50823314	0,84062636	0,83158685	0,28674977	0,29307904	0,36913699
1,03749632	0,41360405	0,7747697	0,73832586	0,26702901	0,29917328	0,35867897
1,11775134	0,43024156	0,844412	0,82336082	0,27612253	0,29440666	0,41198373
1,14698348	0,4349037	0,87518169	0,86943679	0,27732245	0,27761853	0,42307468
1,06486433	0,41231129	0,80686656	0,79535951	0,25950984	0,26951017	0,4142477
1,09962687	0,4710476	0,86870502	0,83592306	0,234307	0,26428573	0,42057889
1,07142714	0,46867132	0,84389865	0,83185348	0,232261	0,23986949	0,38529754

13_to_16	14_to_15	14_to_16
0,41204091	0,402432	0,38135608
0,39974036	0,43657365	0,42808307
0,40062287	0,41622903	0,39211838
0,41231442	0,42925735	0,41640191
0,38661394	0,34693121	0,32131917
0,38970869	0,4207585	0,38929309
0,40147322	0,4081824	0,39066188
0,38318791	0,41627785	0,38640294
0,42149819	0,43058655	0,41350006
0,40746952	0,43871676	0,42908947
0,39386242	0,40638497	0,38853678
0,39075873	0,42868987	0,40791395
0,42202366	0,53586911	0,51120146
0,42610547	0,32614521	0,30834371
0,38847486	0,43117681	0,39166198
0,41711799	0,4133113	0,39136812
0,41454926	0,40973817	0,39746143
0,3872978	0,36603022	0,35114921
0,37066943	0,40943734	0,39388661
0,39489262	0,37302362	0,34852822
0,38522392	0,38542515	0,36609618
0,41220973	0,42363957	0,40336903
0,45649227	0,48068951	0,46188593
0,39995202	0,41188614	0,38731977
0,39658885	0,38944301	0,37275034
0,38890152	0,42159291	0,39789136
0,38872459	0,44569243	0,42090344
0,39417516	0,41890384	0,3996236
0,36914319	0,38993795	0,35996211
0,39887784	0,44297769	0,4142794
0,39222723	0,33110594	0,31040542
0,39087542	0,44321889	0,42791595
0,37645931	0,33552211	0,33141598
0,42953327	0,49748357	0,47424991
0,39452718	0,41068766	0,39675382
0,39380304	0,44743702	0,42376586
0,46798929	0,43673054	0,42325068
0,38021379	0,38770355	0,38040474
0,39091047	0,37946573	0,36164135
0,40398645	0,38205291	0,36698759
0,39224085	0,42722417	0,41051596
0,41244052	0,4769079	0,45860924
0,45208762	0,46499318	0,44437978
0,4014301	0,45582693	0,43155443
0,36023312	0,44674989	0,41712535
0,41575242	0,42034356	0,39896733
0,3902161	0,45796618	0,4116604
0,41810753	0,43285557	0,45716489
0,39330701	0,40990108	0,43414203

0,40188186	0,420638	0,39962486
0,39971509	0,42420266	0,40184818
0,40172588	0,28307719	0,25772955
0,44913619	0,43956474	0,42731488
0,42908426	0,43837659	0,41310651
0,36941415	0,41311152	0,38448594
0,39034958	0,44457814	0,39395392
0,39252375	0,44702485	0,42639281
0,35324045	0,43436832	0,39805357
0,37282857	0,37837356	0,36359484
0,39761696	0,41528729	0,39825328
0,3905461	0,39714878	0,37565868
0,38679745	0,4002478	0,37931522
0,41106561	0,39299346	0,37321072
0,3712679	0,51414227	0,50589346
0,36056487	0,43475284	0,39039789
0,33222338	0,42279304	0,37482437
0,36281793	0,44852732	0,42201371
0,38268698	0,41723955	0,39226301
0,37790223	0,39845095	0,37434583
0,41672038	0,45494948	0,42209186
0,3916955	0,3925636	0,37980059
0,35227051	0,37292054	0,34059576
0,36531784	0,41280182	0,3855074
0,37757339	0,42094707	0,38851102
0,40002286	0,42698562	0,40667451
0,38748019	0,43636403	0,41657493
0,41207642	0,4157854	0,38176257
0,36420119	0,38694417	0,37812977
0,40064234	0,44878756	0,42500071
0,41920086	0,41663681	0,39440571
0,43523214	0,46403032	0,43980016
0,43590106	0,46462616	0,43417398
0,36356444	0,4353107	0,38995386
0,41290915	0,4277647	0,38658319
0,39501664	0,42641282	0,39294462
0,3021654	0,32246516	0,28758899
0,41032701	0,39598756	0,38117345
0,40743055	0,41824587	0,39289478
0,35045932	0,41052869	0,37164402
0,37293143	0,45332849	0,4200282
0,40359681	0,47868834	0,42477793
0,41393901	0,42497098	0,40147718
0,3924504	0,45707174	0,4088174
0,36759743	0,44832953	0,39887109
0,40608755	0,45393949	0,39755449
0,41475913	0,406359	0,39002545
0,39092095	0,39914263	0,3838144
0,39861872	0,40889932	0,38602176
0,38256109	0,37859217	0,35898952

0,4547193	0,46794325	0,44987273
0,44404117	0,50425154	0,45849974
0,42618756	0,47864884	0,45525443
0,40671318	0,39951985	0,37713608
0,37587208	0,4185647	0,38124005
0,36771999	0,36107886	0,3362078
0,40925076	0,4251164	0,40671072
0,40246386	0,42307134	0,4076991
0,39601574	0,44235776	0,4261249
0,41003019	0,4386101	0,41636694
0,38897848	0,39957052	0,36420171
0,36670525	0,41843067	0,39233202
0,37283463	0,39367869	0,35794625
0,36660501	0,4273627	0,40231723
0,40568637	0,41858277	0,37888512
0,44151681	0,46619063	0,44548958
0,41434978	0,43451693	0,42013771
0,37030539	0,39471861	0,37810997
0,39269214	0,41288708	0,39080788
0,40311214	0,45243202	0,42155151
0,38257782	0,44052839	0,41013076
0,39937589	0,43425661	0,40294138
0,34278427	0,39578117	0,35814471
0,38074774	0,41472581	0,38729636
0,36541519	0,38334193	0,36650268
0,42222861	0,45857255	0,4440051
0,44207067	0,40173163	0,37698642
0,33807184	0,41798186	0,39075461
0,3963112	0,45424465	0,42244795
0,40848983	0,43116373	0,43105641
0,38923007	0,4062325	0,36921321
0,41987705	0,42321016	0,40693294
0,39699935	0,38456335	0,37376591
0,37530851	0,45778222	0,43243042
0,37941227	0,40661152	0,37636709
0,49368571	0,49146971	0,47777696
0,44065355	0,49006428	0,47624074
0,42340106	0,48148941	0,46684955
0,41712751	0,4157699	0,40994188
0,38640921	0,42252945	0,41041356
0,40956651	0,42363216	0,41217477
0,36871267	0,40427261	0,37543314
0,38429767	0,43637706	0,39747631
0,36307414	0,41702017	0,37538595
0,43896271	0,48120956	0,46057395
0,39585522	0,42890279	0,41172846
0,41887891	0,3756541	0,35302921
0,38072851	0,34391033	0,33831519
0,36428335	0,36356125	0,35062688
0,38582634	0,38863771	0,38329617

0,40092931	0,43424597	0,41031243
0,3752459	0,31133017	0,29716824
0,35919177	0,43380939	0,41329507
0,36483857	0,39343467	0,38240722
0,44976698	0,4355032	0,42249491
0,40826087	0,41999531	0,4039193
0,39942397	0,36330068	0,36292534
0,41200798	0,36920606	0,35858082
0,44501618	0,471453	0,45607862
0,40167598	0,42823474	0,41149009
0,36639737	0,43180344	0,40998246
0,38123454	0,42515886	0,40159611
0,39469253	0,4567422	0,43312897
0,39837111	0,41407427	0,40877607
0,40172921	0,42072971	0,40532216
0,40194096	0,47052312	0,4503194
0,41805711	0,43482249	0,42018936
0,41826498	0,44020069	0,42468712
0,38802265	0,46021161	0,44106871
0,52358355	0,58305054	0,58202997
0,37118572	0,43217867	0,40169221
0,36254612	0,40782321	0,38949202
0,41924769	0,40012131	0,37279568
0,3845836	0,42285306	0,39851094
0,40864061	0,46489414	0,4471931
0,41179726	0,43611521	0,41563936
0,44392139	0,51212433	0,48529714
0,43460295	0,45664682	0,42469361
0,40055629	0,45247072	0,42292019
0,40766312	0,44291287	0,38583397
0,44684471	0,48685327	0,46856734
0,38429586	0,3972964	0,36379522
0,37820142	0,45538638	0,41494292
0,29825929	0,32990907	0,29909834
0,37595919	0,42350182	0,38827127
0,40043584	0,48426241	0,46416438
0,38053571	0,44890027	0,42103149
0,43873005	0,4464501	0,42686481
0,48383903	0,46309673	0,44028368
0,39547769	0,45589929	0,40923652
0,38880209	0,3528517	0,30224464
0,43584094	0,43153614	0,39338114
0,39448206	0,40065952	0,37461591
0,35765689	0,3693413	0,36726309
0,54962895	0,51925101	0,55003233
0,32615528	0,36467611	0,32421183
0,36217765	0,39050977	0,34766446
0,41136164	0,41456487	0,38469931
0,40042509	0,45500987	0,42834567
0,3715634	0,41216743	0,37099495

0,46358933	0,45391636	0,4682945
0,47993927	0,50498554	0,52652469
0,39679933	0,4817851	0,4537972
0,41559661	0,4325894	0,40390696
0,40265001	0,44388289	0,4122194
0,4808997	0,48117942	0,48034909
0,42370506	0,41076069	0,39027783
0,42838445	0,48029326	0,45476267
0,38036155	0,45443757	0,4252859
0,35056986	0,40446278	0,36289118
0,38718165	0,44344639	0,40362614
0,42162339	0,43760775	0,41672651
0,3735514	0,37705305	0,32956471
0,36715896	0,4320108	0,41519911
0,37903036	0,37988781	0,34102275
0,44789475	0,42840358	0,38879175
0,41698842	0,4160114	0,40647611
0,38808071	0,41700571	0,38277956
0,43362108	0,49898874	0,4649479
0,43646397	0,45781438	0,43870641
0,44948719	0,44382114	0,42246638
0,43225741	0,43055603	0,42763496
0,47469101	0,47789777	0,43963085
0,43306768	0,47188056	0,43577503
0,38748741	0,41023752	0,39554727
0,40809935	0,43435904	0,41626413
0,35792967	0,40225021	0,34883052
0,36063393	0,37386998	0,34075951
0,3718386	0,45926956	0,40954033
0,38121553	0,43316149	0,39181375
0,39640046	0,43546293	0,4106088
0,35214153	0,34239883	0,29593627
0,38646097	0,41792482	0,36837248
0,4079534	0,44221309	0,40916344
0,4473118	0,4857259	0,4728124
0,37412456	0,46037367	0,43295584
0,39102429	0,42789535	0,40996119
0,3791314	0,47161962	0,42572219
0,46399317	0,51990654	0,50604745
0,41088891	0,4264976	0,42045059
0,39659375	0,43057491	0,41129571
0,33712918	0,36391766	0,31410964
0,3572609	0,44378254	0,41783247
0,37747378	0,39475431	0,37621434
0,38500735	0,4475867	0,40516508
0,39420618	0,42787006	0,40264069
0,45005743	0,50398665	0,49136154
0,41970949	0,43729616	0,40759829
0,38335023	0,44343755	0,41817892
0,41480399	0,45422331	0,43894956

0,49126014	0,53080099	0,51385032
0,40687489	0,41312287	0,40470018
0,34289184	0,3837257	0,36241635
0,38445574	0,37368185	0,35871688
0,51740907	0,49160832	0,46291096
0,40270535	0,50020096	0,4473287
0,42807661	0,46750633	0,43748467
0,40634501	0,42236551	0,40763008
0,42583632	0,43432786	0,40974408
0,39635441	0,45314197	0,41730826
0,40380193	0,42255539	0,4055062
0,41192174	0,42791026	0,42286995
0,39350269	0,43655204	0,39843595
0,4386031	0,44598593	0,42718125
0,3825568	0,40269868	0,36148837
0,42316869	0,42255684	0,4044108
0,44155781	0,31808962	0,2892638
0,40798055	0,45138241	0,42497243
0,40144971	0,4304765	0,40029768
0,40942466	0,44244983	0,43324169
0,42827931	0,40297834	0,39207821
0,4094902	0,3987642	0,37830914
0,44676647	0,4543659	0,43488936
0,40316309	0,44535433	0,40499618
0,39264523	0,39741312	0,38423408
0,42371727	0,44308149	0,43339988
0,50835171	0,52698116	0,49984994
0,44925793	0,43024349	0,41458912
0,39753601	0,38690895	0,38044217
0,391445	0,43670636	0,41624476
0,36702277	0,38295539	0,3648522
0,38066889	0,39253898	0,38994706
0,398012	0,41393654	0,38794657
0,3895612	0,41999583	0,41121045
0,39217454	0,39110048	0,37934746
0,44256193	0,46505939	0,44336222
0,41289832	0,40624098	0,38886065
0,40723483	0,38405785	0,36926617
0,42421275	0,38557746	0,3655252
0,39028102	0,39681215	0,38326215
0,38272664	0,46133089	0,45090395
0,44664149	0,45977038	0,44664149
0,41364993	0,40125501	0,38451276
0,39190158	0,42681082	0,40742975
0,38256172	0,40998675	0,39949566
0,40854927	0,42549801	0,40962106
0,38476519	0,39938147	0,39255717
0,42287488	0,40705091	0,40631616
0,30241234	0,3484039	0,28936089
0,41599206	0,4081613	0,39540309

0,41653978	0,42123665	0,40648136
0,36662777	0,35333999	0,32083172
0,35412018	0,41322037	0,38759052
0,41637575	0,420658	0,39895843
0,44884318	0,49840363	0,45363684
0,34969789	0,37324607	0,34252688
0,38798712	0,38634464	0,36948273
0,39302013	0,34731287	0,30623467
0,39411551	0,41497888	0,38001102
0,38246306	0,39425303	0,37270022
0,39344612	0,39508523	0,37792884
0,38204183	0,40646256	0,39894405
0,39927258	0,40713759	0,38514593
0,39385767	0,40061942	0,40387403
0,3635204	0,38697804	0,37509409
0,38962057	0,36762607	0,35119323
0,40302582	0,41880846	0,38766171
0,39091786	0,40220771	0,38214841
0,37846847	0,41053642	0,38174357
0,38081829	0,36208975	0,34783446
0,38610097	0,36794496	0,34682687
0,38836705	0,41663611	0,38883144
0,38344275	0,3941579	0,38508294
0,39141288	0,40276757	0,38581646
0,38728692	0,43298568	0,41060995
0,37554976	0,39576776	0,37867983
0,39195333	0,39121845	0,37960685
0,40675887	0,4362768	0,39801547
0,40847173	0,42756402	0,39725392
0,39334072	0,40780564	0,38450635
0,3787427	0,38994638	0,36314386
0,53923058	0,50899319	0,50170898
0,46409232	0,44377008	0,43769615
0,40838624	0,38741651	0,37171929
0,45430351	0,46494178	0,44445905
0,40165117	0,40197236	0,37200925
0,3582025	0,38052611	0,33276043
0,40089881	0,43215696	0,41412457
0,42708491	0,44950658	0,40632566
0,41282593	0,40677077	0,39251041
0,33942242	0,37025895	0,33594234
0,35018069	0,40228252	0,36347067
0,4601797	0,5183789	0,50389323
0,41704094	0,42986938	0,39672527
0,3216795	0,35140851	0,31466105
0,31605122	0,33313676	0,30513303
0,3941809	0,45280416	0,39808598
0,4335899	0,4446127	0,41331216
0,35729329	0,39156376	0,3583261
0,391431	0,45099869	0,43272526

0,41237917	0,443807	0,41536088
0,3940459	0,41437161	0,40395399
0,36954179	0,40665701	0,37311866
0,35646771	0,37241624	0,35234271
0,41000201	0,42606661	0,4160489
0,3590577	0,38157599	0,35831375
0,38529815	0,43032876	0,4008889
0,39259454	0,39431225	0,37270325
0,42525868	0,44240999	0,42572838
0,39392067	0,44214799	0,42476187
0,38928946	0,42675338	0,39671013
0,37982421	0,39391215	0,37907974
0,4165697	0,35934869	0,32979999
0,41949484	0,3993804	0,38738028
0,49682368	0,54044163	0,51584769
0,37247233	0,37535603	0,33578143
0,35283139	0,42370027	0,37673508
0,33517333	0,3613589	0,3087268
0,39135281	0,39755466	0,38303952
0,4256426	0,43930777	0,41800177
0,34667169	0,40523823	0,34500939
0,43913245	0,39526098	0,38666855
0,41969524	0,4248906	0,40010358
0,36085283	0,43256323	0,40079478
0,43092522	0,38913804	0,38445518
0,39908874	0,38256043	0,35064633
0,43275353	0,37830591	0,36616259
0,44340308	0,43599218	0,42928415
0,3904723	0,40100852	0,39554714
0,36970083	0,34212532	0,3279144
0,33851672	0,36856957	0,32736902
0,40131392	0,41608439	0,39350074
0,42911097	0,44410599	0,43544101
0,41032109	0,3970802	0,38408276
0,40586401	0,40187231	0,36542377
0,38903292	0,37751007	0,36319184
