# Supplementary material for: Cross-Mating Compatibility and Competitiveness among Aedes albopictus Strains from Distinct Geographic Origins - Implications for Future Application of SIT Programs in the South West Indian Ocean Islands
Source: PLoS One. 2016 Nov 2;11(11):e0163788. doi: 10.1371/journal.pone.0163788 (PMC5091895; doi:10.1371/journal.pone.0163788)
Supplement: S1 Table — (PDF) [file pone.0163788.s003.pdf]

rep	Mating	ber of collected fe	ber of female disse	er of female insem	egg numbe	atched egg numbe	hatching rate	egg production/female
1	Run_Run	44	44	44	854	836	97,89227166	19,4090909
1	Run_Sey	47	47	47	1068	952	89,13857678	22,7234043
1	Run_Mau	44	44	44	1011	958	94,75766568	22,9772727
1	Sey_Run	31	31	29	480	466	97,08333333	15,483871
1	Sey_Sey	45	41	37	872	809	92,77522936	19,3777778
1	Sey_Mau	45	45	45	1187	1142	96,20893008	26,3777778
1	Mau_Run	32	32	32	592	588	99,32432432	18,5
1	Mau_Sey	43	43	43	1250	1145	91,6	29,0697674
1	Mau_Mau	43	41	40	1025	677	66,04878049	23,8372093
2	Run_Run	48	48	46	851	788	92,59694477	17,7291667
2	Run_Sey	50	47	46	913	802	87,8422782	18,26
2	Run_Mau	41	41	41	1024	800	78,125	24,9756098
2	Sey_Run	38	38	34	646	415	64,24148607	17
2	Sey_Sey	40	34	33	570	517	90,70175439	14,25
2	Sey_Mau	48	39	38	319	224	70,21943574	6,64583333
2	Mau_Run	43	42	40	704	493	70,02840909	16,372093
2	Mau_Sey	38	30	30	555	507	91,35135135	14,6052632
2	Mau_Mau	41	41	39	802	615	76,68329177	19,5609756
3	Run_Run	14	14	14	252	231	91,66666667	18
3	Run_Sey	46	46	45	1208	1168	96,68874172	26,2608696
3	Run_Mau	49	41	38	1355	1168	86,19926199	27,6530612
3	Sey_Run	48	30	30	755	617	81,7218543	15,7291667
3	Sey_Sey	40	23	23	658	410	62,3100304	16,45
3	Sey_Mau	44	41	40	1057	799	75,59129612	24,0227273
3	Mau_Run	47	46	45	676	646	95,56213018	14,3829787
3	Mau_Sey	56	30	30	1450	1007	69,44827586	25,8928571
3	Mau_Mau	46	46	45	661	534	80,78668684	14,3695652
