# Supplementary material for: Cross-Mating Compatibility and Competitiveness among Aedes albopictus Strains from Distinct Geographic Origins - Implications for Future Application of SIT Programs in the South West Indian Ocean Islands
Source: PLoS One. 2016 Nov 2;11(11):e0163788. doi: 10.1371/journal.pone.0163788 (PMC5091895; doi:10.1371/journal.pone.0163788)
Supplement: S2 Table — (PDF) [file pone.0163788.s004.pdf]

replic	Mating	dissected female number	inseminated female number	insemination rate
1	Run*_Run_Run	44	42	95,45454545
1	Sey*_Run_Run	30	30	100
1	Mau*_Run_Run	30	30	100
1	Run*_Sey_Sey	30	30	100
1	Sey*_Sey_Sey	35	33	94,28571429
1	Mau*_Sey_Sey	30	30	100
1	Run*_Mau_Mau	30	30	100
1	Sey*_Mau_Mau	30	30	100
1	Mau*_Mau_Mau	30	30	100
1	Run*_Run	30	30	100
1	Run*_Sey	30	30	100
1	Run*_Mau	30	30	100
1	Sey*_Run	30	30	100
1	Sey*_Sey	42	41	97,61904762
1	Sey*_Mau	49	47	95,91836735
1	Mau*_Run	30	30	100
1	Mau*_Sey	39	38	97,43589744
1	Mau*_Mau	30	30	100
1	Run_Run	30	30	100
1	Sey_Sey	30	30	100
1	Mau_Mau	30	30	100
2	Run*_Run_Run	41	39	95,12195122
2	Sey*_Run_Run	33	31	93,93939394
2	Mau*_Run_Run	30	30	100
2	Run*_Sey_Sey	37	35	94,59459459
2	Sey*_Sey_Sey	30	30	100
2	Mau*_Sey_Sey	30	30	100
2	Run*_Mau_Mau	30	30	100
2	Sey*_Mau_Mau	30	30	100
2	Mau*_Mau_Mau	30	30	100
2	Run*_Run	30	30	100
2	Run*_Sey	30	30	100
2	Run*_Mau	30	30	100
2	Sey*_Run	30	30	100
2	Sey*_Sey	30	30	100
2	Sey*_Mau	30	30	100
2	Mau*_Run	30	30	100
2	Mau*_Sey	30	30	100
2	Mau*_Mau	33	33	100
2	Run_Run	30	30	100
2	Sey_Sey	39	38	97,43589744
2	Mau_Mau	30	30	100
3	Run*_Run_Run	16	16	100
3	Sey*_Run_Run	26	26	100
3	Mau*_Run_Run	22	22	100
3	Run*_Sey_Sey	24	24	100
3	Sey*_Sey_Sey	22	22	100
3	Mau*_Sey_Sey	30	30	100
3	Run*_Mau_Mau	26	26	100

3	Sey*_Mau_Mau	38	36	94,73684211
3	Mau*_Mau_Mau	23	20	86,95652174
3	Run*_Run	30	29	96,66666667
3	Run*_Sey	37	33	89,18918919
3	Run*_Mau	28	27	96,42857143
3	Sey*_Run	27	27	100
3	Sey*_Sey	30	30	100
3	Sey*_Mau	43	42	97,6744186
3	Mau*_Run	22	22	100
3	Mau*_Sey	30	29	96,66666667
3	Mau*_Mau	20	20	100
3	Run_Run	27	27	100
3	Sey_Sey	10	10	100
3	Mau_Mau	16	16	100
